# Synthesis of Four Enantiomers of (1-Amino-3-Hydroxypropane-1,3-Diyl)Diphosphonic Acid as Diphosphonate Analogues of 4-Hydroxyglutamic Acid

**DOI:** 10.3390/molecules27092699

**Published:** 2022-04-22

**Authors:** Liwia Lebelt, Iwona E. Głowacka, Dorota G. Piotrowska

**Affiliations:** Bioorganic Chemistry Laboratory, Faculty of Pharmacy, Medical University of Lodz, Muszynskiego 1, 90-151 Lodz, Poland; liwia.lebelt@umed.lodz.pl (L.L.); iwona.glowacka@umed.lodz.pl (I.E.G.)

**Keywords:** Abramov reaction, phosphonates, glutamic acid analogues, absolute configuration

## Abstract

All the enantiomers of (1-amino-3-hydroxypropane-1,3-diyl)diphosphonic acid, newly design phosphonate analogues of 4-hydroxyglutamic acids, were obtained. The synthetic strategy involved Abramov reactions of diethyl (*R*)- and (*S*)-1-(*N*-Boc-amino)-3-oxopropylphosphonates with diethyl phosphite, separation of diastereoisomeric [1-(*N*-Boc-amino)-3-hydroxypropane-1,3-diyl]diphosphonates as *O*-protected esters, followed by their hydrolysis to the enantiomeric phosphonic acids. The absolute configuration of the enantiomeric phosphonates was established by comparing the ^31^P NMR chemical shifts of respective (*S*)-*O*-methylmandelic acid esters obtained from respective pairs of *syn*- and *anti*-[1-(*N*-Boc-amino)-3-hydroxypropane-1,3-diyl]diphosphonates according to the Spilling rule.

## 1. Introduction

**Figure 1:**
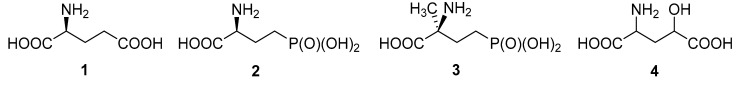
L-Glutamic acid **1** and its active analogues.

As analogues of naturally occurring α-amino acids, α-aminophosphonic acids are pharmacologically significant as they can mimic transition states of several biological processes such as peptide hydrolysis. Owing to the tetrahedral structure of the phosphonic residue, they can act as enzyme inhibitors or antibiotics [1,2,3,4]. Moreover, their activity often depends on the absolute configuration at Cα in α-aminophosphonic acids. Over decades, a vast number of phosphonate analogues of α-amino acids have been synthesized with the intention to study their biological properties (Figure 1). Among them, analogues of glutamic acid **1**, a major excitatory neurotransmitter in the central nervous system, deserve great consideration. For example, 2-amino-4-phosphonobutanic acid (L-AP4) **2** has been obtained as an analogue of glutamic acid and appeared to be a selective agonist for group III glutamate metabotropic receptors (mGluR) [5,6,7,8,9], whereas its α-methylated analogue (MAP4) **3** acts as a competitive antagonist of mGluR [10,11].

In continuation of our research program directed at the syntheses of enantiomerically pure functionalized aminophosphonates, we focus attention on hydroxyglutamic acids, which are widely available in nature, including plants, however this structure is also found as a part of more complex molecules with important biological properties. As expected, the presence of an additional hydroxy group in the glutamic acid framework may have a positive impact on the activity of its analogues. Thus, (2*S*,4*S*)-4-hydroxyglutamic acid **4** exhibited potency at mGlu_1a_R and mGlu_8a_R similar to that of L-glutamic acid [12], and its isomer (2*S*,4*R*)-**4** demonstrated a significant preference for the NMDA (N-methyl-D-aspartic acid) receptor [13].

**Figure 2:**
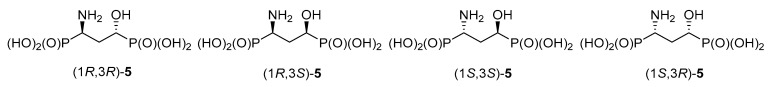
Four enantiomers of (1-amino-3-hydroxypropane-1,3-diyl)diphosphonic acid **5**.

Inspired by these observations we considered the synthesis of all four enantiomerically pure diphosphonic acids **5** (Figure 2).

**Scheme 1:**
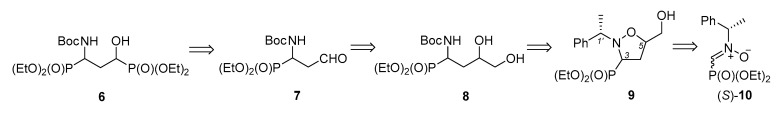
Retrosynthesis of [1-(*N*-Boc-amino)-3-hydroxypropane-1,3-diyl]diphosphonate **6**.

Our synthetic strategy relied on the formation of the C–P bond by the addition of diethyl phosphite to (*R*)- and (*S*)-(1-amino-2-oxoethyl)phosphonates **7**, available from the enantiomerically pure *N*-(1-phenylethyl)-*C*-(diethoxyphosphoryl)nitrone (*S*)-**10** already described by our research group (Scheme 1) [14].

## 2. Results and Discussion

**Scheme 2:**
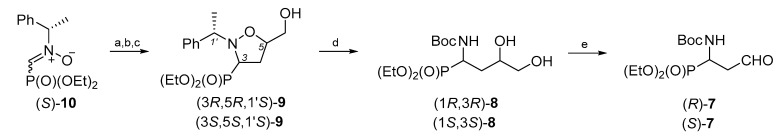
Synthesis of the aldehydes (*R*)- and (*S*)-**7**. Reagents and conditions: (a) allyl alcohol, MgBr_2_-etherate, 24 h, rt; (b) Ac_2_O, NEt_3_, DMAP, rt, 24 h, chromatographic separation; (c) NH_4_OH, EtOH, rt, 4h; (d) Boc_2_O, H_2_, 20% Pd(OH)_2_/C, EtOH, rt, 24 h; (e) NaIO_4_, CH_2_Cl_2_–H_2_O, rt, 2 h [14,15].

The enantiomerically pure aldehydes (*R*)-**7** and (*S*)-**7** were synthesized starting from the nitrone (*S*)-**10** following the reaction sequence depicted in Scheme 2, and their configurational stability was proven [14,15]. Cycloaddition of the nitrone (*S*)-**10** to allyl alcohol in the presence of MgBr_2_ led to the formation of an inseparable 1:1 mixture of isoxazolidines (3*R*,5*R*,1′*S*)-**9** and (3*S*,5*S*,1′*S*)-**9**. They were successfully separated as *O*-acetyl derivatives from which the starting compounds (3*R*,5*R*,1′*S*)-**9** and (3*S*,5*S*,1′*S*)-**9** were recovered after ammonolysis. Subsequent catalytic hydrogenation in the presence of Boc_2_O produced the *N*-Boc-aminodiols (1*R*,3*R*)-**8** and (1*S*,3*S*)-**8,** respectively, which upon treatment with sodium metaperiodate, gave the aldehydes (*R*)-**7** and (*S*)-**7**.

**Scheme 3:**
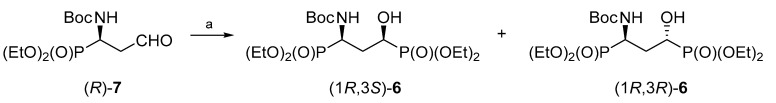
Synthesis of the diphosphonates (1*R*,3*S*)-**6** and (1*R*,3*R*)-**6**. Reagents and conditions: (a) HP(O)(OEt)_2_, NEt_3_, rt, 48 h.

The aldehyde (*R*)-**7** was subjected to the Abramov reaction with diethyl phosphite in the presence of catalytic amounts of triethylamine to afford a 1:1 mixture of diastereoisomeric diphosphonates (1*R*,3*S*)-**6** and (1*R*,3*R*)-**6** (Scheme 3). Attempts to separate the diastereoisomeric mixture of diphosphonates by column (silica gel) and high performance liquid chromatography (HPLC) appeared fruitless as the fractions collected were only enriched for the respective isomers (up to 90%). The ratio of diastereoisomers was established on the basis of ^31^P NMR spectra of the crude product. Since two phosphonyl groups are installed in the structure of compound **6**, two signals were identified for each of the respective diastereoisomeric diphosphonates (1*R*,3*S*)-**6** (δ^31^P = 25.26 and 23.58 ppm) and (1*R*,3*R*)-**6** (δ^31^P = 24.60 and 24.01 ppm).

**Scheme 4:**
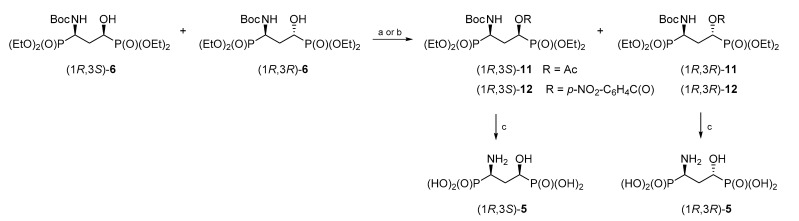
Reagents and conditions: (a) Ac_2_O, Et_3_N, DMAP, 2 h, rt; (b) *p*-NO_2_-C_6_H_4_C(O)Cl, Et_3_N, DMAP, CH_2_Cl_2_, 4 h, rt; (c) 5M HCl, 6 h, reflux, propylene oxide [81% from (1*R*,3*S*)-**11** and 84% from (1*R*,3*R*)-**11**; 53% from (1*R*,3*S*)-**12** and 60% from (1*R*,3*R*)-**12**].

Separation of the diastereoisomeric mixture of 3-hydroxydiphosphonates **6** was achieved by their transformation into *O*-protected derivatives (Scheme 4). Thus, a 1:1 mixture of compounds (1*R*,3*S*)-**6** and (1*R*,3*R*)-**6** was esterified with acetic anhydride in the presence of triethylamine and catalytic amounts of DMAP (4-dimethylaminopyridine) to form the *O*-acetyl derivatives (1*R*,3*S*)-**11** and (1*R*,3*R*)-**11**, which were then successfully separated by HPLC into a faster eluting diastereoisomer (1*R*,3*S*)-**11** (22%) and a late-eluting one (1*R*,3*R*)-**11** (40%). Alternatively, a 1:1 mixture of diphosphonates (1*R*,3*S*)-**6** and (1*R*,3*R*)-**6** was benzoylated with *p*-nitrobenzoyl chloride to produce the derivatives (1*R*,3*S*)-**12** and (1*R*,3*R*)-**12**, and their separation by HPLC allowed isolation of pure isomer (1*R*,3*S*)-**12** (21%) followed by (1*R*,3*R*)-**12** (31%). Finally, the *O*-protected derivatives **11** and **12** were efficiently hydrolysed to produce the phosphonic acids (1*R*,3*S*)-**5** and (1*R*,3*R*)-**5**.

**Scheme 5:**
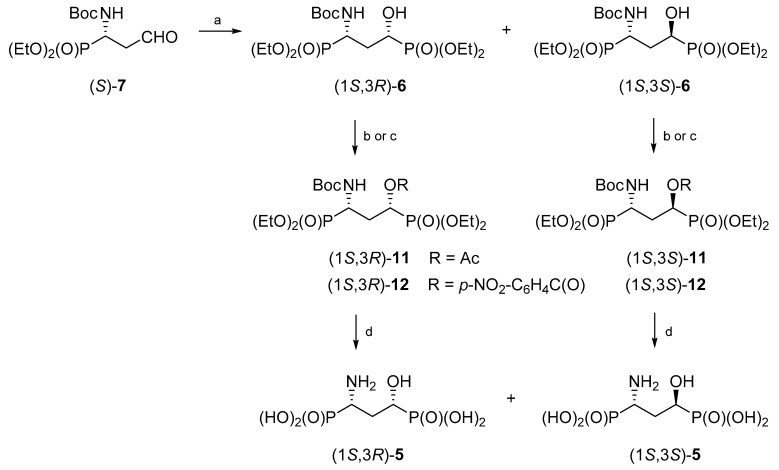
Synthesis of the diphosphonates (1*S*,3*R*)-**6** and (1*S*,3*S*)-**6**. Reagents and conditions: (a) HP(O)(OEt)_2_, Et_3_N, rt, 48 h; (b) Ac_2_O, Et_3_N, DMAP, 2 h, rt [20% for (1*S*,3*R*)-**12** and 32% for (1*S*,3*S*)-**12**]; (c) *p*-NO_2_-C_6_H_4_C(O)Cl, Et_3_N, DMAP, CH_2_Cl_2_, 4 h, rt [17% for (1*S*,3*R*)-**13** and 14% for (1*S*,3*S*)-**13**; (d) 5M HCl, 6 h, reflux, propylene oxide [86% for (1*S*,3*R*)-**5** and 69% for (1*S*,3*S*)-**5**].

To complete the full set of stereoisomeric phosphonic acids **5**, the aldehyde (*S*)-**7** was used to synthesize diphosphonates (1*S*,3*R*)-**6** and (1*S*,3*S*)-**6**, which were subsequently *O*-protected as the respective esters **11** or **12,** and then transformed into the final acids (1*S*,3*R*)-**5** and (1*S*,3*S*)-**5** by application of an analogous reaction sequence (Scheme 5).

Since enantiomerically pure aldehydes were used for the synthesis of the respective diphosphonates, i.e., (*R*)-**7** to obtain (1*R*,3*S*)-**6** and (1*R*,3*R*)-**6**, and (*S*)-**7** to obtain (1*S*,3*R*)-**6** and (1*S*,3*S*)-**6**, the absolute configuration at C1 in the isomeric compounds **6** can be arbitrarily assigned. In order to unambiguously determine the absolute configuration at C3, it was therefore necessary to establish the relative configuration between C1 and C3 for the diastereoisomeric pairs of the respective diphosphonates, namely, (1*R*,3*S*)-**6** and (1*R*,3*R*)-**6**, and (1*S*,3*R*)-**6** and (1*S*,3*S*)-**6**.

**Scheme 6:**
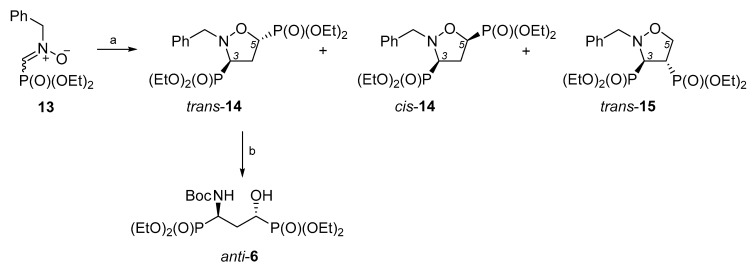
Cycloaddition of nitrone **13** to vinylphosphonate and transformation of cycloadduct *trans*-**14** into *anti*-**5**. Reagents and conditions: (a) vinylphosphonate, 60 °C, 48 h; (b) H_2_, 20% Pd(OH)_2_/C, 48 h, rt.

In assigning the relative configurations of the diastereoisomeric diphosphonates (1*R*,3*S*)-**6** and (1*R*,3*R*)-**6**, and (1*S*,3*R*)-**6** and (1*S*,3*S*)-**6**, we took advantage of the known stereochemical outcome of the cycloaddition of *N*-benzyl-*C*-(diethoxyphosphoryl)nitrone **13** with vinylphosphonate leading to the formation of a 76:12:12 mixture of the respective racemic isoxazolidines, *trans*-**14** (δ^31^P = 21.32 and 20.77 ppm), *cis*-**14** (δ^31^P = 20.81 and 19.49 ppm), and *trans*-**15** (δ^31^P = 27.42 and 21.15 ppm, both as doublets with *J* value 32.4 Hz), with *trans*-**14** predominating (Scheme 6) [16,17]. From this mixture, the major diastereoisomeric (isoxazolidine-3,5-diyl)-3,5-disphosphonate *trans*-**14** [(3*R*/*S*,5*R*/*S*)-**14**] and its 3,4-disubstituted regioisomer *trans*-**15** [(3*R*/*S*,5*S*/*R*)-**15**] were isolated on a silica gel column followed by HPLC with 17% and 3.5% yields, respectively. Compound *trans*-**14** was then efficiently transformed into *anti*-**6** via hydrogenolysis in the presence of Boc_2_O. The transformation of compound *trans*-**14** into **6** proceeded without changes in configuration of the stereogenic centres, thus the relative configuration between substituents at C1 and C3 in racemic diphosphonate *anti*-**6** [(1*R/S*,3*R/S*)-**6**] could be established unequivocally (Scheme 6), and therefore, the same applied to the *anti*-configured enantiomeric pair of diphosphonates (1*R*,3*R*)-**6** and (1*S*,3*S*)-**6** (Scheme 3 and Scheme 5). The addition of diethyl phosphite to aldehyde (*R*)-**7** or (*S*)-**7**, results in the formation of the corresponding *syn*-adduct **6** in addition to the isomeric *anti*-**6** product (stereochemical outcome of Abramov reaction). Consequently, the absolute configuration of the other pair of enantiomeric diphosphonates obtained from (*R*)-**7** and (*S*)-**7** were assigned as (1*R*,3*S*)-**6** and (1*S*,3*R*)-**6**, respectively (Scheme 3 and Scheme 5).

**Scheme 7:**
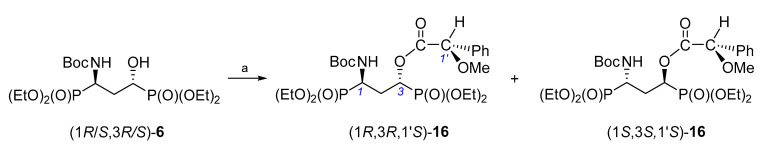
Synthesis of (*S*)-*O*-methylmandelate derivatives (1*R*,3*R*,1′*S*)-**16** and (1*S*,3*S*,1′*S*)-**16**. Reactions and conditions: (a) (*S*)-PhCH(OCH_3_)COOH, DCC, DMAP, CH_2_Cl_2_, rt, 24 h [14% for (1*R*,3*R*,1′*S*)-**16** and 10% for (1*S*,3*S*,1′*S*)-**16**].

To gather additional evidence of the absolute configurations at C3 in the respective 3-hydroxydiphosphonates **6**, the racemic compound *anti*-**6** [(1*R/S*,3*R/S*)-**6**] available from isoxazolidine *trans*-**14** [(3*R/S*,5*R/S*)-**14**] was transformed into a diastereoisomeric mixture of *O*-methylmandelate derivatives (1*R*,3*R*,1′*S*)-**16** (δ^31^P = 24.34 and 18.42 ppm) and (1*S*,3*S*,1′*S*)-**16** (δ^31^P = 23.98 and 19.32 ppm) via esterification with (*S*)-*O*-methylmandelic acid [18] in the presence of DCC (N,N′-dicyclohexylcarbodiimide) [19] (Scheme 7). Although separation of the diastereoisomeric *O*-methylmandelates was tedious with HPLC, mainly due to problems with removal of dicyclohexylurea (DCU), sufficient amounts of the diastereoisomers were obtained to collect their ^1^H and ^31^P NMR spectra (see Supplementary Materials), i.e., (1*R*,3*R*,1′*S*)-**16** eluted faster than (1*R*,3*S*,1′*S*)-**16** (Scheme 4). Moreover, the *O*-methylmandelates **16** appeared unstable, even at −4 °C.

**Figure 3:**
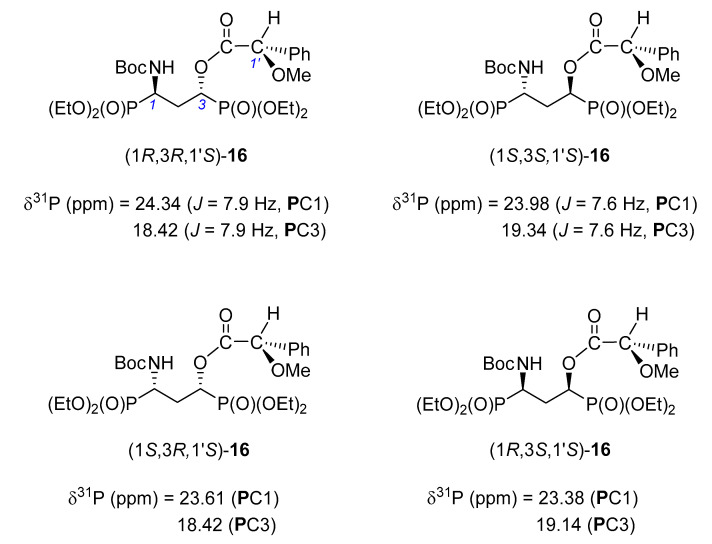
Structures of diastereoisomeric (*S*)-*O*-methylmandelates **16**.

To synthesize all diastereoisomeric (*S*)-*O*-methylmandelic acid esters of the 3-hydroxydiphosphonates **6**, analogous reactions were performed on the respective mixtures of diastereoisomeric phosphonates **6** obtained directly from enantiomerically pure aldehydes (Scheme 3 and Scheme 5). Thus, a mixture of the 3-hydroxydiphosphonates (1*R*,3*S*)-**6** and (1*R*,3*R*)-**6** obtained from aldehyde (*R*)-**7** was converted into (*S*)-*O*-methylmandelates (1*R*,3*S*,1′*S*)-**16** and (1*R*,3*R,*1′*S*)-**16**, whereas esters (1*S*,3*R*,1′*S*)-**16** and (1*S*,3*S,*1′*S*)-**16** were synthesized from the 3-hydroxydiphosphonates (1*S*,3*R*)-**6** and (1*S*,3*S*)-**6** produced from aldehyde (*S*)-**7** (Figure 3).

Based on extensive configurational studies of the α-hydroxyphosphonates, Spilling and co-workers concluded that ^31^P NMR chemical shifts for the (*R*)-*O*-methylmandelic acid esters of (*S*)-α-hydroxyphosphonates appear in a higher field compared to the signals for the (*R*)-*O*-methylmandelates of enantiomeric (*R*)-alcohols [20]. Accordingly, (*S*)-*O*-methylmandelates of (*R*)-α-hydroxyphosphonates are expected to absorb in a higher field than (*S*)-*O*-methylmandelates of (*S*)-α-hydroxyphosphonates. Indeed, this general rule worked well for our 3-hydroxydiphosphonates **6** (Figure 3). Thus, the ^31^P nucleus at C3 in (*S*)-*O*-methylmandalate (1*R*,3*R*,1′*S*)-**16** resonates in a higher field (δ^31^P = 18.42 ppm) compared to the diastereoisomeric ester (1*S*,3*S*,1′*S*)-**16** (δ^31^P = 19.34 ppm) obtained from the enantiomeric α-hydroxydiphosphonate (1*S*,3*S*)-**6**. Similarly, a lower value for the ^31^P NMR chemical shift of the phosphorus atom at C3 in (*S*)-*O*-methylmandelate (1*S*,3*R*,1′*S*)-**16** (δ^31^P = 18.42 ppm) was observed in comparison to the respective signal for (1*R*,3*S*,1′*S*)-**16** (δ^31^P = 19.14 ppm). Thereby, comparison of the ^31^P NMR chemical shifts for the respective pairs of (*S*)-*O*-methylmandelic acid esters of enantiomeric hydroxydiphosphonates, i.e., (1*R*,3*R*,1′*S*)-**16** and (1*S*,3*S*,1′*S*)-**16**, and (1*S*,3*R*,1′*S*)-**16** and (1*R*,3*S*,1′*S*)-**16**, provided unambiguous evidence for the already established absolute configurations of the isomeric 1-amino-3-hydroxydiphosphonates (1*R*,3*R*)-**6**, (1*S*,3*S*)-**6**, (1*S*,3*R*)-**6**, and (1*R*,3*S*)-**6**, respectively.

## 3. Materials and Methods

### 3.1. General Information

NMR spectra were measured in chloroform-*d* (CDCl_3_), benzene-*d6* (C_6_D_6_), or deuterium oxide (D_2_O) on a Bruker Avance III (600 MHz). Solvent signals or TMS were used as internal references for ^1^H and ^13^C chemical shifts (ppm). ^31^P signals were referenced through the solvent lock (2H) signal according to the IUPAC recommended secondary referencing method and the manufacturer’s protocols (an analogous protocol was used for ^13^C NMR spectra recorded in D_2_O). Coupling constants *J* are given in Hz. The NMR experiments were conducted at 300K with the following parameters: ^1^H NMR spectra were acquired at 600.26 MHz using 30°-pulses (zg30), a spectral width of 12,335.5 Hz, acquisition time 2.6564 s, collecting an average of 16 scans, a relaxation delay of 1.0 sec, a pulse width 9.4 µs; ^13^C NMR were acquired at 150.95 MHz with 30°-pulses (zgpg30), a spectral width of 36,057.7 Hz, acquisition time 0.9088 s, collecting an average of 8192 scans, a relaxation delay of 2.0 s, a pulse width 10.5 µs; ^31^P NMR were acquired at 242.98 MHz with 30°-pulses (zgpg30), a spectral width of 96,153.8 Hz, acquisition time 0.3408 s, collecting an average of 128 scans, a relaxation delay of 2.0 s, a pulse width 13.7 µs. IR spectroscopic data were measured on an Bruker Alpha-T FT-IR spectrometer. Melting points were determined with a Boetius apparatus and are uncorrected. Elemental analyses were performed by the Microanalytical Laboratory of the Faculty of Pharmacy (Medical University of Lodz) with a Perkin Elmer PE 2400 CHNS analyzer, and their results were found to be in good agreement (±0.3%) with the calculated values. Polarimetric measurements were conducted with an Optical Activity PolAAr 3001 apparatus. HPLC separations were performed using a Waters HPLC system consisting of binary HPLC pump (Waters 2545), a diode array detector (Waters 2998) and an auto sampler (Waters 2767), and an XBridge C18 column OBD, 19 × 100 mm with a particle size of 5μm. The following adsorbents were used: column chromatography, Merck silica gel 60 (70–230 mesh); analytical TLC, Merck TLC plastic sheets silica gel 60 F254. TLC plates were developed in chloroform–methanol and chloroform–isopropanol solvent systems. Visualization of spots was achieved with iodine vapours. All solvents were purified by methods described in the literature.

### 3.2. General Procedure for the Synthesis of (1R,3S)-***6*** and (1R,3R)-***6*** or (1S,3R)-***6*** and (1S,3S)-***6***

Crude aldehyde (*R*)-**7** or (*S*)-**7** (1.0 mmol) and diethyl phosphite (5.0 mmol) containing triethylamine (0.1 mmol) were left at room temperature for 48 h. The crude product was purified on a silica gel column with chloroform-methanol (100:1 *v*/*v*) to give an inseparable mixture of diphosphonates (1*R*,3*S*)-**6** and (1*R*,3*R*)-**6** or (1*S*,3*R*)-**6** and (1*S*,3*S*)-**6**.

Tetraethyl (1*R*,3*S*)- and (1*R*,3*R*)-[1-(*N*-Boc-amino)-3-hydroxypropane-1,3-diyl]diphosphonate [(1*R*,3*S*)-6 and (1*R*,3*R*)-6]. From aldehyde (*R*)-**7** (0.292 g, 0.897 mmol), an inseparable mixture of diphosphonates (1*R*,3*R*)**-6** and (1*R*,3*S*)**-6** (0.296 g, 76%) was obtained. ^31^P NMR (243 MHz, CDCl_3_): δ = 25.26 [(1*R*,3*S*)**-6**], 24.60 [d, *J* = 8.0 Hz, (1*R*,3*R*)**-6**], 24.03 [d, *J* = 8.0 Hz, (1*R*,3*R*)**-6**], 23.58 [(1*R*,3*S*)**-6**]. Anal. Calcd. for C_16_H_35_NO_9_P_2_×0.25 H_2_O: C, 42.53; H, 7.92; N, 3.10. Found: C, 42.33; H, 7.91; N, 3.02.

Tetraethyl (1*S*,3*R*)- and (1*S*,3*S*)-[1-(*N*-Boc-amino)-3-hydroxypropane-1,3-diyl]diphosphonate [(1*S*,3*R*)-6 and (1*S*,3*S*)-6]. From aldehyde (*S*)-**7** (0.308 g, 0.950 mmol), an inseparable mixture of diphosphonates (1*S*,3*S*)-**6** and (1*S*,3*R*)-**6** (0.298 g, 72%) was obtained. ^31^P NMR (243 MHz, CDCl_3_): δ = 25.26 [(1*R*,3*S*)**-6**], 24.60 [d, *J* = 8.0 Hz, (1*R*,3*R*)**-6**], 24.03 [d, *J* = 8.0 Hz, (1*R*,3*R*)**-6**], 23.58 [(1*R*,3*S*)**-6**]. Anal. Calcd. for C_16_H_35_NO_9_P_2_·0.25 H_2_O: C, 42.53; H, 7.92; N, 3.10. Found: C, 42.38; H, 8.11; N, 3.18.

### 3.3. General Procedure for the Synthesis of Tetraethyl [1-(N-Boc-amino)-3-Acetoxypropane-1,3-Diyl]Diphosphonate ***11***

A 1:1 mixture of diphosphonates (1*R*,3*R*)-**6** and (1*R*,3*S*)-**6** or (1*S*,3*S*)-**6** and (1*S*,3*R*)-**6**, acetic anhydride (1.5 mmol), triethylamine (2.0 mmol), and catalytic amounts of DMAP (1 crystal) in methylene chloride (1 mL) were stirred at room temperature for 4 h. The reaction mixture was washed with water (3 × 5 mL), dried over MgSO_4_, concentrated in vacuo and chromatographed on a silica gel column with chloroform-isopropanol (100:1 *v*/*v*). Diastereoisomers were separated by HPLC with a mobile phase of water-acetonitrile (70:30, *v/v*) at a flow rate of 17 mL/min to yield (1*R*,3*R*)-**11** and (1*R*,3*S*)-**11** or (1*S*,3*S*)-**11** and (1*S*,3*R*)-**11**.

#### 3.3.1. Synthesis of (1*R*,3*S*)-**11** and (1*R*,3*R*)-**11**

From a 1:1 mixture of 3-hydroxydiphosphonates (1*R*,3*S*)-**6** and (1*R*,3*R*)-**6** (0.149 g, 0.345 mmol), compound (1*R*,3*S*)-**11** (0.037 g, 22%) was obtained followed by (1*R*,3*R*)**-11** (0.067 g, 40%).

Tetraethyl (1*R*,3*S*)-[1-(*N*-Boc-amino)-3-acetoxypropane-1,3-diyl]diphosphonate [(1*R*,3*S*)-11]. Colourless oil; *t_R_* = 10.69 min. ${\left[\alpha \right]}_{D}^{20}$ = +3.27 (*c* 1.04, CHCl_3_). IR (film): ν = 3483, 3249, 2982, 2934, 2872, 1752, 1708, 1532, 1296, 1222, 1024, 969 cm^−1^. ^1^H NMR (600 MHz, CDCl_3_): δ = 5.38 (ddd, 1H, *J* = 4.7 Hz, *J* = 8.9 Hz, *J* = 13.7 Hz, *H*C3), 4.92 (d, 1H ^3^*J* = 10.3 Hz, N*H*), 4.22–4.05 (m, 9H, 4 × C*H*_2_OP and *H*C1), 2.47–2.39 (m, 1H, *H_a_*CH_b_), 2.10 (s, 3H, C*H*_3_), 2.09–1.99 (m, 1H, *H_a_*C2), 1.41 (s, 9H, 3 × C*H*_3_), 1.34–1.26 (m, 4 × C*H*_3_CH_2_OP). ^13^C NMR (151 MHz, CDCl_3_): δ = 169.85 (d, ^3^*J*_COCP_ = 5.1 Hz), 154.97 (d, ^3^*J*_CNCP_ = 5.4 Hz), 80.33, 65.60 (dd, ^1^*J*_CP_ = 168.7 Hz, ^3^*J*_CCCP_ = 12.2 Hz), 63.20 (d, ^2^*J*_COP_ = 7.1 Hz), 63.15 (^2^*J*_COP_ = 7.0 Hz), 63.11 (^2^*J*_COP_ = 6.4 Hz), 62.72 (^2^*J*_COP_ = 6.7 Hz), 53.56, 44.88 (dd, ^1^*J*_CP_ = 158.0 Hz, ^3^*J*_CCCP_ = 12.4 Hz), 30.49 (dd, ^2^*J*_CCP_ = 4.8 Hz, ^2^*J*_CCP_ = 2.0 Hz), 28.39 (3 × *C*H_3_), 21.01, 16.59 (d, ^3^*J*_CCOP_ = 4.0 Hz), 16.56 (d, ^3^*J*_CCOP_ = 3.8 Hz), 16.38 (d, ^3^*J*_CCOP_ = 6.0 Hz), 16.46 (d, ^3^*J*_CCOP_ = 6.6 Hz). ^31^P NMR (243 MHz, CDCl_3_): δ = 23.55 (d, ^4^*J*_PCCCP_ = 4.2 Hz), 19.83 (d, ^4^*J*_PCCCP_ = 4.2 Hz). Anal. Calcd. for C_18_H_37_NO_10_P_2_: C, 44.18; H, 7.62; N, 2.86. Found: C, 44.01; H, 7.82; N, 2.90.

Tetraethyl (1*R*,3*R*)-[1-(*N*-Boc-amino)-3-acetoxypropane-1,3-diyl]diphosphonate [(1*R*,3*R*)-11]. White amorphous solid; *t_R_* = 12.67 min. ${\left[\alpha \right]}_{D}^{20}$ = −21.80 (*c* 1.22, CHCl_3_). Mp = 84–88 °C. IR (KBr): ν = 3480, 3262, 2983, 2935, 1710, 1674, 1251, 1225, 1024, 978 cm^−1^_._^1^H NMR (600 MHz, CDCl_3_): δ = 5.39 (ddd, 1H, *J* = 2.0 Hz, *J* = 8.5 Hz, *J* = 11.5 Hz, *H*C3), 4.67 (d, 1H ^3^*J* = 10.8 Hz, N*H*), 4.39–3.96 (m, 9H, 4 × C*H*_2_OP, *H*C1), 2.58–2.32 (m, 1H, *H_a_*C2), 2.14 (s, 3H, C*H*_3_), 2.14–2.07 (m, 1H, *H_b_*C2), 1.44 (s, 9H, 3 × C*H*_3_), 1.36–1.31 (m, 4 × C*H*_3_CH_2_OP).^13^C NMR (151 MHz, CDCl_3_): δ = 169.52 (d, ^3^*J*_COCP_ = 2.2 Hz), 155.07 (d, ^3^*J*_CNCP_ = 3.9 Hz), 80.47, 63.13 (d, ^2^*J*_COP_ = 6.1 Hz), 62.97 (d, ^2^*J*_COP_ = 7.2 Hz), 62.89 (d, ^2^*J*_COP_ = 7.1 Hz), 62.78 (d, ^2^*J*_COP_ = 6.5 Hz), 62.66 (dd, ^1^*J*_CP_ = 167.3 Hz, ^3^*J*_CCCP_ = 13.6 Hz), 42.53 (dd, ^1^*J*_CP_ = 158.8 Hz, ^3^*J*_CCCP_ = 14.3 Hz), 28.95 (dd, ^2^*J*_CCP_ = 3.9 Hz, ^2^*J*_CCP_ = 7.5 Hz), 28.30 (3 × *C*H_3_), 20.74, 16.55 (d, ^3^*J*_CCOP_ = 5.7 Hz, 2 × *C*H_3_), 16.46 (d, ^3^*J*_CCOP_ = 5.9 Hz), 16.45 (d, ^3^*J*_CCOP_ = 5.6 Hz). ^31^P NMR (243 MHz, CDCl_3_): δ = 24.41 (d, ^4^*J*_PCCCP_ = 7.9 Hz), 20.53 (d, ^4^*J*_PCCCP_ = 7.9 Hz). Anal. Calcd. for C_18_H_37_NO_10_P_2_: C, 44.18; H, 7.62; N, 2.86. Found: C, 44.12; H, 7.95; N, 2.91.

#### 3.3.2. Synthesis of (1*S*,3*R*)-**11** and (1*S*,3*S*)-**11**

From a 1:1 mixture of 3-hydroxydiphosphonates (1*S*,3*R*)-**6** and (1*S*,3*S*)-**6** (0.098 g, 0.227 mmol), compound (1*S*,3*R*)-**11** (0.022 g, 20%) was obtained followed by (1*R*,3*R*)**-11** (0.036 g, 32%).

Tetraethyl (1*S*,3*R*)-[1-(*N*-Boc-amino)-3-acetoxypropane-1,3-diyl]diphosphonate [(1*S*,3*R*)-11] [enantiomer of (1*R*,3*S*)-11]. Colourless oil; *t_R_* = 10.69 min.$\;{\left[\alpha \right]}_{D}^{20}$ = −3.45 (*c* 1.10, CHCl_3_). Anal. Calcd. for C_18_H_37_NO_10_P_2_ × 0.25 H_2_O: C, 44.18; H, 7.62; N, 2.86. Found: C, 44.00; H, 7.88; N, 2.96.

Tetraethyl (1*S*,3*S*)-[1-(*N*-Boc-amino)-3-acetoxypropane-1,3-diyl]diphosphonate [(1*S*,3*S*)-11] [enantiomer of (1*R*,3*R*)-11]. White amorphous solid; *t_R_* = 12.67 min. ${\left[\alpha \right]}_{D}^{20}$ = +20.30 (*c* 1.01, CHCl_3_). Anal. Calcd. for C_18_H_37_NO_10_P_2_: C, 44.18; H, 7.62; N, 2.86. Found: C, 44.11; H, 7.85; N, 2.97.

### 3.4. General Procedure for the Synthesis of Tetraethyl [1-(N-Boc-amino)-3-(4-Nitrobenzoyloxy)Propane-1,3-Diyl]Diphosphonate ***12***

A 1:1 mixture of diphosphonates (1*R*,3*R*)-**6** and (1*R*,3*S*)-**6** or (1*S*,3*S*)-**6** and (1*S*,3*R*)-**6**, 4-nitrobenzoyl chloride (1.5 mmol), and triethylamine (2.0 mmol) containing DMAP (1 crystal) in methylene chloride (1 mL) was stirred at room temperature for 4 h. The reaction mixture was washed with water (3 × 5 mL), dried over Na_2_SO_4_, concentrated in vacuo and chromatographed on a silica gel column with dichloromethane-isopropanol (100:1 *v*/*v*). Diastereoisomers were separated by HPLC with a mobile phase of water-acetonitrile (64:38, *v/v*) at a flow rate of 17 mL/min to yield (1*R*,3*R*)-**12** and (1*R*,3*S*)-**12** or (1*S*,3*S*)-**12** and (1*S*,3*R*)-**12**.

#### 3.4.1. Synthesis of (1*R*,3*S*)-**12** and (1*R*,3*R*)-**12**

From a 1:1 mixture of 3-hydroxydiphosphonates (1*R*,3*R*)-**6** and (1*R*,3*S*)-**6** (0.099 g, 0.229 mmol), compound (1*R*,3*S*)-**12** (0.029 g, 21%) was obtained followed by (1*R*,3*R*)-**12** (0.043 g, 31%).

Tetraethyl (1*R*,3*S*)-[1-(*N*-Boc-amino)-3-(4-nitrobenzoyloxy)propane-1,3-diyl]diphosphonate [(1*R*,3*S*)-12]. Yellowish oil; *t_R_* = 14.89 min.$\;{\left[\alpha \right]}_{D}^{20}$ = −4.60 (c 2.65, CHCl_3_). IR (film): ν = 3290, 3050, 2982, 2932, 1739, 1704, 1530, 1394, 1367, 1243, 1053, 1024, 716 cm^−1^. ^1^H NMR (600 MHz, CDCl_3_): δ = 8.33 (d, 2H, ^3^*J* = 8.8 Hz), 8.28 (d, 2H, ^3^*J* = 8.6 Hz), 5.72 (ddd, 1H, *J* = 4.4 Hz, *J* = 9.0 Hz, ^2^*J*_PC1H_ = 9.1 Hz, *H*C3), 4.98 (d, 1H. ^3^*J* = 10.2 Hz, N*H*), 4.32–4.26 (m, 1H, *H*C1), 4.25–4.09 (m, 8H, 4 × C*H*_2_OP), 2.66–2.58 (m, 1H, *H_a_*C2), 2.35–2.26 (m, 1H, *H_b_*C2), 1.36 (s, 9H, 3 × C*H*_3_), 1.35–1.32 (m, 12H, 4 × C*H*_3_CH_2_OP). ^13^C NMR (151 MHz, CDCl_3_): δ = 163.66 (d, ^3^*J*_COCP_ = 4.3 Hz), 155.01 (d, ^3^*J*_CNCP_ = 5.5 Hz), 150.90, 135.04, 131.21, 123.76, 80.40, 67.33 (dd, ^1^*J*_CP_ = 11.7 Hz, ^3^*J*_CCCP_ = 168.2 Hz), 63.36 (d, ^2^*J*_COP_ = 6.5 Hz), 63.31 (d, ^2^*J*_COP_ = 6.1 Hz), 63.25 (d, ^2^*J*_COP_ = 7.1 Hz), 62.83 (d, ^2^*J*_COP_ = 6.8 Hz), 44.99 (dd, ^1^*J*_CP_ = 157.7 Hz, ^3^*J* _CCCP_ = 12.4 Hz), 30.74 (d, ^2^*J*_CCP_ = 3.0 Hz), 28.28 (3 × *C*H_3_), 16.63 (d, ^3^*J*_CCOP_ = 5.5 Hz), 16.54 (d, ^3^*J*_CCOP_ = 5.7 Hz), 16.46 (d, ^3^*J*_CCOP_ = 5.7 Hz). ^31^P NMR (243 MHz, CDCl_3_): δ = 23.33 (d, ^4^*J*_PCCCP_ = 4.1 Hz) and 18.97 (d, ^4^*J*_PCCCP_ = 4.1 Hz). Anal. Calcd. for C_23_H_38_N_2_O_12_P_2_: C, 46.32; H, 6.42; N, 4.70. Found: C, 46.13; H, 6.32, N, 4.71.

Tetraethyl (1*R*,3*R*)-[1-(*N*-Boc-amino)-3-(4-nitrobenzoyloxy)propane-1,3-diyl]diphosphonate [(1*R*,3*R*)-12]. White amorphous solid; *t_R_* = 18.14 min. ${\left[\alpha \right]}_{D}^{20}$ = −41.34 (*c* 0.82, CHCl_3_). Mp = 125–126 °C. IR (KBr): ν = 3288, 3049, 2982, 2930, 1740, 1704, 1530, 1368, 1243, 1053, 1024, 716 cm^−1^. ^1^H NMR (600 MHz, CDCl_3_): δ = 8.32 (d, 2H, ^3^*J* = 8.8 Hz), 8.22 (d, 2H, ^3^*J* = 8.8 Hz), 5.69 (ddd, 1H, *J* = 1.84 Hz, *J* = 8.3 Hz, *J* = 12.5 Hz, *H*C3), 4.74 (d, 1H, *J* = 10.5 Hz, N*H*), 4.25–4.09 (m, 9H, 4 × C*H*_2_OP and *H*C1), 2.67–2.61 (m, 1H, *H_a_*C2), 2.27–2.21 (m, 1H, *H_b_*C2), 1.40 (s, 9H, 3 × C*H*_3_), 1.35–1.30 (m, 4 × C*H*_3_CH_2_OP). ^13^C NMR (151 MHz, CDCl_3_): δ = 163.34 (d, ^3^*J*_COCP_ = 2.5 Hz), 155.00 (d, ^3^*J*_CNCP_ = 4.3 Hz), 150.80, 135.16, 131.00, 123.76, 80.57, 64.38 (dd, ^1^*J*_CP_ = 167.7 Hz, ^3^*J*_CCCP_ = 13.5 Hz), 63.39 (d, ^2^*J*_COP_ = 6.3 Hz), 63.17 (d, ^2^*J*_COP_ = 7.2 Hz), 62.98 (d, ^2^*J*_COP_ = 6.6 Hz), 62.94 (d, ^2^*J*_COP_ = 6.1 Hz), 42.73 (dd, ^1^*J*_CP_ = 158.5 Hz, ^3^*J*_CCCP_ = 14.3 Hz), 29.32 (^2^*J*_CCP_ = 3.4 Hz, ^2^*J*_CCP_ = 7.3 Hz), 28.30 (3 × *C*H_3_), 16.62 (d, ^3^*J*_CCOP_ = 5.6 Hz), 16.56 (d, ^3^*J*_CCOP_ = 5.8 Hz), 16.50 (d, ^3^*J*_CCOP_ = 5.8 Hz), 16.46 (d, ^3^*J*_CCOP_ = 5.9 Hz). ^31^P NMR (243 MHz, CDCl_3_): δ = 24.10 (d, *J*_PCCCP_ = 7.5 Hz) and 19.69 (d, *J*_PCCCP_ = 7.5 Hz). Anal. Calcd. for C_23_H_38_N_2_O_12_P_2_: C, 46.31; H, 6.42; N, 4.70. Found: C, 46.29; H, 6.43, N, 4.59.

#### 3.4.2. Synthesis of (1*S*,3*R*)-**12** and (1*S*,3*S*)-**12**

From a 1:1 mixture of 3-hydroxydiphosphonates (1*S*,3*S*)-**6** and (1*S*,3*R*)-**6** (0.094 g, 0.218 mmol), compound (1*S*,3*R*)-**12** (0.022 g, 17%) was obtained followed by (1*S*,3*S*)-**12** (0.018g, 14%).

Tetraethyl (1*S*,3*R*)-[1-(*N*-Boc-amino)-3-(4-nitrobenzoyloxy)propane-1,3-diyl]diphosphonate [(1*S*,3*R*)-12] [enantiomer of (1*R*,3*S*)-12]. Colourless oil; *t_R_* = 14.89 min. ${\left[\alpha \right]}_{D}^{20}$ = +2.78 (*c* 2.16, CHCl_3_). Anal. Calcd. for C_23_H_38_N_2_O_12_P_2_: C, 46.31; H, 6.42; N, 4.70. Found: C, 46.18; H, 6.49; N, 4.73.

Tetraethyl (1*S*,3*S*)-[1-(*N*-Boc-amino)-3-(4-nitrobenzoyloxy)propane-1,3-diyl]diphosphonate [(1*S*,3*S*)-12] [enantiomer of (1*R*,3*R*)-12]. White amorphous solid; *t_R_* = 18.14 min. Mp = 116–118 °C. ${\left[\alpha \right]}_{D}^{20}$ = +40.60 (*c* 0.83, CHCl_3_). Anal. Calcd. for C_23_H_38_N_2_O_12_P_2_: C, 46.31; H, 6.42; N, 4.70. Found: C, 46.60; H, 6.64; N, 4,71.

### 3.5. General Procedure for the Hydrolysis of ***11*** or ***12***

A solution of the respective enantiomers of compound **11** or **12** (1.0 mmol) in 5M HCl (15 mL) was refluxed for 6 h. The solvent was removed under reduced pressure, and the residue was suspended in mixture of methanol-water (15 mL) and neutralized with propylene oxide and concentrated in vacuo. The reside was dissolved in 10 mL deionised water. Compounds (1*S*,3*S*)-**5** and (1*R*,3*R*)-**5** were precipitated by adding isopropanol; compounds (1*S*,3*R*)-**5** and (1*S*,3*R*)-**5** were precipitated by adding methanol.

(1*R*,3*S*)-(1-amino-3-hydroxypropane-1,3-diyl)diphosphonic acid [(1*R*,3*S*)-5]. From compound (1*R*,3*S*)-**11** (0.051 g, 0.104 mmol), diphosphonic acid (1*S*,3*R*)-**5** (0.019 g, 0.081 mmol, 53%) was obtained. White amorphous solid. Mp > 290 °C. ${\left[\alpha \right]}_{D}^{20}$ = +6.55 (*c* 0.61, 5% NH_3_). IR (KBr): ν = 3390, 3241, 2960, 2932, 1651, 1519, 1454, 1167, 1081, 919, 809, 723 cm^−1^. ^1^H NMR (600 MHz, D_2_O): δ = 3.94 (ddd, 1H, *J* = 3.4 Hz, *J* = 7.3 Hz, *J* = 10.6 Hz, C*H*P), 3.41(ddd, 1H, *J* = 4.4 Hz, *J* = 9.6 Hz, *J* = 13.7 Hz, C*H*P), 2.28–2.22 (m, 1H), 1.96–1.87 (m, 1H). ^13^*C* NMR (151 MHz, D_2_O): δ = 67.91 (dd, ^1^*J*_PC_ = 156.1 Hz, ^3^*J*_PCCC_ = 10.2 Hz), 48.35 (dd, ^1^*J*_PC_ = 141.4 Hz, ^3^*J*_PCCC_ = 13.4 Hz), 30.02. ^31^P NMR (243 MHz, D_2_O): δ = 17.88 and 12.41. C_3_H_11_NO_11_P_2_·0.25 H_2_O: C, 15.04; H, 4.84; N, 5.85. Found: C, 15.07; H, 4.88; N, 5.89.

(1*R*,3*R*)-(1-amino-3-hydroxypropane-1,3-diyl)diphosphonic acid [(1*R*,3*R*)-5]. From compound (1*R*,3*R*)-**11** (0.043 g, 0.088 mmol), diphosphonic acid (1*R*,3*R*)-**5** (0.017 g, 84%) was obtained. White amorphous solid. Mp > 290 °C. IR (KBr): ν= 3406, 3252, 2960, 2926, 2855, 1636, 1532, 1438, 1165, 1062, 912, 717 cm^−1^. ${\left[\alpha \right]}_{D}^{20}$ = −9.71 (*c* 0.68, 5% NH_3_). ^1^H NMR (600 MHz, D_2_O): δ = 3.81 (ddd, 1H, *J* = 3.9 Hz, *J* = 9.2 Hz, *J* = 13.1 Hz, C*H*P), 3.43 (ddd, 1H, *J* = 3.2 Hz, *J* = 10.3 Hz, *J* = 13.6 Hz, C*H*P), 2.18–2.10 (m, 1H), 2.08–2.00 (m, 1H). ^13^C NMR (151 MHz, D_2_O): δ = 65.45 (dd, ^1^*J*_PC_ = 157.0 Hz, ^3^*J*_PCCC_ = 11.3 Hz), 46.55 (dd, ^1^*J*_PC_ = 137.3 Hz, ^3^*J*_PCCC_ = 12.2 Hz), 30.02. ^31^P NMR (243 MHz, D_2_O): δ = 18.97 and 12.89. Anal. Calcd. for C_3_H_11_NO_11_P_2_·0.25 H_2_O: C, 15.04; H, 4.84; N, 5.85. Found: C, 15.12; H, 4.87; N, 5.84.

(1*S*,3*R*)-(1-amino-3-hydroxypropane-1,3-diyl)diphosphonic acid [(1*S*,3*R*)-5] [enantiomer of (1*R*,3*S*)-5]. From compound (1*S*,3*R*)-**11** (0.054 g, 0.11 mmol), diphosphonic acid (1*R*,3*R*)-**5** (0.022 g, 86%) was obtained as a white amorphous solid. Mp > 290 °C. ${\left[\alpha \right]}_{D}^{20}$ = −4.64 (*c* 0.56, 5% NH_3_). Anal. Calcd. for C_3_H_11_NO_11_P_2_·0.25 H_2_O: C, 15.04; H, 4.84; N, 5.85. Found: C, 15.19; H, 4.89; N, 5.87.

(1*S*,3*S*)-(1-amino-3-hydroxypropane-1,3-diyl)diphosphonic acid [(1*S*,3*S*)-5] [enantiomer of (1*R*,3*R*)-5]. From compound (1*S*,3*S*)-**11** (0.052 g, 0.106 mmol), diphosphonic acid (1*S*,3*S*)-**5** (0.017 g, 69%) was obtained as a white amorphous solid. Mp > 290 °C. ${\left[\alpha \right]}_{D}^{20}$ = +8.62 (*c* 0.83, 5% NH_3_). Anal. Calcd. for C_3_H_11_NO_11_P_2_·0.25 H_2_O: C, 15.04; H, 4.84; N, 5.85. Found: C, 15.21; H, 4.85; N, 5.86.

### 3.6. Cycloaddition of Nitrone ***13*** to Vinylphosphonate

Nitrone **13** (0.710 g, 2.617 mmol) and vinylphosphonate (0.389 mL, 2.617 mmol) were stirred in toluene (3.0 mL) at 60 °C for 48 h. All volatiles were removed in vacuo and the crude products were subjected to purification on a silica gel column with chloroform-isopropanol (100:1, *v*/*v* then 50:1 *v*/*v*) to yield (3*R/S*,5*R/S*)-**14** [*anti*-**14**] (0.331 g, 17%). The residue was separated by HPLC with a mobile phase of water-isopropanol (80:20, *v/v*) at a flow rate of 17 mL/min to yield (3*R/S*,4*S/R*)-**15** [*anti*-**15**] (0.040 g, 3.5%).

Tetraethyl (3*R/S*,5*R/S*)-(2-benzylisoxazolidine-3,5-diyl)diphosphonate [(3*R/S*,5*R/S*)-14]. Colourless oil. IR (film): ν = 3477, 2984, 2931, 2911, 1650, 1246, 1048, 1025, 970 cm^−1^. ^1^H NMR (CDCl_3_, 600 MHz): δ = 7.41 (d, *J* = 7.3 Hz, 2H), 7.32 (t, *J* = 7.4 Hz, 2H), 7.28 (d, *J* = 7.8 Hz, 1H), 4.36 (d, 1H, *J*_AB_ = 13.8 Hz, *H*_a_CH_b_Ph), 4.29 (dt, 1H, *J* = 8.3 Hz, *J* = 2.1 Hz, *H*C5), 4.25–4.08 (m, 4 × C*H*_2_OP and H_a_C*H_b_*Ph), 3.46 (ddd, 1H, *J* = 4.9 Hz, *J* = 6.4 Hz, *J* = 8.3 Hz, *H*C3), 2.91–2.80 (m, 2H, *H_a_*C4 and *H_b_*C4), 1.38–1.31 (m, 9H, 3 × C*H*_3_CH_2_OP), 1.29 (t, 3H, *J* = 7.1 Hz, C*H*_3_CH_2_OP). ^13^C NMR (CDCl_3_, 151 MHz): δ = 137.00, 129.49, 128.31, 127.51, 72.56 (dd, ^1^*J*_PC_ = 167.7 Hz, ^3^*J*_PCCC_ = 6.1 Hz, C5), 63.42 (d, ^2^*J*_COP_ = 6.5 Hz), 63.28 (d, ^2^*J*_COP_ = 6.5 Hz), 63.05 (d, *J* = 8.0 Hz), 62.92 (d, ^2^*J*_COP_ = 6.9 Hz), 62.60 (d, ^2^*J*_COP_ = 6.9 Hz), 61.23 (dd, ^1^*J*_PC_ = 170.8 Hz, ^3^*J*_PCCC_ = 5.8 Hz, C3), 33.45, 16.62 (d, ^3^*J*_CCOP_ = 6.5 Hz), 16.53 (d, ^3^*J*_CCOP_ = 6.3 Hz). ^31^P NMR (243 MHz, CDCl_3_): δ = 21.32 and 20.77. Anal. Calcd. for C_18_H_31_NO_7_P_2_·0.25 H_2_O: C, 49.15; H, 7.22; N, 3.19. Found: C, 49.06; H, 7.07; N, 3.27.

Tetraethyl (3*R/S*,4*S/R*)-(2-benzylisoxazolidine-3,4-diyl)diphosphonate [(3*R/S*,4*S/R*)-15]. Colourless oil; *t_R_* = 14.13 min. ^1^H NMR (600 MHz, CDCl_3_): δ = 7.43 (d, *J* = 6.9 Hz, 1H), 7.33 (t, *J* = 7.4 Hz, 1H), 7.28 (d, *J* = 7.4 Hz, 1H), 4.28 (ddd, ^3^*J* = 15.1 Hz, ^3^*J* = 8.9 Hz, *^3^J* = 7.0 Hz, 1H, *H_a_*C5), 4.23 (d, *J*_AB_ = 12.9 Hz, 1H), 4.21–4.14 (m, 8H, 4 × C*H*_2_OP), 4.08 (d, *J*_AB_ = 12.9 Hz, 1H), 4.08–4.03 (m, 1H, *H_b_*C5), 3.59 (ddd, ^2^*J* = 21.0 Hz, ^3^*J* = 7.2 Hz, ^3^*J* = 5.5 Hz, 1H, *H*C3), 3.26–3.11 (m, 1H, *H*C4), 1.36 (t, ^3^*J* = 7.1 Hz, 6H, 2 × C*H*_3_CH_2_OP), 1.30 (t, ^3^*J* = 7.1 Hz, 3H, C*H*_3_CH_2_OP), 1.27 (t, ^3^*J* = 7.1 Hz, 3H, C*H*_3_CH_2_OP). ^13^C NMR (151 MHz, CDCl_3_): δ = 136.54, 129.46, 128.35, 127.60, 66.57, 63.24 (d, ^2^*J*_COP_ = 7.0 Hz), 63.15 (d, ^2^*J*_COP_ = 6.6 Hz), 62.72 (d, ^2^*J*_COP_ = 6.6 Hz), 62.45 (d, ^2^*J*_COP_ = 6.6 Hz), 61.37 (d, *J* = 177.1 Hz), 41.95 (d, *J* = 147.1 Hz), 16.64 (d, ^3^*J*_CCOP_ = 3.6 Hz), 16.57 (d, ^3^*J*_CCOP_ = 5.2 Hz). ^31^P NMR (243 MHz, CDCl_3_): δ = 27.42 (d, *J*_PCCP_ = 32.4 Hz), 21.15 (d, *J*_PCCP_ = 32.4 Hz). Anal. Calcd. C_18_H_31_NO_7_P_2_: C, 49.66 H, 7.18; N, 3.22. Found: C, 49.55 H, 7.02; N, 3.12.

### 3.7. Synthesis of (1R/S,3R/S)-***6*** [Anti-***6***] from (3R/S,5R/S)-***6*** [Anti-***14***]

A solution of isoxazolidine (3*R/S*,5*R/S*)-**14** [*anti*-**14**] (0.046 g, 0.020 mmol) and Boc_2_O (0.023 g, 0.020 mmol) was kept under atmospheric pressure of hydrogen over 20% PdOH-C (5 mg) at room temperature for 2 days. The suspension was filtered through a layer of celite. The solution was concentrated, and the residue was chromatographed on a silica gel column with chloroform–isopropanol (100:1, *v*/*v*) to yield (1*R*/*S*,3*R*/*S*)-***6*** [*anti*-**6**] (0.032 g, 74%) as a colourless oil.

(1*R/S*,3*R/S*)-[1-(*N*-Boc-amino)-3-hydroxypropane-1,3-diyl]diphosphonate [(1*R/S*,3*R/S*)-6]. Colourless oil. IR (film): ν = 3417, 3281, 2982, 2931, 1698, 1393, 1368. 1232, 1166, 1046, 1026 cm^−1^. ^1^H NMR (CDCl_3_, 600 MHz): δ = 5.00 (dd, 1H, *J* = 10.8 Hz, *J* = 4.6 Hz, N*H*), 4.30–4.15 (m, 9H, 4 × C*H*_2_OP), 4.10 (d, 1H, *J* = 21.7 Hz, O*H*), 3.98 (dd, 1H, *J* = 11.7 Hz, *J* = 11.3 Hz, *H*CO), 2.23–2.15 (m, 1H, *H_a_*C2), 2.01–1.98 (m, 1H, *H_b_*C2), 1.47 (s, 9H, 3 × C*H*_3_), 1.38–1.34 (m, 12H, 4 × C*H*_3_CH_2_OP). ^13^C NMR (151 MHz, CDCl_3_): δ = 156.79 (d, ^3^*J*_CNCP_ = 9.0 Hz), 81.04, 63.80 (dd, ^1^*J*_PC_ = 170.6 Hz, ^3^*J*_PCCC_ = 13.2 Hz), 63.02 (d, ^2^*J*_COP_ = 7.0 Hz), 62.79 (d, ^2^*J*_COP_ = 2.2 Hz), 62.73 (d, ^2^*J*_COP_ = 6.8 Hz), 43.66 (dd, ^1^*J*_PC_ = 158.0 Hz, ^3^*J*_PCCC_ = 16.1 Hz), 32.72 (dd, ^2^*J*_PCC_ = 4.1 Hz, ^2^*J*_PCC_ = 4.0 Hz), 28.20, 16.50 (d,^3^*J*_CCOP_ = 2.9 Hz), 16.46 (d, ^3^*J*_CCOP_ = 2.9 Hz), 16.41 (d, ^3^*J*_CCOP_ = 5.7 Hz), 16.34 (d, ^3^*J*_CCOP_ = 5.7 Hz).^31^P NMR (CDCl_3_, 243 MHz): δ = 24.59 (d, ^4^*J*_PCCCP_ = 8.0 Hz), 24.93 (d, ^4^*J*_PCCCP_ = 8.0 Hz). ^1^H NMR (600 MHz, C_6_D_6_): δ = 5.86 (d, ^3^*J* = 10.0 Hz, 1H, N*H*), 5.36 (s, 1H, O*H*), 4.75 (ddt, *J* = 17.8 Hz, ^3^*J* = 10.0 Hz, *J* = 3.2 Hz, 1H, C*H*N), 4.41 (t, *J* = 10.1 Hz, 1H, C*H*O), 4.19–4.04 (m, 4H, 2 × C*H*_2_OP), 4.02–3.87 (m, 4H, 2 × C*H*_2_OP), 2.64–2.53 (m, 1H, *H_α_*CH_β_), 2.42–2.33 (m, 1H, *H_β_*CH_α_), 1.37 (s, 9H, 3 × C*H*_3_), 1.11 (t, ^3^*J* = 7.1Hz, 3H, C*H*_3_CH_2_OP), 1.10 (t, ^3^*J* = 7.1 Hz, 3H, C*H*_3_CH_2_OP), 1.04 (t, ^3^*J* = 7.1 Hz, 3H, C*H*_3_CH_2_OP), 1.03 (t, ^3^*J* = 7.1 Hz, 3H, C*H*_3_CH_2_OP). ^13^C NMR (151 MHz, C_6_D_6_): δ = 169.04, 156.28 (d, ^3^*J*_CNCP_= 5.8 Hz), 79.37, 64.45 (dd, ^1^*J*_PC_ = 155.1 Hz, ^3^*J*_PCCC_ =13.9 Hz), 62.44 (d, ^2^*J*_COP_ = 6.8 Hz), 62.25 (d, ^2^*J*_COP_ = 6.5 Hz), 62.15 (d, ^2^*J*_COP_ = 6.3 Hz), 44.26 (dd, ^1^*J*_PC_ = 156.1, ^3^*J*_PCCC_ = 15.7 Hz), 32.48, 27.96, 16.22 (d, ^3^*J*_CCOP_ = 5.3 Hz), 16.08 (d, ^3^*J*_CCOP_ = 5.2 Hz), 15.99 (d, ^3^*J*_CCOP_ = 5.7 Hz). ^31^P NMR (243 MHz, C_6_D_6_): δ = 25.33 (d, *J*_PCCCP_ = 7.1 Hz), 24.56 (d, *J*_PCCCP_ = 7.1 Hz). Anal. Calcd. for C_16_H_35_NO_9_P_2_ × 0.25 H_2_O: C, 42.53; H, 7.92; N, 3.10. Found: C, 42.38; H, 8.11; N, 3.09.

### 3.8. General Procedure for Esterification of 3-Hydroxydiphosphonates ***6*** with (S)-O-Methylmandelic Acid

To a solution of diphosphonate (1*R*/*S*,3*R/S*)-**6** or an appropriate mixture of diphosphonates (1*R*,3*S*)-**6** and (1*R*,3*R*)-**6** or (1*S*,3*R*)-**6** and (1*S*,3*S*)-**6** (1.00 mmol) in methylene chloride (3.5 mL), (*S*)-2-methoxy-2-phenylacetic acid (1.75 mmol), DCC (1.75 mmol) and DMAP (0.10 mmol) were added. This mixture was stirred at room temperature for 24 h. The reaction mixture was filtered off and concentrated in vacuo and chromatographed on a silica gel column with chloroform-isopropanol (100:1 *v*/*v*).

#### 3.8.1. Esterification of (1*R*/*S*,3*R*/*S*)-**6** with (*S*)-*O*-Methylmandelic Acid

From 3-hydroxydiphosphonate (1*R*/*S*,3*R/S*)-**6** (0.134 g, 0.585 mmol), (*S*)-*O*-methylmandelate (1*R*,3*R*,1′*S*)-**16** (0.026 g, 14%) was obtained followed by diastereoisomer (1*S*,3*S*,1′*S*)-**16** (0.018 g, 10%) after separation by HPLC with a mobile phase of water-acetonitrile (63:37, *v/v*) and a flow rate of 17 mL/min.

Mandelate (1*R*,3*R*,1′*S*)-**16**: white amorphous solid; *t_R_* = 15.77 min. ^1^H NMR (600 MHz, C_6_D_6_): δ = 7.52 (d, 2H, ^3^*J* = 7.3 Hz), 7.08 (t, 2H, ^3^*J* = 7.6 Hz), 7.00 (t, 1H, ^3^*J* = 7.6 Hz), 6.05 (d, ^3^*J* = 10.0 Hz, *H*NBoc), 5.84 (ddd, 1H, ^3^*J* = 1.4 Hz, ^3^*J* = 1.4 Hz, ^3^*J* = 7.7 Hz, ^3^*J* = 9.7 Hz, *H*C3), 4.88 (s, 1H, *H*COCH_3_), 4.58 (dddd, 1H, ^3^*J* = 3.2 Hz, ^3^*J* = 10.0 Hz, ^3^*J* = 7.7 Hz, ^3^*J* = 9.7 Hz, *H*C1), 4.09–3.97 (m, 4H, 2 × C*H*_2_OP), 3.95–3.84 (m, 2H, C*H*_2_OP), 3.69–3.57 (m, 2H, C*H*_2_OP), 3.54 (s, 3H, OC*H*_3_), 2.79–2.72 (m, 1H, *H_a_*C2). 2.67–2.61 (m, 1H, *H_b_*C2), 1.46 (s, 9H, 3 × C*H*_3_), 1.11 (t, 3H, ^3^*J* = 7.1 Hz, C*H*_3_CH_2_OP), 1.07 (t, 3H, ^3^*J* = 7.1 Hz, C*H*_3_CH_2_OP), 0.93 (t, 3H, ^3^*J* = 7.1 Hz, C*H*_3_CH_2_OP), 0.89 (t, 3H, ^3^*J* = 7.1 Hz, C*H*_3_CH_2_OP). ^31^P NMR (243 MHz, C_6_D_6_): δ = 24.72 (d, ^4^*J*_PCCCP_ = 7.9 Hz), 19.18 (d, ^4^*J*_PCCCP_ = 7.9 Hz). ^31^P NMR (243 MHz, CDCl_3_): δ = 24.34 (d, *J* = 7.9 Hz), 18.42 (d, *J* = 7.9 Hz).

Mandelate (1*S*,3*S*,1′*S*)-**16**: white amorphous solid; *t_R_* = 17.86 min. ^1^H NMR (C_6_D_6_, 600 MHz): δ = 7.62 (d, 2H, ^3^*J* = 7.4 Hz), 7.17–7.15 (m, 2H), 7.05 (t, 1H, ^3^*J* = 7.4 Hz), 5.87–5.83 (m, 1H, *H*C3), 5.26 (d, ^3^*J* = 10.4 Hz, *H*NBoc), 4.90 (s, 1H, *H*COCH_3_), 4.44–4.37 (m, 1H, *H*C1), 3.99–3.80 (m, 8H, 4 × C*H*_2_OP), 3.39 (s, 3H, OC*H*_3_), 2.84–2.77 (m, 1H, *H_a_*C2), 2.57–2.50 (m, 1H, *H_b_*C2), 1.43 (s, 9H, 3 × C*H*_3_), 1.03 (t, 6H, *J* = 7.0 Hz, 2 × C*H*_3_CH_2_OP), 0.97 (t, 3H, *J* = 7.0 Hz, C*H*_3_CH_2_OP), 0.91 (t, 3H, *J* = 7.0 Hz, C*H*_3_CH_2_OP). ^31^P NMR (C_6_D_6_, 243 MHz): δ = 24.31 (d, ^4^*J*_PCCCP_ = 7.5 Hz), 19.72 (d, ^4^*J*_PCCCP_ = 7.5 Hz). ^31^P NMR (CDCl_3_, 243 MHz): δ = 23.98 (d, ^4^*J*_PCCCP_ = 7.6 Hz), 19.34 (d, ^4^*J*_PCCCP_ = 7.6 Hz).

#### 3.8.2. Esterification of (1*R*,3*R*)-**6** and (1*R*,3*S*)-**6** with (*S*)-*O*-Methylmandelic Acid

From a 1:1 mixture of 3-hydroxydiphosphonates (1*R*,3*R*)-**6** and (1*R*,3*S*)-**6** (0.262 g, 0.585 mmol), (*S*)-*O*-methylmandelate (1*R*,3*R*,1′*S*)-**16** (0.010 g, 3%) was obtained followed by diastereoisomer (1*R*,3*S*,1′*S*)-**16** (0.010 g, 8%) after separation by HPLC with a mobile phase of water-acetonitrile (61.5:38.5, *v/v*) and a flow rate of 17 mL/min.

Mandelate (1*R*,3*R*,1′*S*)-**16**: white amorphous solid; *t_R_* = 12.56 min. ^31^P NMR (243 MHz, CDCl_3_): δ = 24.34 (d, *J* = 7.9 Hz), 18.42 (d, *J* = 7.9 Hz).

Mandelate (1*R*,3*S*,1′*S*)-**16**: colorless oil; *t_R_* = 14.12 min. ^1^H NMR (600 MHz, C_6_D_6_): δ = 7.63 (d, *J* = 7.5 Hz, 1H), 7.05 (t, *J* = 7.4 Hz, 1H), 5.96 (q, *J* = 7.8 Hz, 1H, *H*C3), 5.32 (d, *J* = 8.6 Hz, 1H, N*H*), 4.83 (s, 1H, *H*COCH_3_), 4.53 (dq, *J* = 16.6, 7.5 Hz, 1H), 3.28 (s, 1H, OC*H*_3_), 2.85–2.74 (m, 1H, *H_a_*C2), 2.30–2.17 (m, 1H, *H_b_*C2), 1.41 (s, 9H, 3 × C*H*_3_), 1.10–0.96 (m, 12H, 4 × C*H*_3_CH_2_OP). ^31^P NMR (243 MHz, C_6_D_6_): δ = 23.77, 19.72. ^31^P NMR (243 MHz, CDCl_3_): δ = 23.38, 19.14.

#### 3.8.3. Esterification of (1*S*,3*S*)-**6** and (1*S*,3*R*)-**6** with (*S*)-*O*-Methylmandelic Acid

From a mixture of 3-hydroxydiphosphonates (1*S*,3*S*)-**6** and (1*S*,3*R*)-**6** (0.088 g, 0.200 mmol), (*S*)-*O*-methylmandelate (1*S*,3*R*,1′*S*)-**16** (0.036 g, 36%) was obtained followed by diastereoisomer (1*S*,3*S*,1′*S*)-**16** (0.031 g, 31%) after separation by HPLC with a mobile phase of water-acetonitrile (60:40, *v/v*) at a flow rate of 17 mL/min.

Mandelate (1*S*,3*R*,1′*S*)-**16**: colourless oil; *t_R_* = 9.06 min. ^1^H NMR (600 MHz, C_6_D_6_): δ = 7.57 (d, *J* = 7.4 Hz, 2H), 7.11 (t, *J* = 7.4 Hz, 1H), 7.02 (t, *J* = 7.4 Hz, 1H), 5.97 (q, *J* = 8.0 Hz, 1H, *H*C3), 5.52 (d, *J =* 5.7 Hz, N*H*), 4.91 (s, 1H, *H*COCH_3_), 4.68–4.55 (m, 1H), 4.08–3.90 (m, 4H), 3.92–3.84 (m, 2H), 3.78 (qd, *J* = 7.4 Hz, *J* = 3.4 Hz, 1H), 3.72–3.61 (m, 1H), 3.38 (s, 3H, OC*H_3_*), 2.91–2.79 (m, 1H, *H_a_*C2), 2.40–2.25 (m, 1H, *H_b_*C2), 1.41 (s, 9H, 3 × C*H*_3_), 1.07 (t, *J* = 7.0 Hz, 3H, C*H*_3_CH_2_OP), 1.04 (t, *J* = 7.0 Hz, 3H, C*H*_3_CH_2_OP) 0.93 (t, *J* = 7.0 Hz, 6H, 2 × C*H*_3_CH_2_OP). ^31^P NMR (243 MHz, C_6_D_6_): δ = 24.00, 19.22. ^31^P NMR (243 MHz, CDCl_3_): δ = 23.61, 18.42.

Mandelate (1*S*,3*S*,1′*S*)-**16**: white amorphous solid; *t_R_* = 10.82 min. ^31^P NMR (CDCl_3_, 243 MHz): δ = 23.98 (d, ^4^*J*_PCCCP_ = 7.6 Hz), 19.34 (d, ^4^*J*_PCCCP_ = 7.6 Hz).

## 4. Conclusions

The nucleophilic addition reactions of aldehydes (*R*)-**7** and (*S*)-**7** with diethyl phosphite provided inseparable mixtures of diastereoisomeric diphosphonates (1*R*,3*S*)-**6** and (1*R*,3*R*)-**6**, and (1*S*,3*R*)-**6** and (1*S*,3*S*)-**6**, respectively. Diastereoisomeric 3-hydroxydiphosphonates **6** were then efficiently separated as *O*-acetates or *O*-*p*-nitrobenzoates and then hydrolysed to the designed phosphonic acids (1*R*,3*S*)-**5**, (1*R*,3*R*)-**5**, (1*S*,3*R*)-**5**, and (1*S*,3*S*)-**5** as diphosphonate analogues of 4-hydroxyglutamic acids.

## Data Availability

Data is contained within the article.

## Supplementary Materials

The following are available online at https://www.mdpi.com/article/10.3390/molecules27092699/s1, Figure S1: ^31^P NMR Spectrum for (1*R*,3*S*)-6 and (1*R*,3*R*)-6 in CDCl_3_, Figure S2: ^1^H NMR Spectrum for (1*R*,3*S*)-11 in CDCl_3_, Figure S3: ^13^C NMR Spectrum for (1*R*,3*S*)-11 in CDCl_3_, Figure S4: ^31^P NMR Spectrum for (1*R*,3*S*)-11 in CDCl_3_, Figure S5: ^1^H NMR Spectrum for (1*R*,3*R*)-11 in CDCl_3_, Figure S6: ^13^C NMR Spectrum for (1*R*,3*R*)-11 in CDCl_3_, Figure S7: ^31^P NMR Spectrum for (1*R*,3*R*)-11 in CDCl_3_, Figure S8: ^1^H NMR Spectrum for (1*R*,3*S*)-12 in CDCl_3_, Figure S9: ^13^C NMR Spectrum for (1*R*,3*S*)-12 in CDCl_3_, Figure S10: ^31^P NMR Spectrum for (1*R*,3*S*)-12 in CDCl_3_, Figure S11: ^1^H NMR Spectrum for (1*R*,3*R*)-12 in CDCl_3_, Figure S12: ^13^C NMR Spectrum for (1*R*,3*R*)-12 in CDCl_3_, Figure S13: ^31^P NMR Spectrum for (1*R*,3*R*)-12 in CDCl_3_, Figure S14: ^1^H NMR Spectrum for (1*R*,3*R*)-4 in D_2_O, Figure S15: ^13^C NMR Spectrum for (1*R*,3*R*)-4 in D_2_O, Figure S16: ^31^P NMR Spectrum for (1*R*,3*R*)-4 in D_2_O, Figure S17: ^1^H NMR Spectrum for (1*R*,3*S*)-4 in D_2_O, Figure S18: ^13^C NMR Spectrum for (1*R*,3*S*)-4 in D_2_O, Figure S19: ^31^P NMR Spectrum for (1*R*,3*S*)-4 in D_2_O, Figure S20: ^1^H NMR Spectrum for *trans*-14 in CDCl_3_, Figure S21: ^13^C NMR Spectrum for *trans*-14 in CDCl_3_, Figure S22: ^31^P NMR Spectrum for *trans*-14 in CDCl_3_, Figure S23: ^1^H NMR Spectrum for *trans*-15 in CDCl_3_, Figure S24: ^13^C NMR Spectrum for *trans*-15 in CDCl_3_, Figure S25: ^31^P NMR Spectrum for *trans*-15 in CDCl_3_, Figure S26: ^1^H NMR Spectrum for (1*R/S*,3*R/S*)-6 [*anti*-6] in CDCl_3_, Figure S27: ^13^C NMR Spectrum for (1*R/S*,3*R/S*)-6 [*anti*-6] in CDCl_3_, Figure S28: ^31^P NMR Spectrum for (1*R/S*,3*R/S*)-6 [*anti*-6] in CDCl_3_, Figure S29: ^1^H NMR Spectrum for (1*R/S*,3*R/S*)-6 [*anti*-6] in C_6_D_6_, Figure S30: ^13^C NMR Spectrum for (1*R/S*,3*R/S*)-6 [*anti*-6] in C_6_D_6_, Figure S31: ^31^P NMR Spectrum for (1*R/S*,3*R/S*)-6 [*anti*-6] in C_6_D_6_, Figure S32: 1H NMR Spectrum for (1*R*,3*R*,1’*S*)-16 in C_6_D_6_, Figure S33: ^31^P NMR Spectrum for (1*R*,3*R*,1’*S*)-16 in C_6_D_6_, Figure S34: ^31^P NMR Spectrum for (1*R*,3*R*,1’*S*)-16 in CDCl_3_, Figure S35: 1H NMR Spectrum for (1*S*,3*S*,1’*S*)-16 in C_6_D_6_, Figure S36: ^31^P NMR Spectrum for (1*S*,3*S*,1’*S*)-16 in C_6_D_6_, Figure S37: ^31^P NMR Spectrum for (1*S*,3*S*,1’*S*)-16 in CDCl_3_, Figure S38: ^1^H NMR Spectrum for (1*R*,3*S*,1’*S*)-16 in C_6_D_6_, Figure S39: ^31^P NMR Spectrum for (1*R*,3*S*,1’*S*)-16 in C_6_D_6_, Figure S40: ^31^P NMR Spectrum for (1*R*,3*S*,1’*S*)-16 in CDCl_3_, Figure S41: ^1^H NMR Spectrum for (1*S*,3*R*,1’*S*)-16 in C_6_D_6_, Figure S42: ^31^P NMR Spectrum for (1*S*,3*R*,1’*S*)-16 in C_6_D_6_, Figure S43: ^31^P NMR Spectrum for (1*S*,3*R*,1’*S*)-16 in CDCl_3_, Figure S44: Separation of (1*R*,3*S*)-11 and (1*R*,3*R*)-11 by preparative HPLC, Figure S45: Separation of (1*S*,3*R*)-11 and (1*S*,3*S*)-11 by preparative HPLC, Figure S46: Separation of (1*R*,3*S*)-12 and (1*R*,3*R*)-12 by preparative HPLC, Figure S47: Separation of (1*S*,3*R*)-12 and (1*S*,3*S*)-12 by preparative HPLC, Figure S48: Separation of *trans*-14 and *trans*-15 by preparative HPLC, Figure S49: Separation of (1*R*,3*R*,1’*S*)-16 and (1*S*,3*S*,1’*S*)-16 by preparative HPLC, Figure S50: Separation of (1*R*,3*R*,1’*S*)-16 and (1*R*,3*S*,1’*S*)-16 by preparative HPLC, Figure S51: Separation of (1*S*,3*R*,1’*S*)-16 and (1*S*,3*S*,1’*S*)-16 by preparative HPLC.

## Sample Availability

Samples are not available.

## Abbreviations

DCC	N,N′-dicyclohexylcarbodiimide
DCU	dicyclohexylurea
DMAP	4-dimethylaminopyridine
HPLC	high performance liquid chromatography
L-AP4	L-(+)-2-amino-4-phosphonobutyric acid
MgBr_2_-etherate	magnesium bromide ethyl etherate
mGluR	glutamate metabotropic receptors
mGlu_1a_R	metabotropic glutamate 1a receptor
mGlu_8a_R	metabotropic glutamate 8a receptor
MAP4	(*S*)-2-Amino-2-methyl-4-phosphonobutyric acid
NMDA	N-methyl-D-aspartic acid
rt	room temperature
