# Supplementary material for: Synthesis of Four Enantiomers of (1-Amino-3-Hydroxypropane-1,3-Diyl)Diphosphonic Acid as Diphosphonate Analogues of 4-Hydroxyglutamic Acid
Source: Molecules. 2022 Apr 22;27(9):2699. doi: 10.3390/molecules27092699 (PMC9105571; doi:10.3390/molecules27092699)
Supplement: Supplementary file 1 [file molecules-27-02699-s001.zip › molecules-1691647-supplementary.pdf]

Synthesis of four enantiomers of (1-amino-3-hydroxypropane-1,3-diyl)diphosphonic acid as diphosphonate analogues of 4-hydroxyglutamic acid

Liwia Lebelt [1], Iwona E. Głowacka [1] and Dorota G. Piotrowska [1,*]

1. Bioorganic Chemistry Laboratory, Faculty of Pharmacy, Medical University of Lodz, 90-151 Lodz, Muszynskiego 1, Poland; liwia.lebelt@umed.lodz.pl (L.L.); iwona.glowacka@umed.lodz.pl (I.E.G.); dorota.piotrowska@umed.lodz.pl (D.G.P.)

* Correspondence:; dorota.piotrowska@umed.lodz.pl (D.G.P.)

Contents

NMR spectra for compounds (1*R*,3*S*)-**6** and (1*R*,3*R*)-**6**, (1R,3S)-**11**, (1*R*,3*R*)-**11,** (1*R*,3*S*)-**12,** (1*R*,3*S*)-**12,** (1*R*,3*R*)-**4**, (1*R*,3*S*)-**4**, **14**, **15**, (1*R*/*S*,3*R*/*S*)-**6** [*anti*-**6**], (1*R*,3*R*,1′*S*)-**16**, (1*S*,3*S*,1′*S*)-**16**, (1*S*,3*R*,1′*S*)-**16**.

Preparative chromatograms for (1*R*,3*S*)-**11** and (1*R*,3R)-**11,** (1*S*,3*R*)-**12** and (1*S*,3*S*)-**12**, (1*S*,3*R*)-**12** and (1*S*,3*S*)-**12**, (1*R*,3*R*,1′*S*)-**16** and (1*S*,3*S*,1′*S*)-**16**, **14** and **15**, (1*R*,3*R*,1′*S*)-**16** and (1*R*,3*S*,1′*S*)-**16**, (1*S*,3*R*,1′*S*)-**16** and (1*S*,3*S*,1′*S*)-**16.**

Figure S1: ^{31}P NMR Spectrum for (1*R*,3*S*)-**6** and (1*R*,3*R*)-**6** in $CDCl_3$

Figure S2: ^{1}H NMR Spectrum for (1*R*,3*S*)-**11** in $CDCl_3$

Figure S3: ^{13}C NMR Spectrum for (1*R*,3*S*)-**11** in $CDCl_3$

Figure S4: ^{31}P NMR Spectrum for (1*R*,3*S*)-**11** in $CDCl_3$

Figure S5: ^{1}H NMR Spectrum for (1*R*,3*R*)-**11** in $CDCl_3$

Figure S6: ^{13}C NMR Spectrum for (1*R*,3*R*)-**11** in $CDCl_3$

Figure S7: ^{31}P NMR Spectrum for (1*R*,3*R*)-**11** in $CDCl_3$

Figure S8: ^{1}H NMR Spectrum for (1*R*,3S)-**12** in $CDCl_3$

Figure S9: ^{13}C NMR Spectrum for (1*R*,3*S*)-**12** in $CDCl_3$

Figure S10: ^{31}P NMR Spectrum for (1*R*,3*S*)-**12** in $CDCl_3$

Figure S11: ^{1}H NMR Spectrum for (1*R*,3*R*)-**12** in $CDCl_3$

Figure S12: ^{13}C NMR Spectrum for (1*R*,3*R*)-**12** in $CDCl_3$

Figure S13: ^{31}P NMR Spectrum for (1*R*,3*R*)-**12** in $CDCl_3$

Figure S14: ^{1}H NMR Spectrum for (1*R*,3*R*)-**4** in D_2O

Figure S15: ^{13}C NMR Spectrum for (1*R*,3*R*)-**4** in D_2O

Figure S16: ^{31}P NMR Spectrum for (1*R*,3*R*)-**4** in D_2O

Figure S17: ^{1}H NMR Spectrum for (1*R*,3*S*)-**4** in D_2O

Figure S18: ^{13}C NMR Spectrum for (1*R*,3*S*)-**4** in D_2O

Figure S19: ^{31}P NMR Spectrum for (1*R*,3*S*)-**4** in D_2O

Figure S20: ^{1}H NMR Spectrum for *trans*-**14** in $CDCl_3$

Figure S21: ^{13}C NMR Spectrum for *trans*-**14** in $CDCl_3$

Figure S22: ^{31}P NMR Spectrum for *trans*-**14** in $CDCl_3$

Figure S23: ^{1}H NMR Spectrum for *trans*-**15** in $CDCl_3$

Figure S24: ^{13}C NMR Spectrum for *trans*-**15** in $CDCl_3$

Figure S25: ^{31}P NMR Spectrum for *trans*-**15** in $CDCl_3$

Figure S26: ^{1}H NMR Spectrum for (1*R*/*S*,3*R*/*S*)-**6** [*anti*-**6**] in $CDCl_3$

Figure S27: ^{13}C NMR Spectrum for (1*R*/*S*,3*R*/*S*)-**6** [*anti*-**6**] in $CDCl_3$

Figure S28: ^{31}P NMR Spectrum for (1*R*/*S*,3*R*/*S*)-**6** [*anti*-**6**] in $CDCl_3$

Figure S29: ^{1}H NMR Spectrum for (1*R*/*S*,3*R*/*S*)-**6** [*anti*-**6**] in C_6D_6

Figure S30: ^{13}C NMR Spectrum for (1*R*/*S*,3*R*/*S*)-**6** [*anti*-**6**] in C_6D_6

Figure S31: ^{31}P NMR Spectrum for (1*R*/*S*,3*R*/*S*)-**6** [*anti*-**6**] in C_6D_6

Figure S32: ^{1}H NMR Spectrum for (1*R*,3*R*,1′*S*)-**16** in C_6D_6

Figure S33: ^{31}P NMR Spectrum for (1*R*,3*R*,1′*S*)-**16** in C_6D_6

Figure S34: ^{31}P NMR Spectrum for (1*R*,3*R*,1′*S*)-**16** in $CDCl_3$

Figure S35: ^{1}H NMR Spectrum for (1*S*,3*S*,1′*S*)-**16** in C_6D_6

Figure S36: ^{31}P NMR Spectrum for (1*S*,3*S*,1′*S*)-**16** in C_6D_6

Figure S37: ^{31}P NMR Spectrum for (1*S*,3*S*,1′*S*)-**16** in $CDCl_3$

Figure S38: ^{1}H NMR Spectrum for (1*R*,3*S*,1′*S*)-**16** in C_6D_6

Figure S39: ^{31}P NMR Spectrum for (1*R*,3*S*,1′*S*)-**16** in C_6D_6

Figure S40: ^{31}P NMR Spectrum for (1*R*,3*S*,1′*S*)-**16** in $CDCl_3$

Figure S41: ^{1}H NMR Spectrum for (1*S*,3*R*,1′*S*)-**16** in C_6D_6

Figure S42: ^{31}P NMR Spectrum for (1*S*,3*R*,1′*S*)-**16** in C_6D_6

Figure S43: ^{31}P NMR Spectrum for (1*S*,3*R*,1′*S*)-**16** in $CDCl_3$

Figure S44: Separation of (1*R*,3*S*)-**11** and (1*R*,3R)-**11** by preparative HPLC

Figure S45: Separation of (1*S*,3*R*)-**11** and (1*S*,3*S*)-**11** by preparative HPLC

Figure S46: Separation of (1*R*,3*S*)-**12** and (1*R*,3*R*)-**12** by preparative HPLC

Figure S47: Separation of (1*S*,3*R*)-**12** and (1*S*,3*S*)-**12** by preparative HPLC

Figure S48: Separation of *trans*-**14** and *trans*-**15** by preparative HPLC

Figure S49: Separation of (1*R*,3*R*,1′*S*)-**16** and (1*S*,3*S*,1′*S*)-**16** by preparative HPLC

Figure S50: Separation of (1*R*,3*R*,1′*S*)-**16** and (1*R*,3*S*,1′*S*)-**16** by preparative HPLC

Figure S51: Separation of (1*S*,3*R*,1′*S*)-**16** and (1*S*,3*S*,1′*S*)-**16** by preparative HPLC

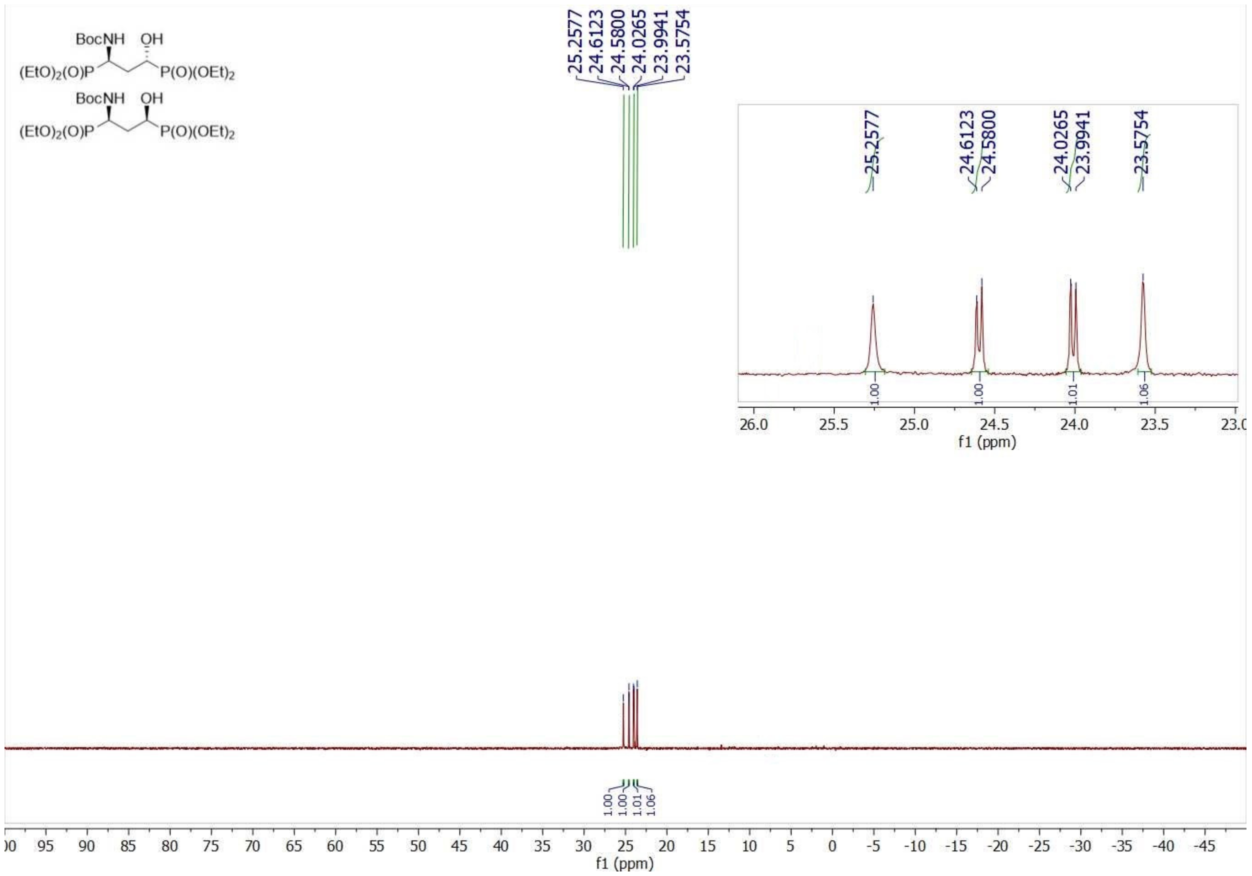

Figure S1: ^{31}P NMR Spectrum for (1*R*,3*S*)-**6** and (1*R*,3*R*)-**6** in $CDCl_3$

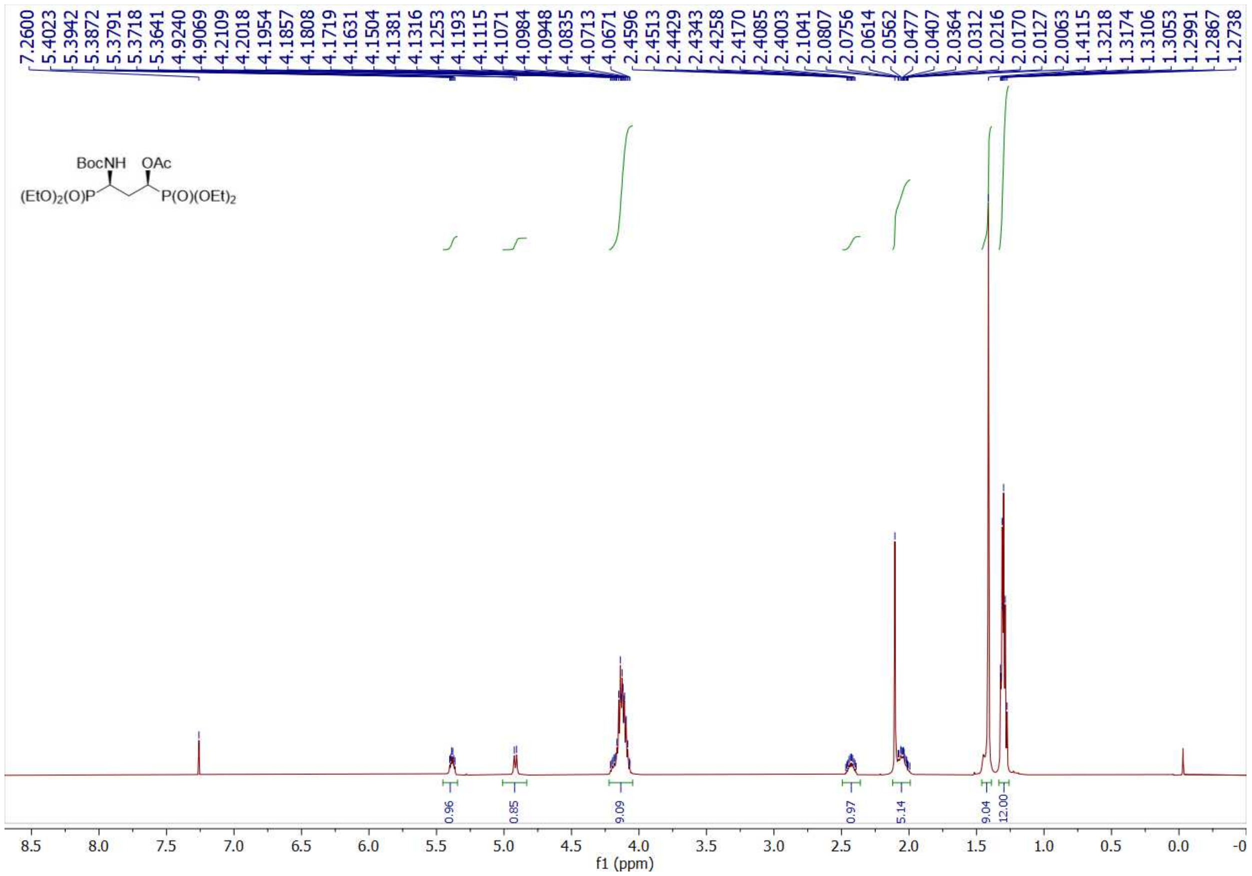

Figure S2: ^{1}H NMR Spectrum for (1*R*,3*S*)-**11** in $CDCl_3$

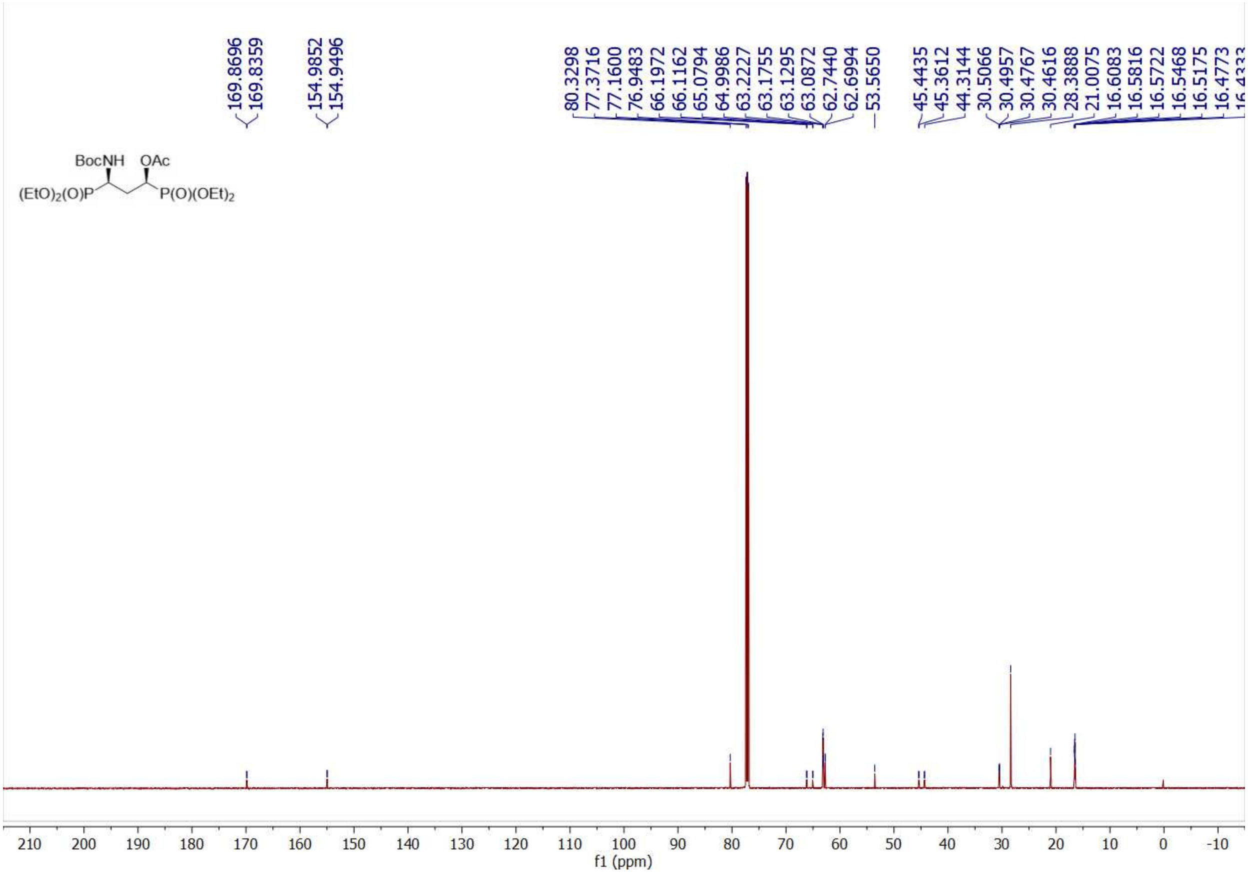

Figure S3: ^{13}C NMR Spectrum for (1*R*,3*S*)-**11** in $CDCl_3$

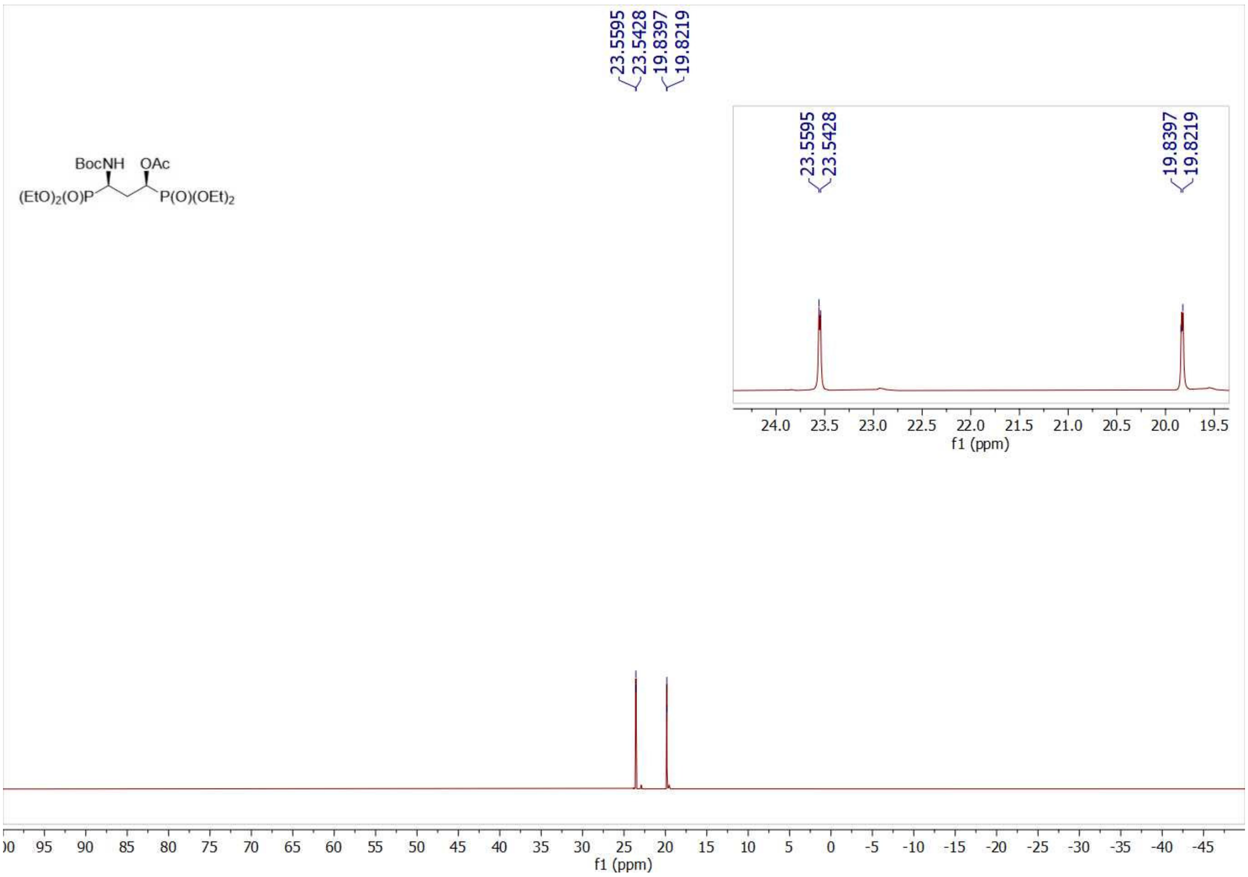

Figure S4: ^{31}P NMR Spectrum for (1*R*,3*S*)-**11** in $CDCl_3$

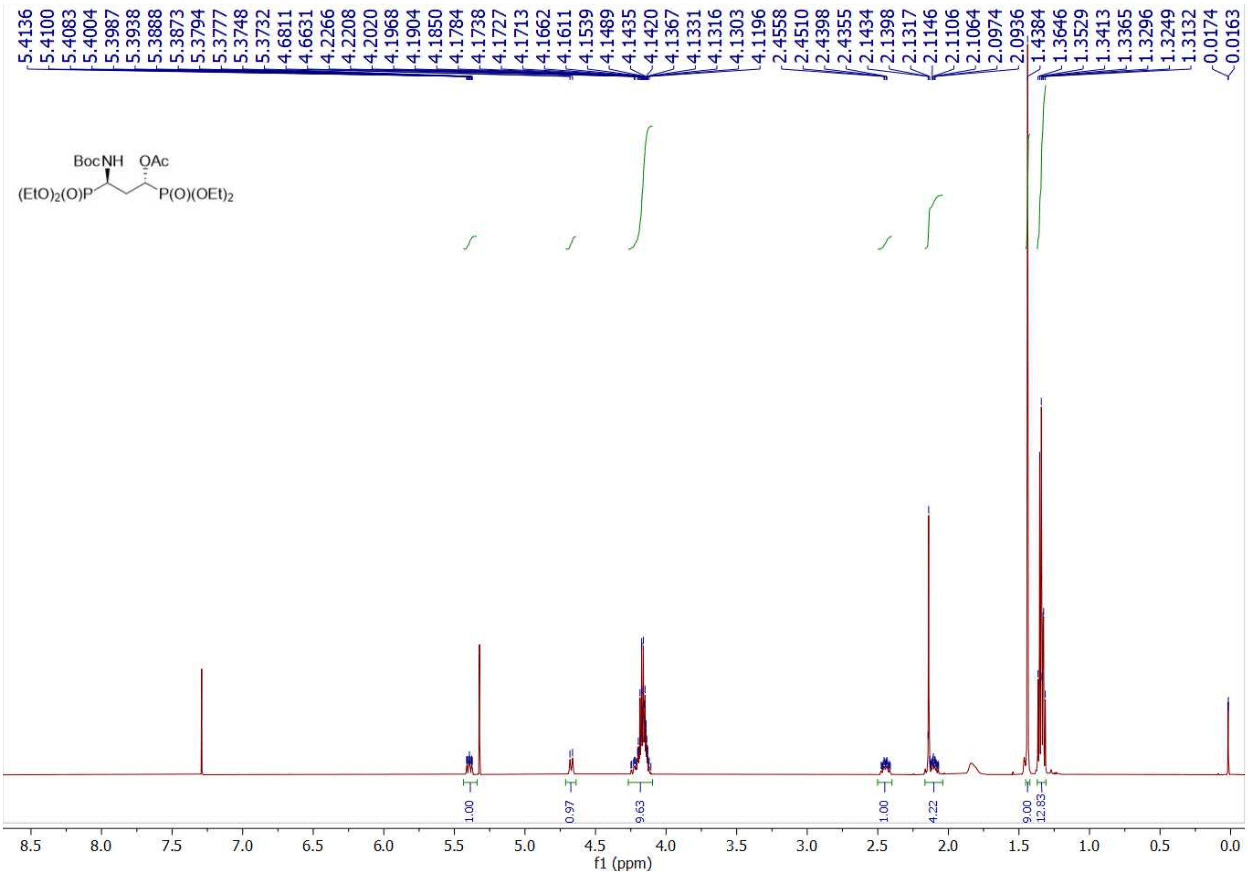

Figure S5: ^{1}H NMR Spectrum for (1*R*,3*R*)-**11** in $CDCl_3$

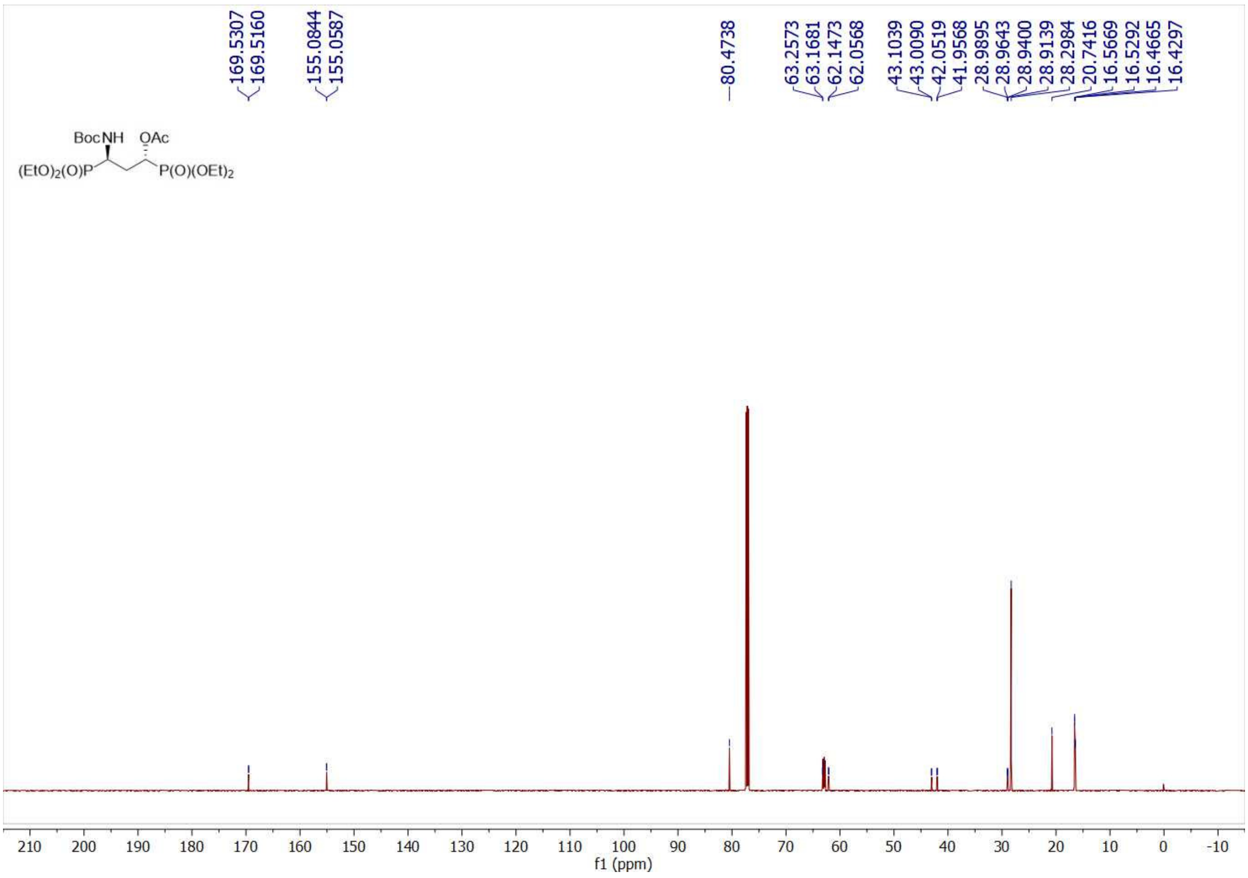

Figure S6: ^{13}C NMR Spectrum for (1*R*,3*R*)-**11** in $CDCl_3$

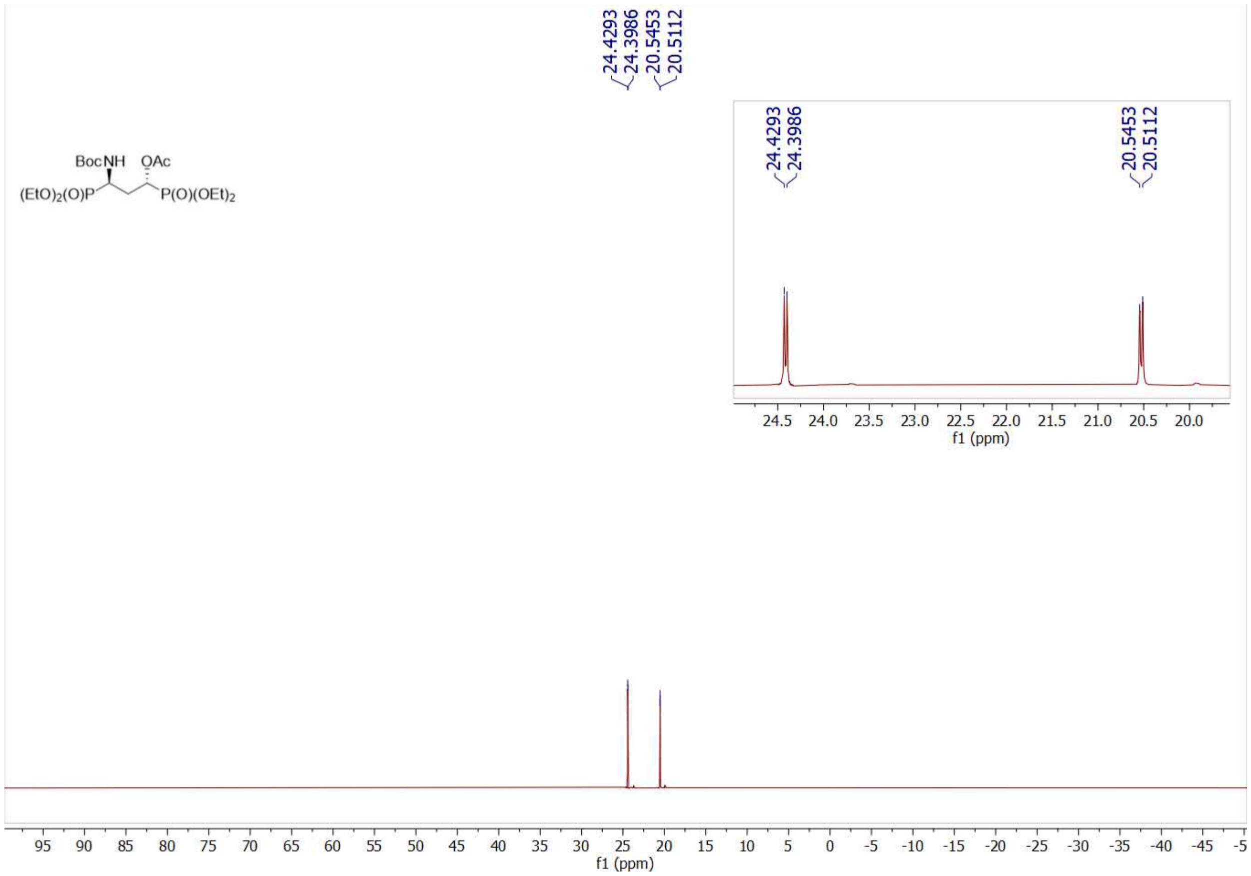

Figure S7: ^{31}P NMR Spectrum for (1*R*,3*R*)-**11** in $CDCl_3$

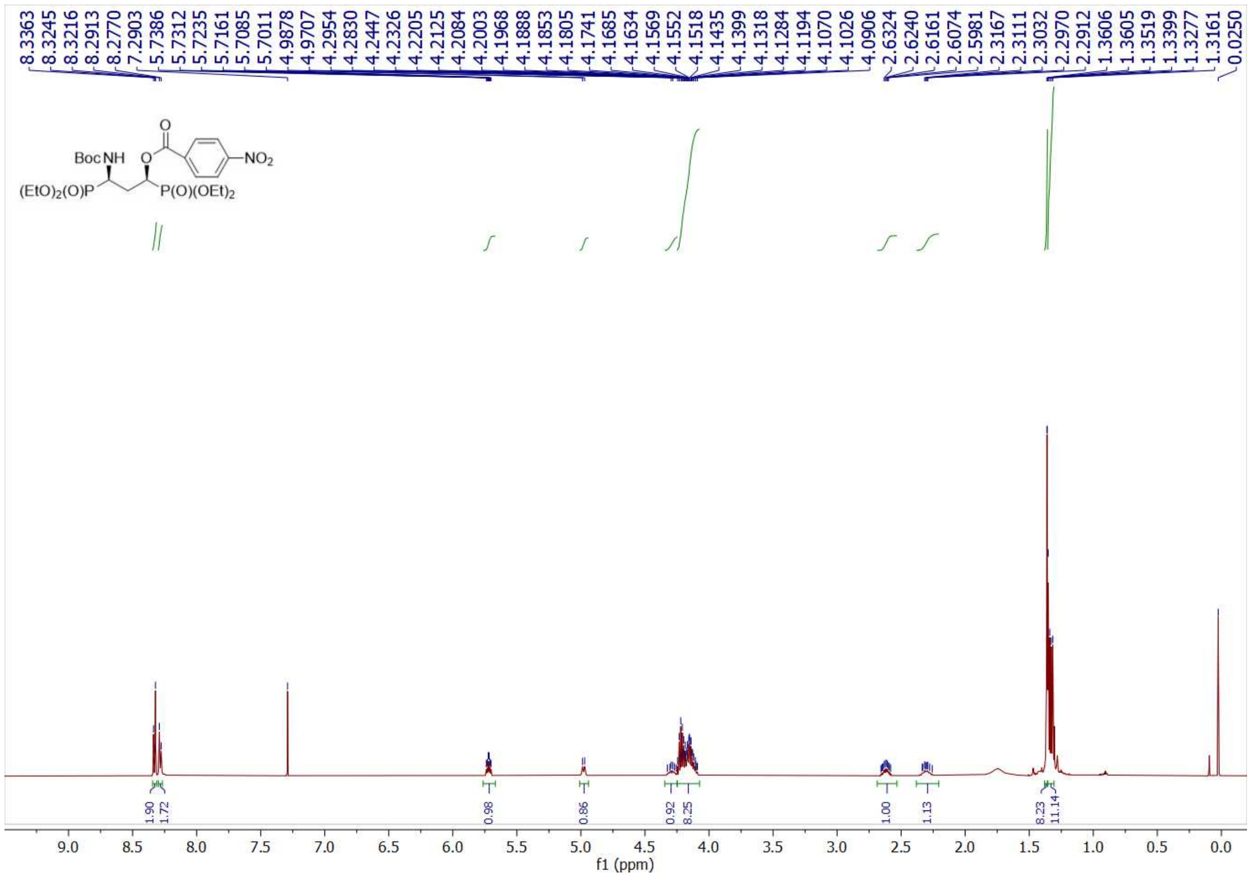

Figure S8: ^{1}H NMR Spectrum for (1*R*,3*S*)-**12** in $CDCl_3$

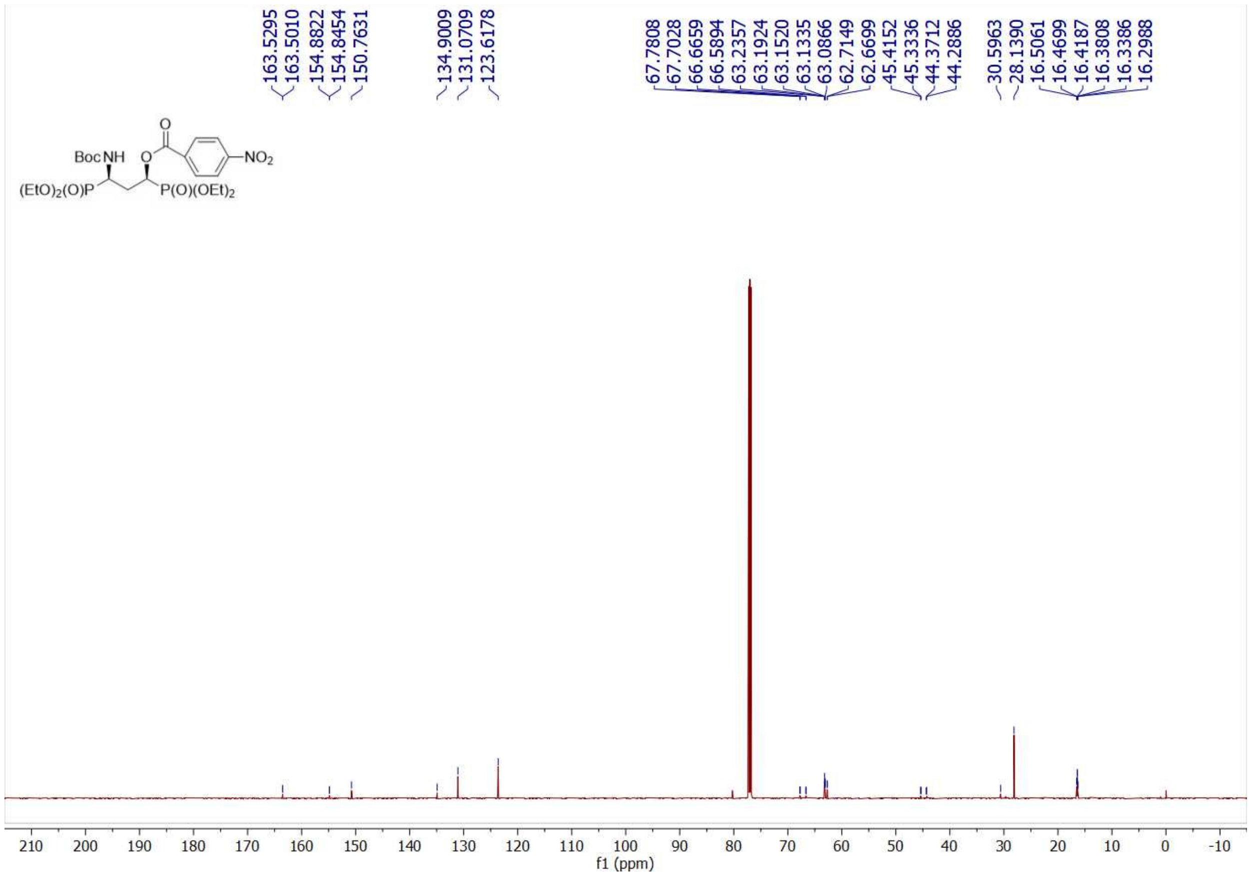

Figure S9: ^{13}C NMR Spectrum for (1*R*,3*S*)-**12** in $CDCl_3$

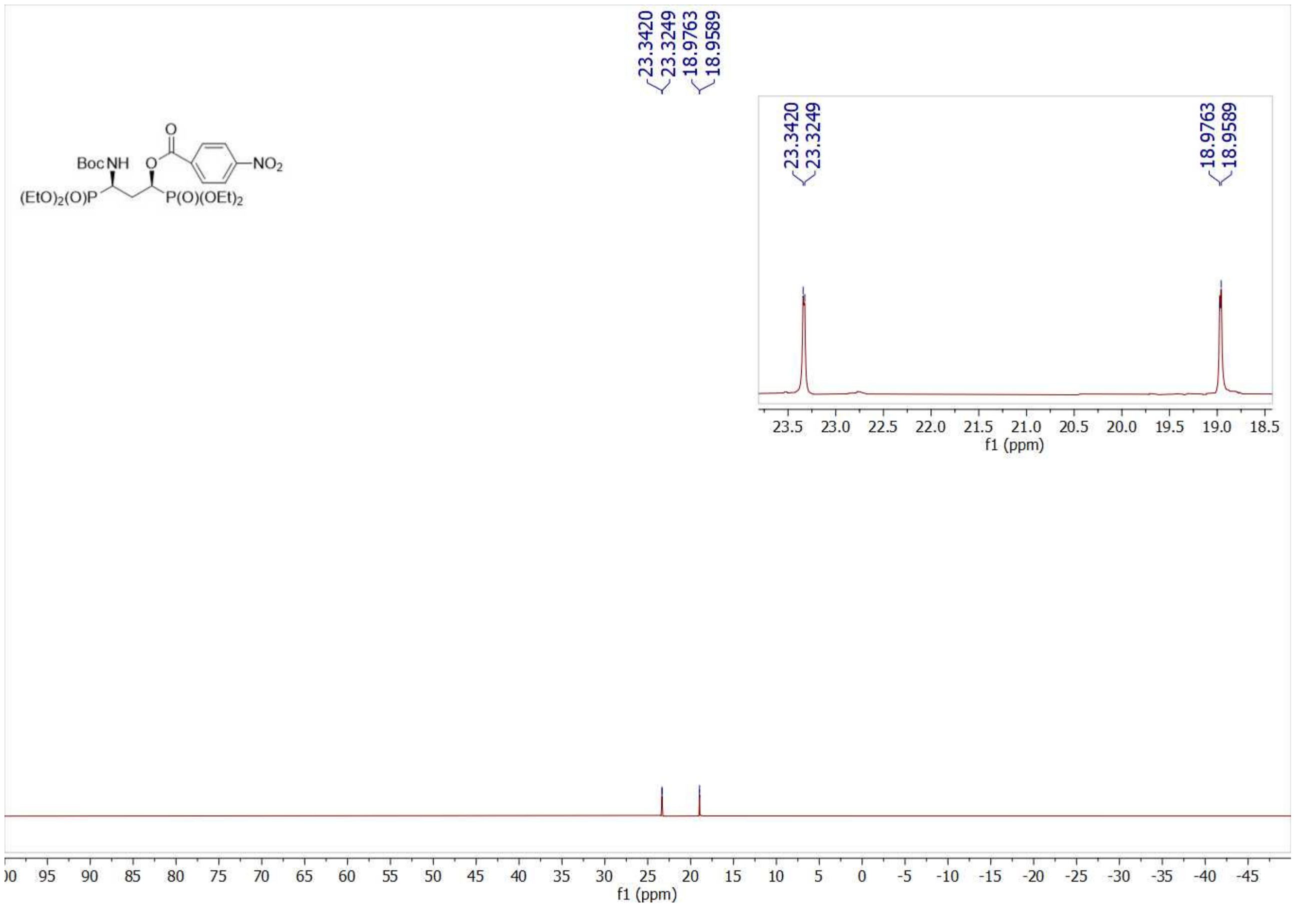

Figure S10: ^{31}P NMR Spectrum for (1*R*,3*S*)-**12** in $CDCl_3$

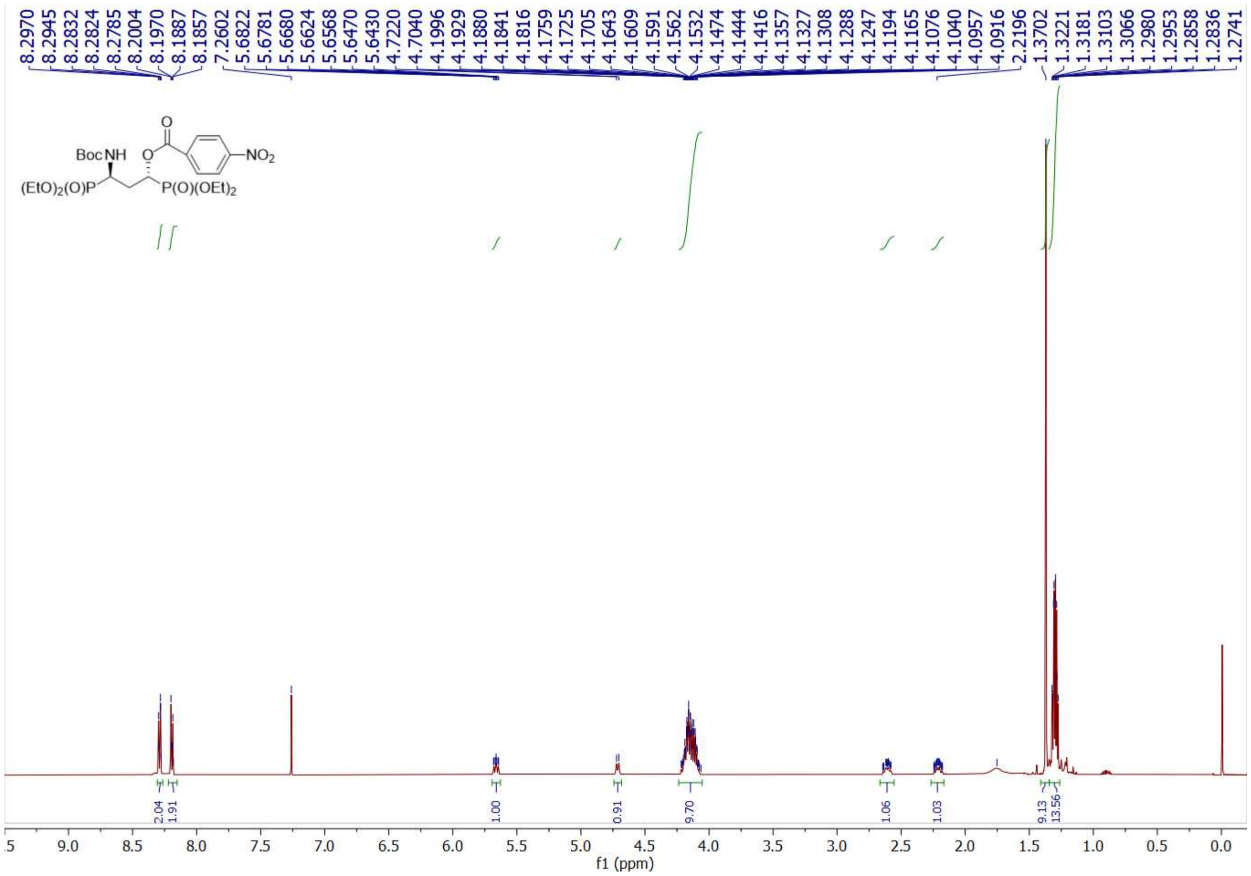

Figure S11: ^{1}H NMR Spectrum for (1*R*,3*R*)-**12** in CDCl3

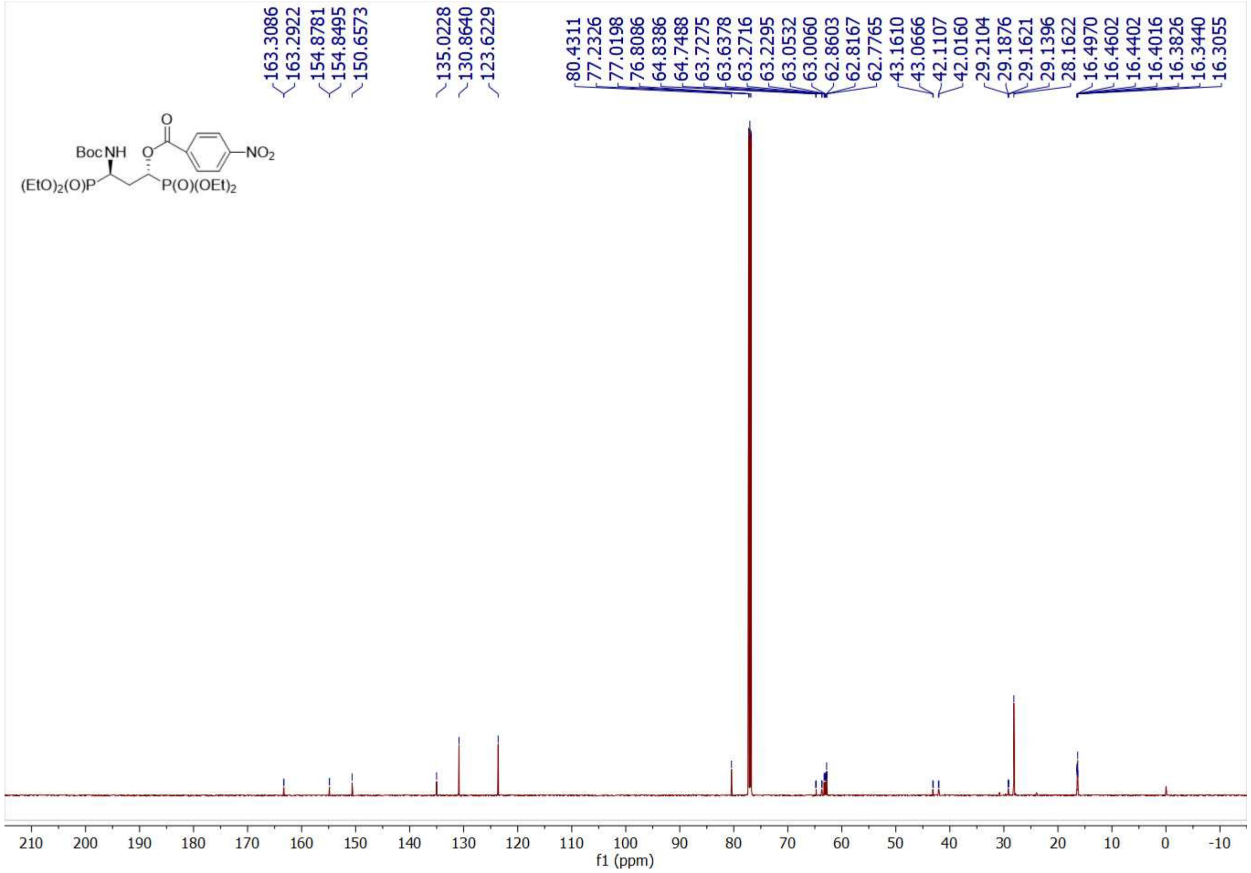

Figure S12: ^{13}C NMR Spectrum for (1*R*,3*R*)-**12** in CDCl3

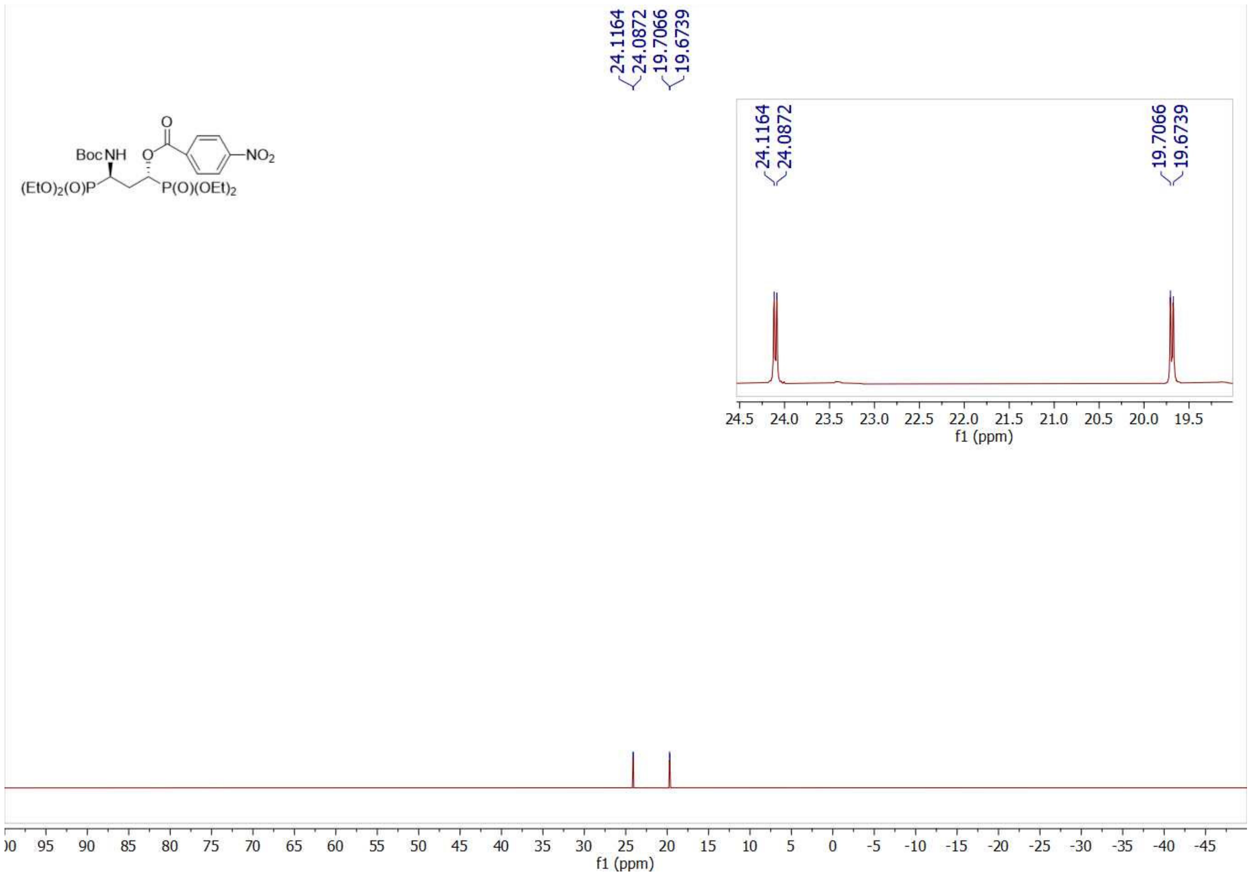

Figure S13: ^{31}P NMR Spectrum for (1*R*,3*R*)-**12** in $CDCl_3$

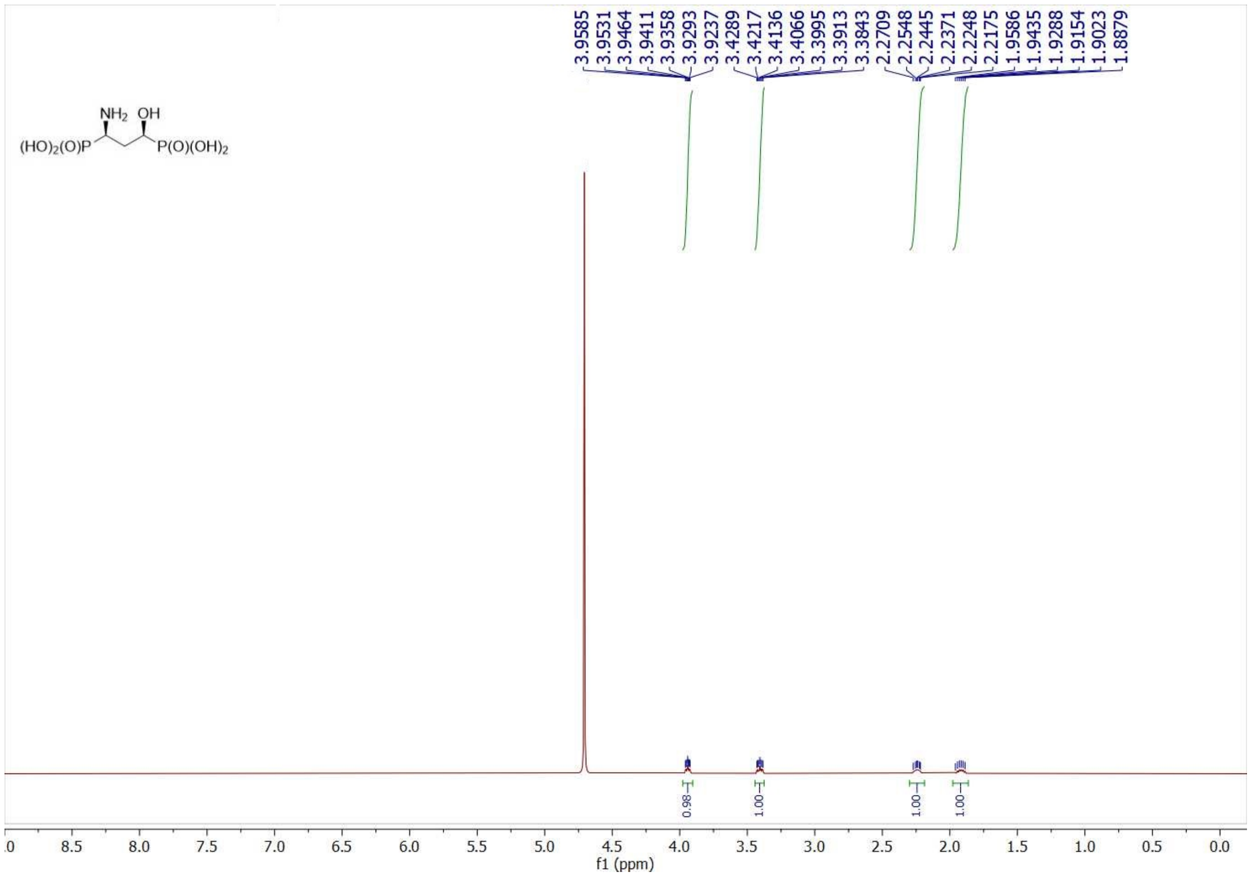

Figure S14: ^{1}H NMR Spectrum for (1*R*,3*R*)-**4** in D_2O

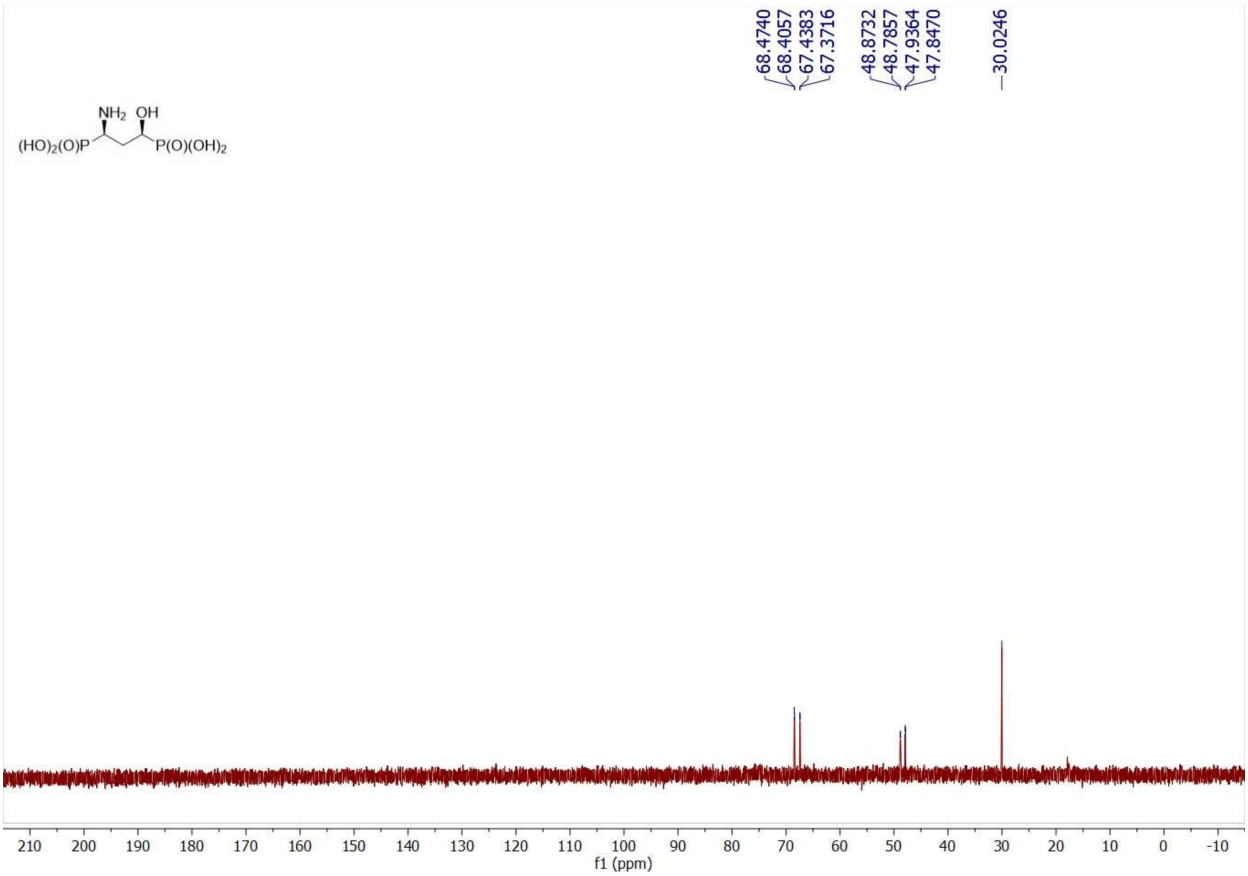

Figure S15: ^{13}C NMR Spectrum for (1*R*,3*R*)-**4** in D_2O

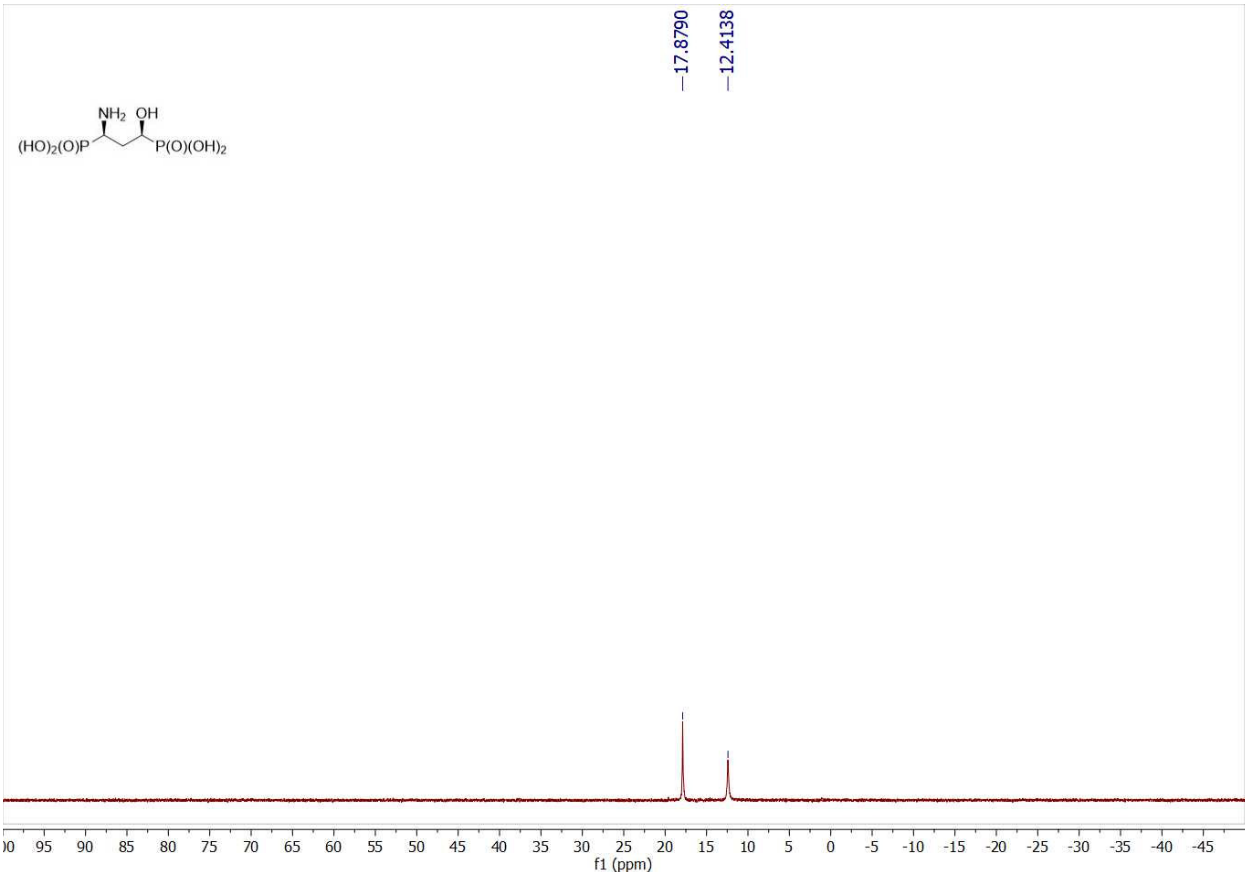

Figure S16: ^{31}P NMR Spectrum for (1*R*,3*R*)-**4** in D_2O

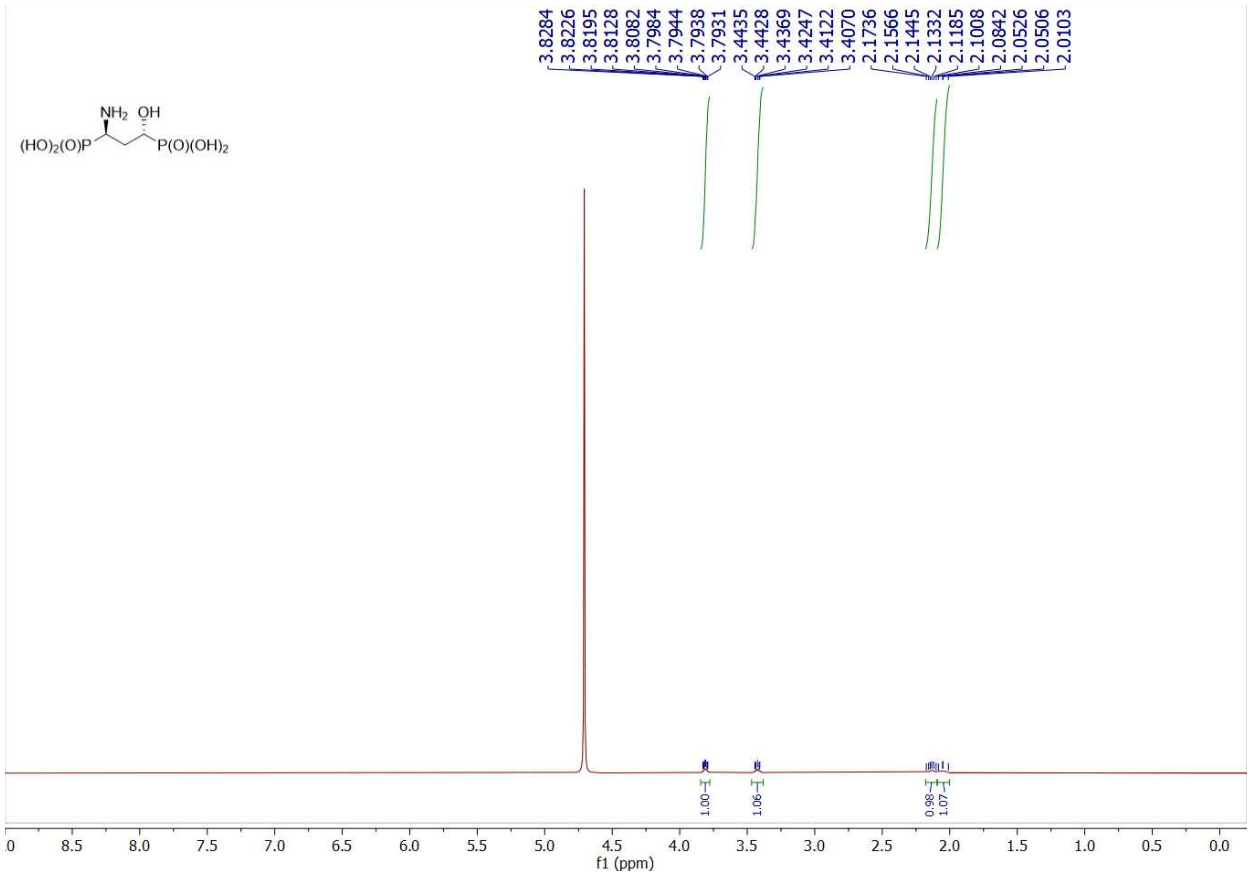

Figure S17: ^{1}H NMR Spectrum for (1*R*,3*S*)-**4** in D_2O

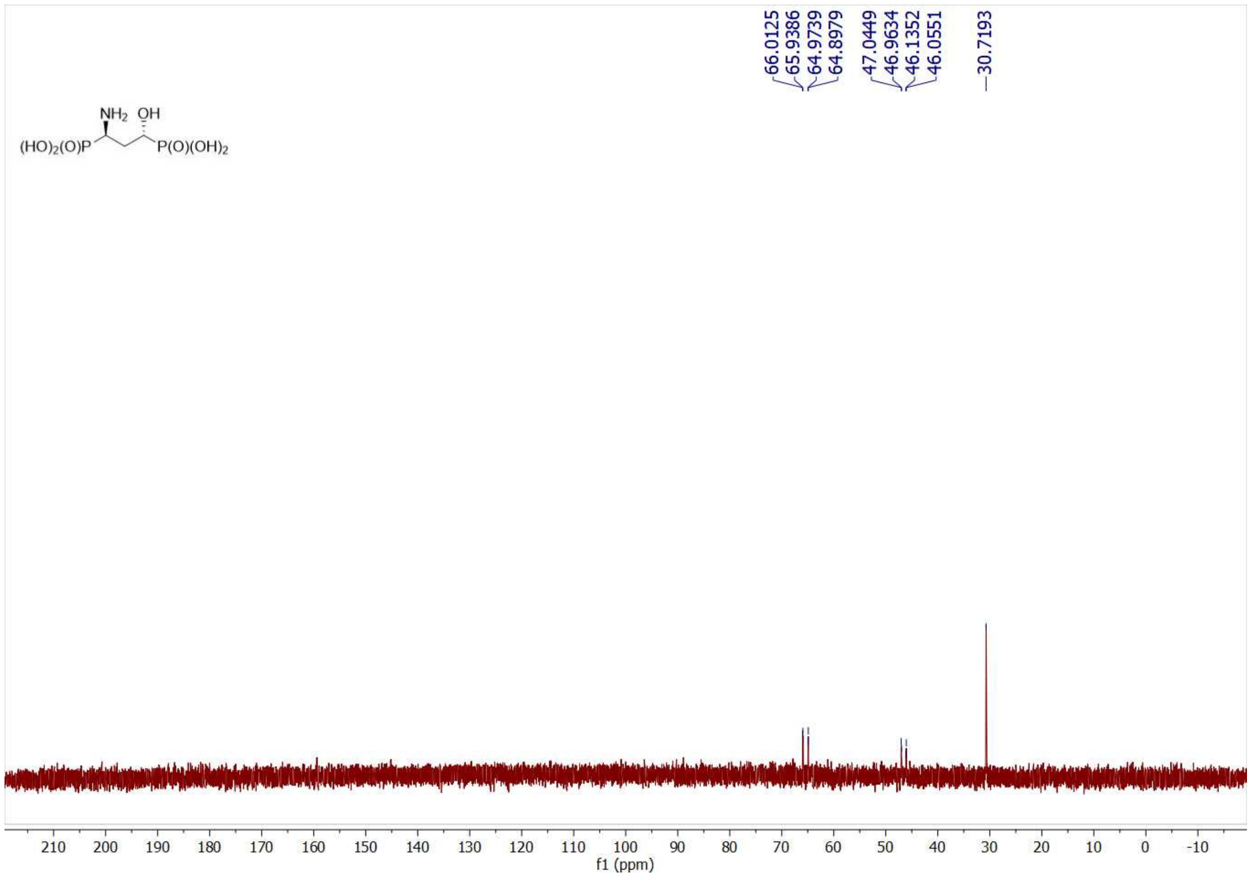

Figure S18: ^{13}C NMR Spectrum for (1*R*,3*S*)-**4** in D_2O

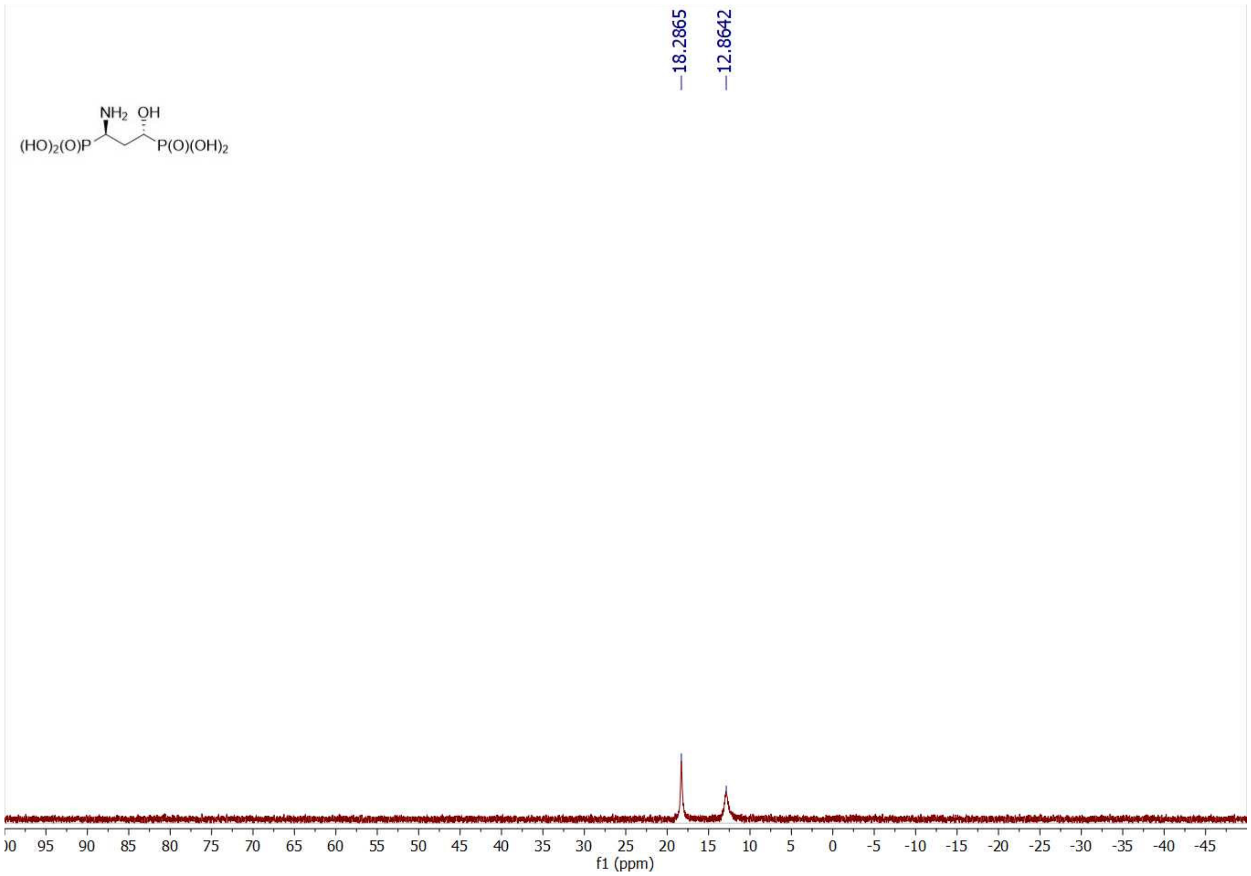

Figure S19: ^{31}P NMR Spectrum for (1*R*,3*S*)-**4** in D_2O

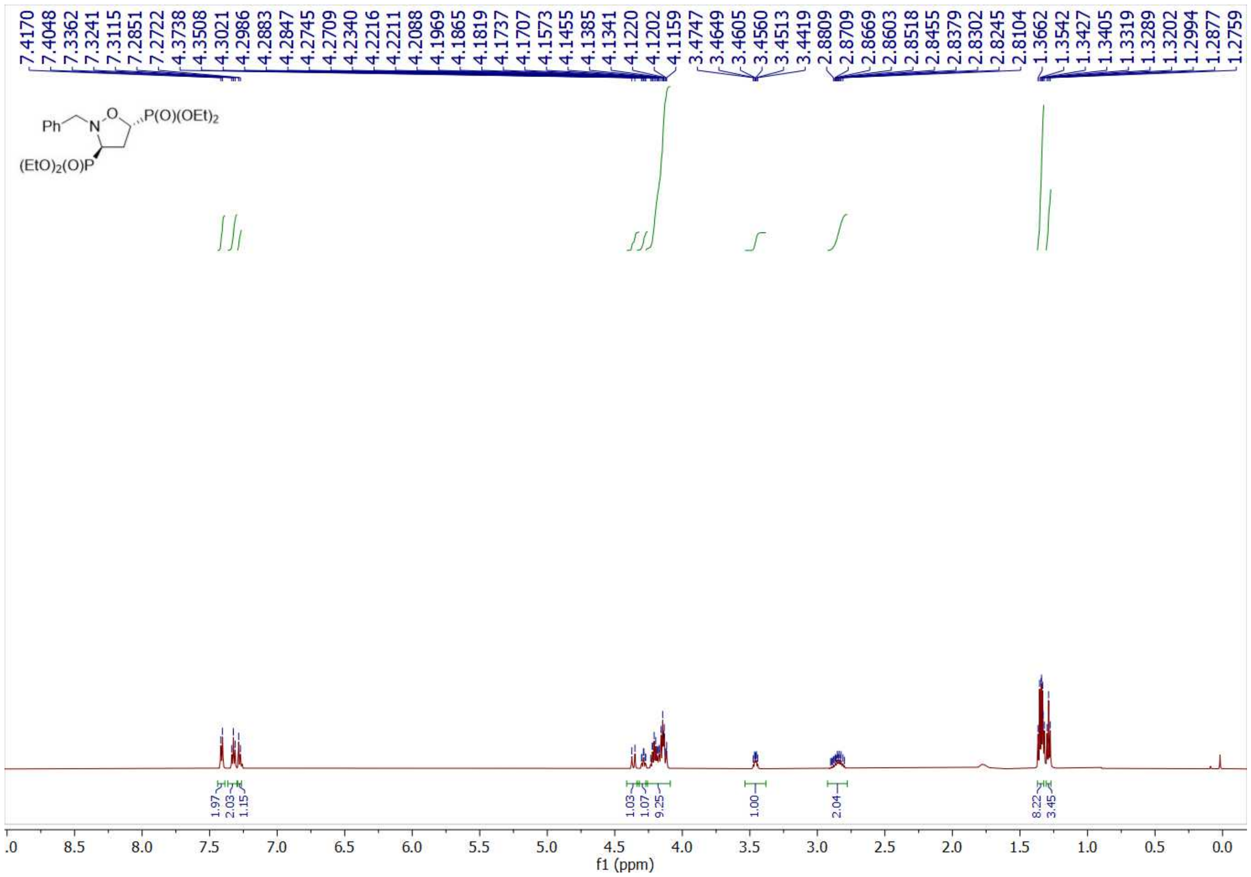

Figure S20: ^{1}H NMR Spectrum for *trans*-**14** in $CDCl_3$

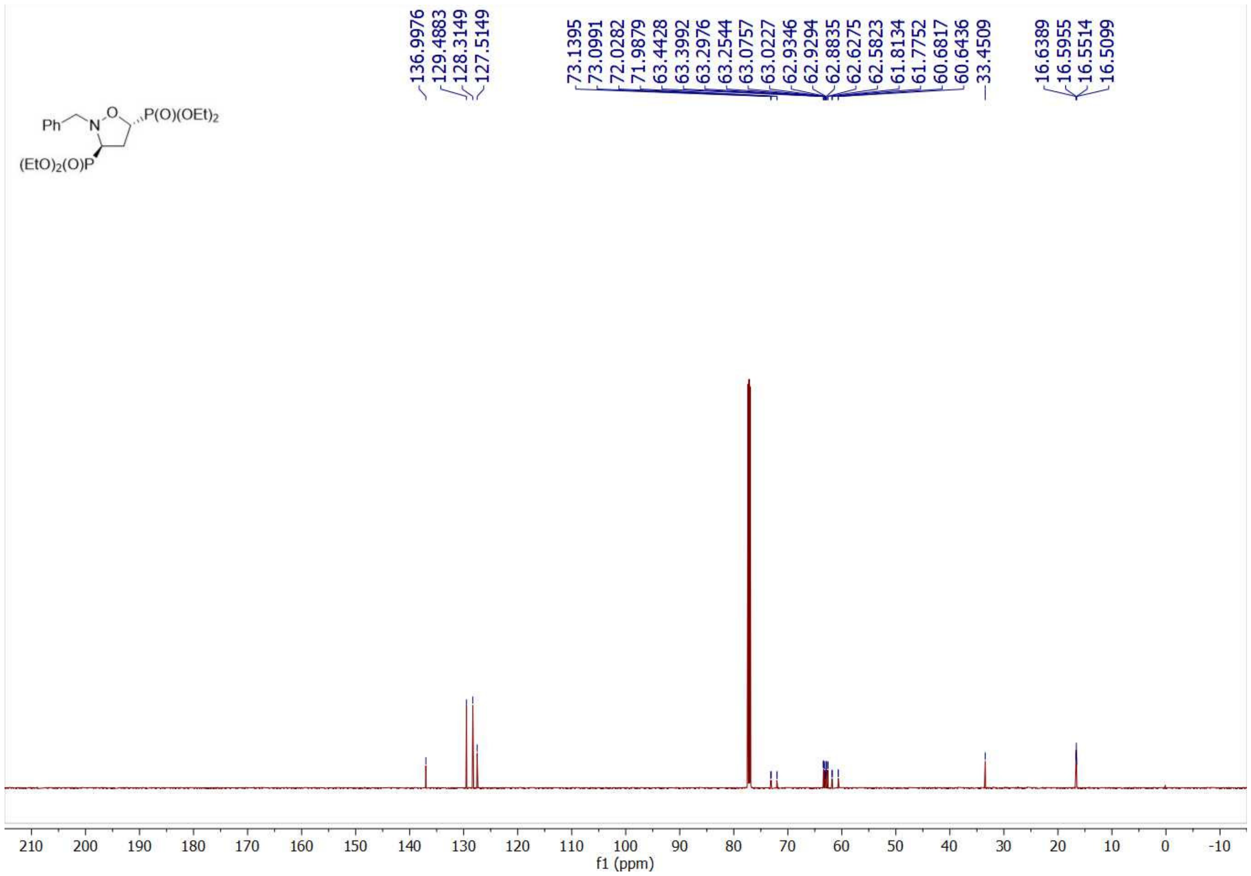

Figure S21: ^{13}C NMR Spectrum for *trans*-**14** in $CDCl_3$

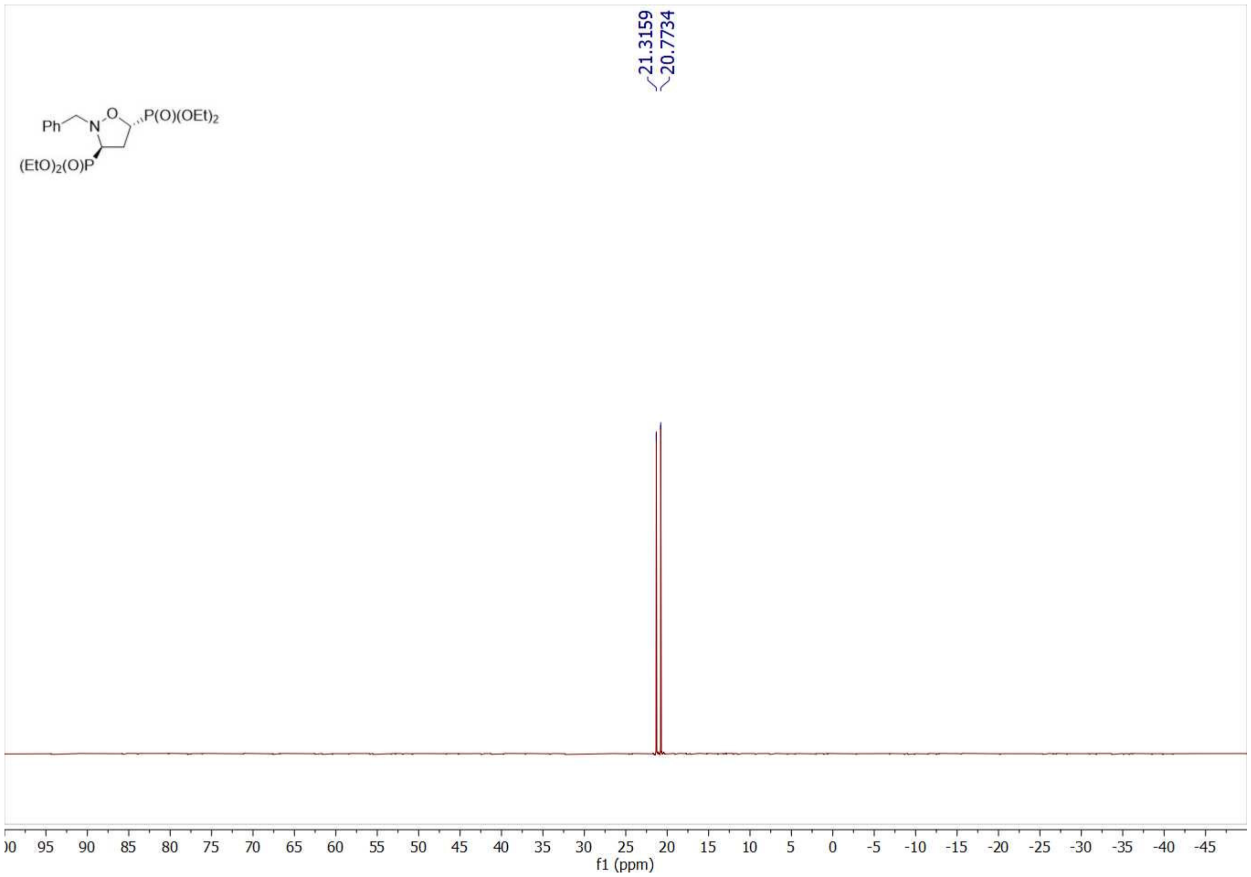

Figure S22: ^{31}P NMR Spectrum for *trans*-**14** in $CDCl_3$

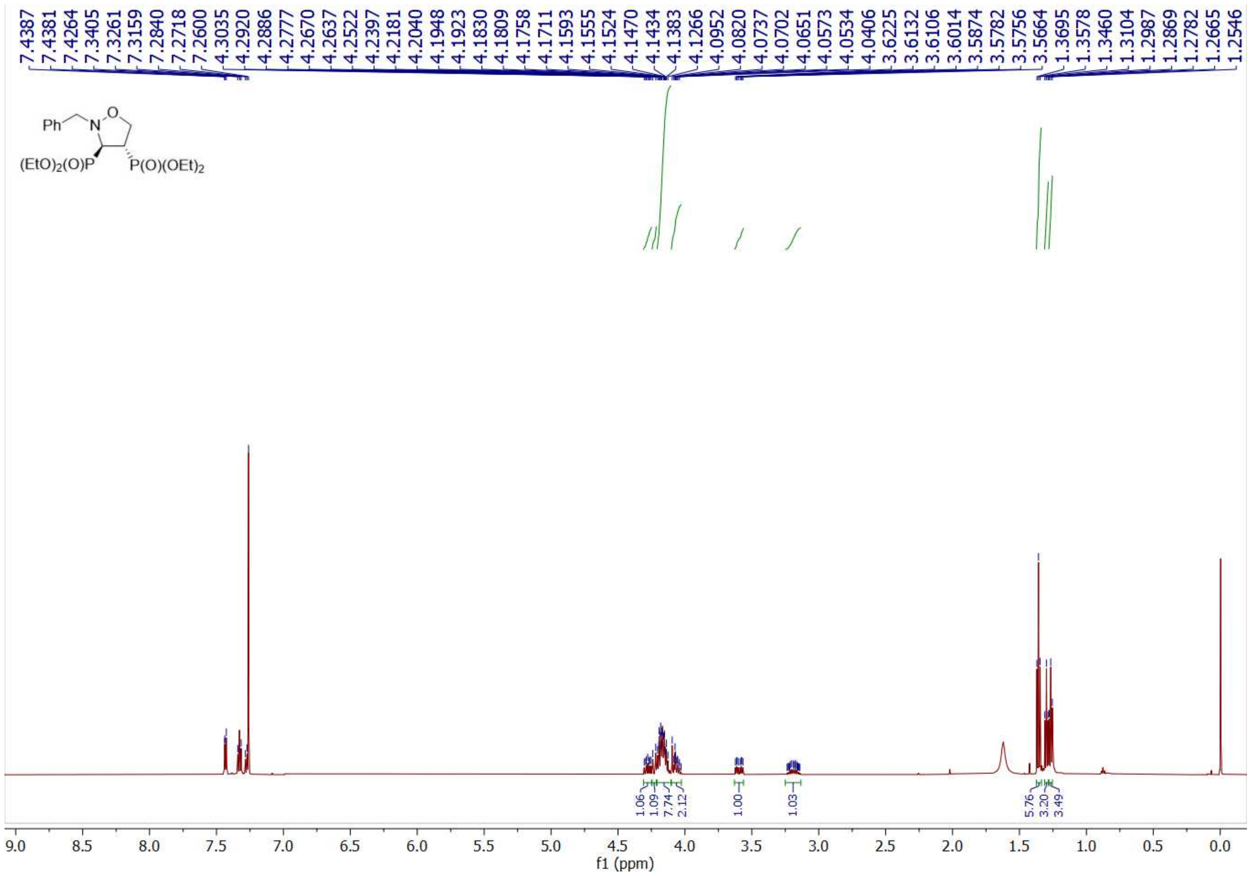

Figure S23: ^1H NMR Spectrum for *trans*-**15** in $CDCl_3$

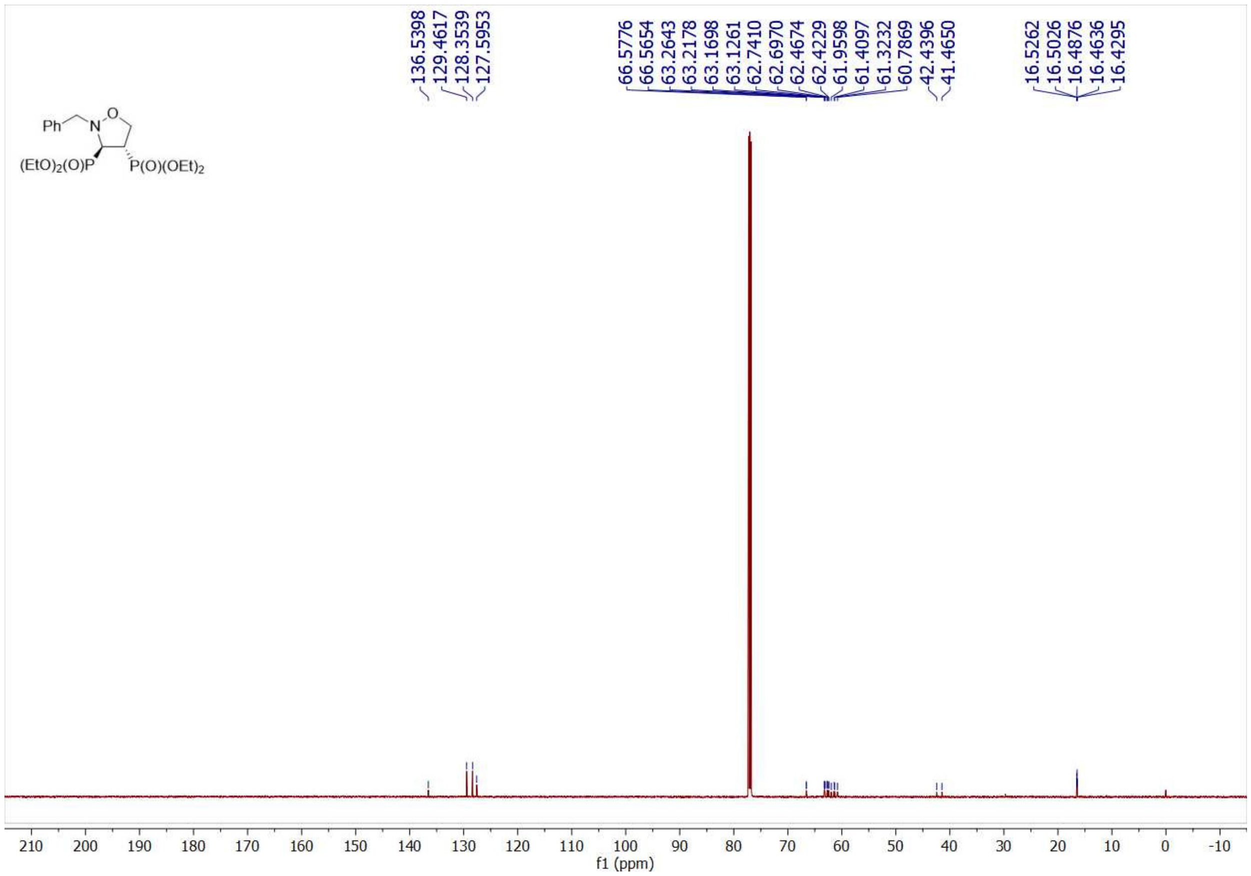

Figure S24: ^{13}C NMR Spectrum for *trans*-**15** in $CDCl_3$

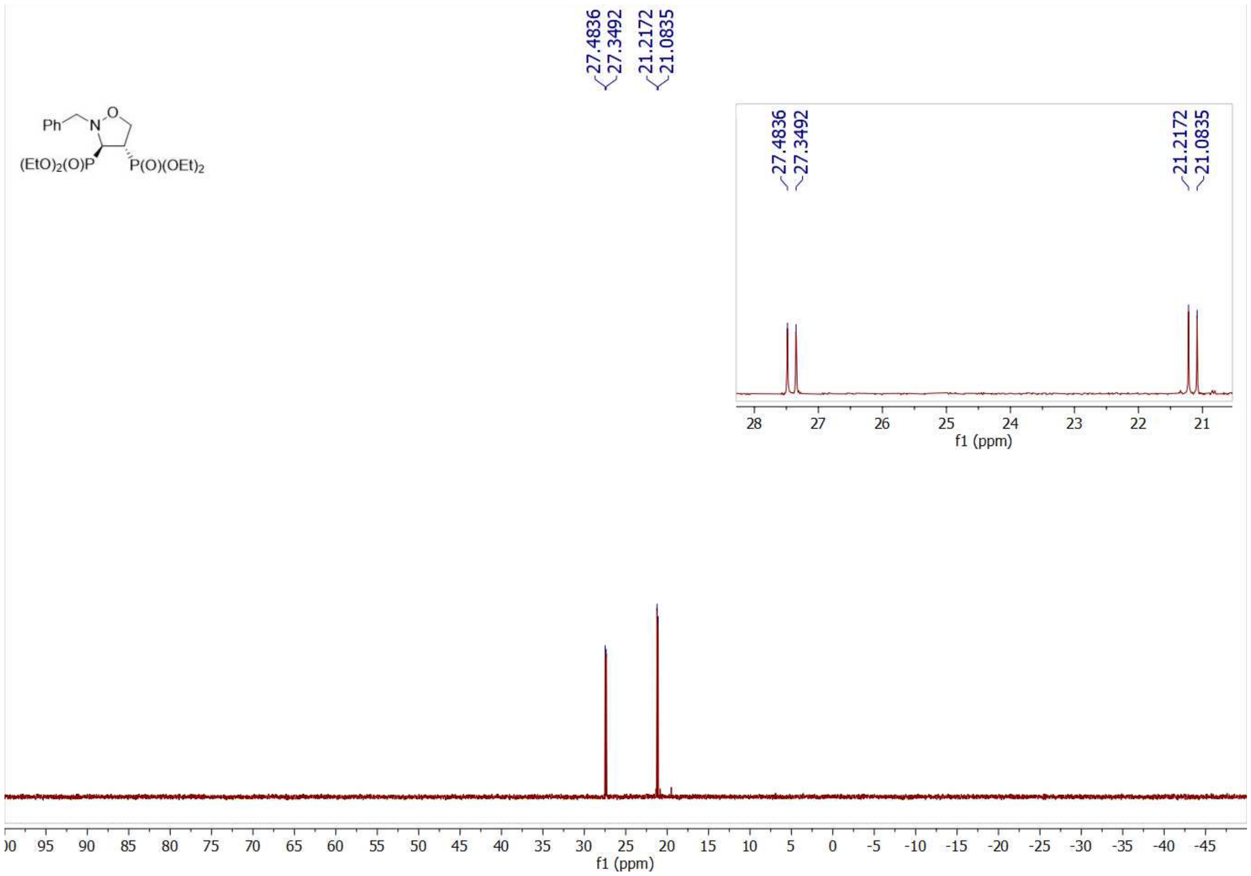

Figure S25: ${}^{31}P$ NMR Spectrum for *trans*-**15** in $CDCl_3$

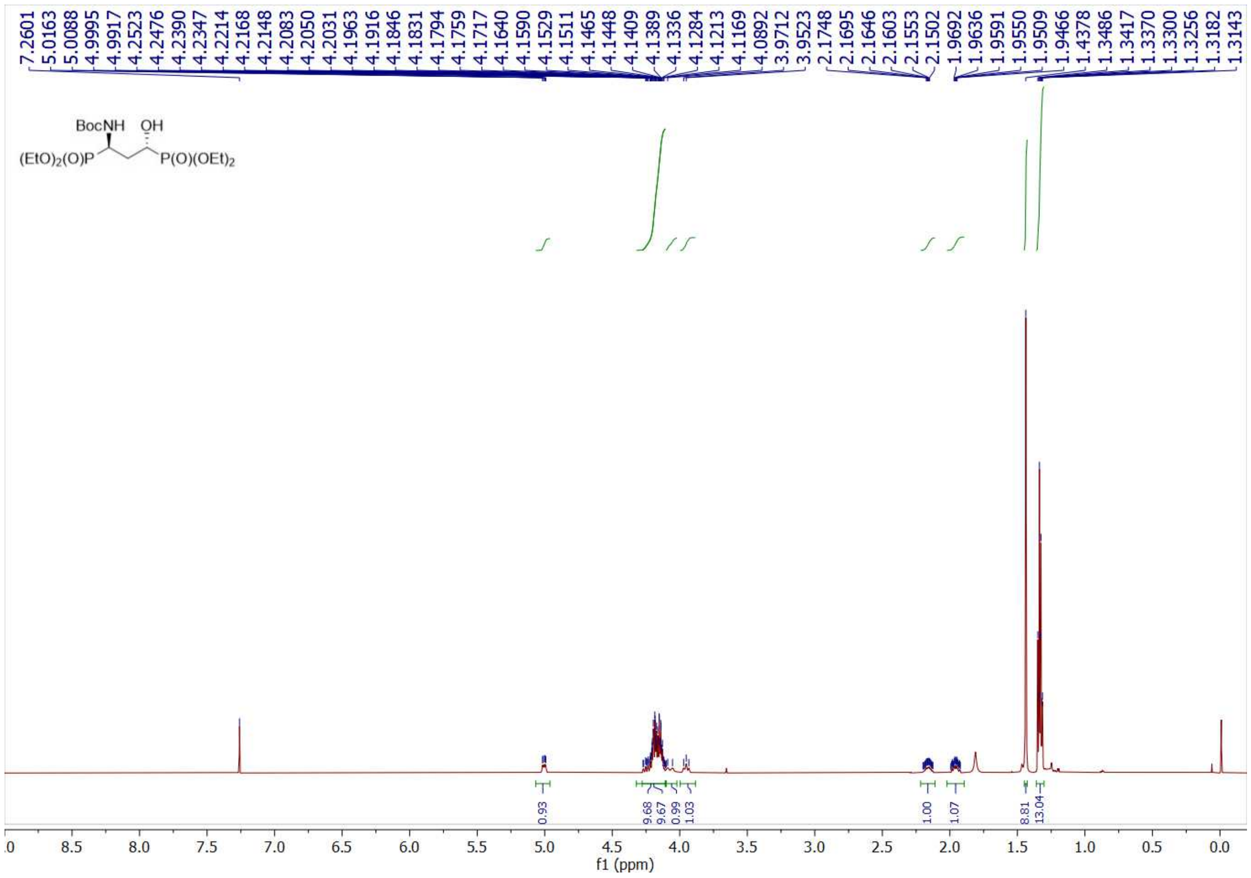

Figure S26: ${}^{1}H$ NMR Spectrum for (1*R*/*S*,3*R*/*S*)-***6*** [*anti*-***6***] in $CDCl_3$

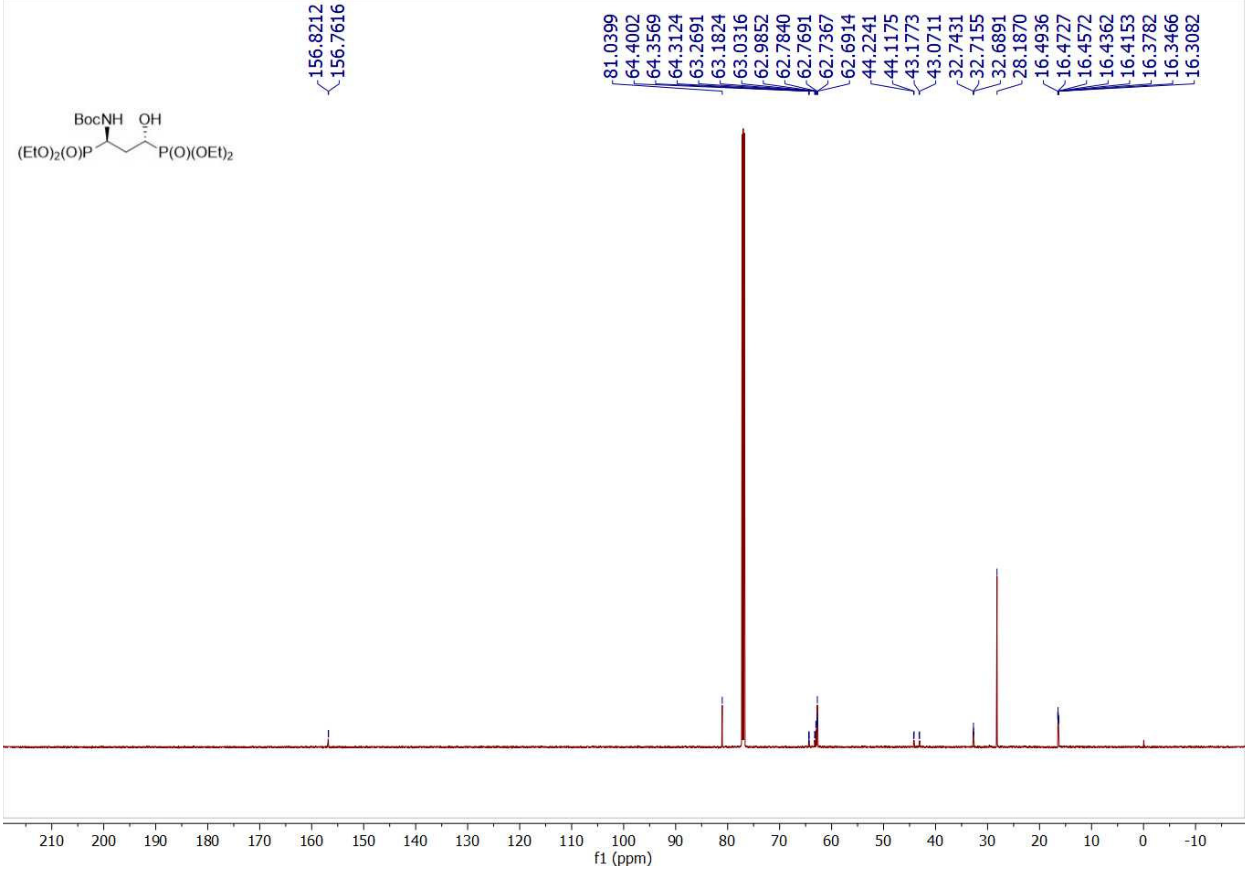

Figure S27: ^{13}C NMR Spectrum for (1*R*/*S*,3*R*/*S*)-***6*** [*anti*-***6***] in $CDCl_3$

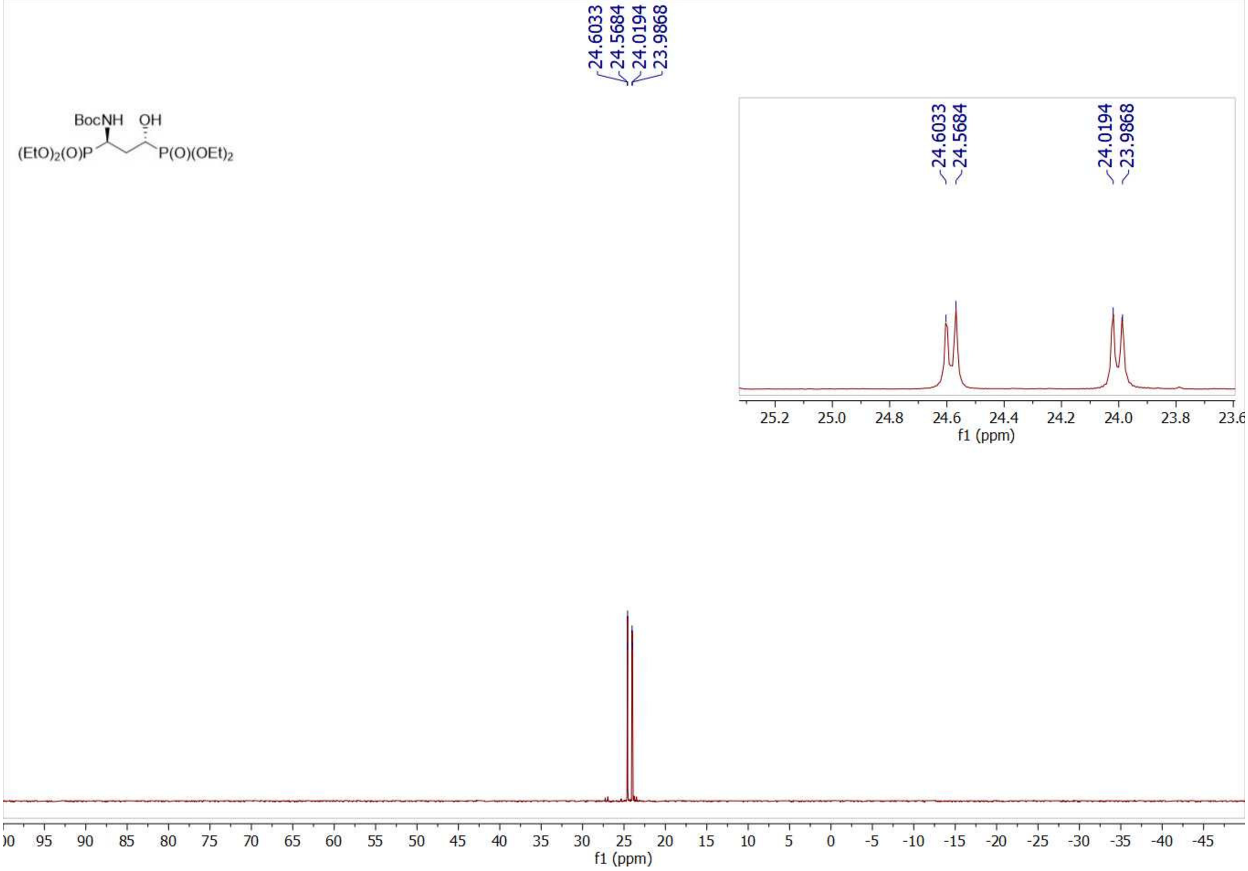

Figure S28: ^{31}P NMR Spectrum for (1*R*/*S*,3*R*/*S*)-***6*** [*anti*-***6***] in $CDCl_3$

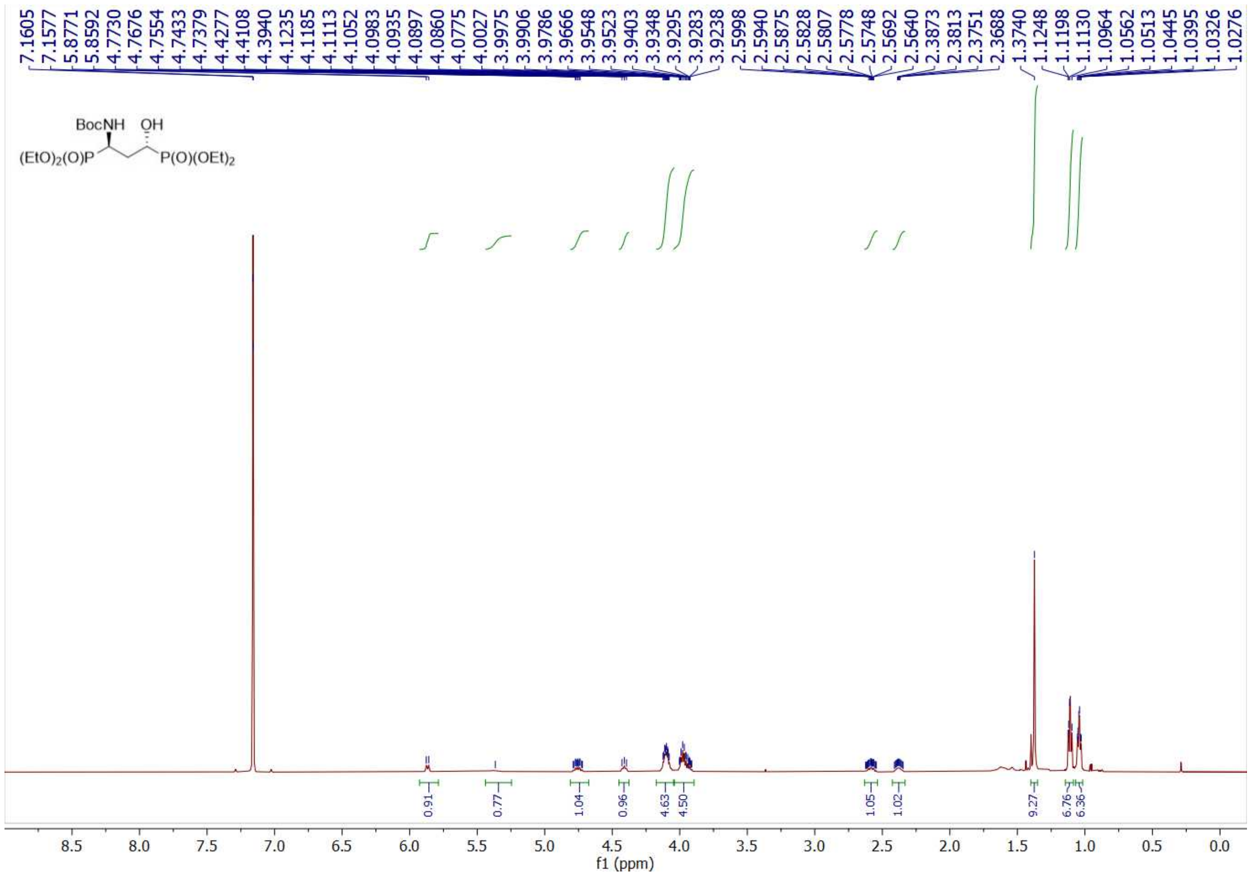

Figure S29: ^{1}H NMR Spectrum for (1*R*/*S*,3*R*/*S*)-***6*** [*anti*-***6***] in C_6D_6

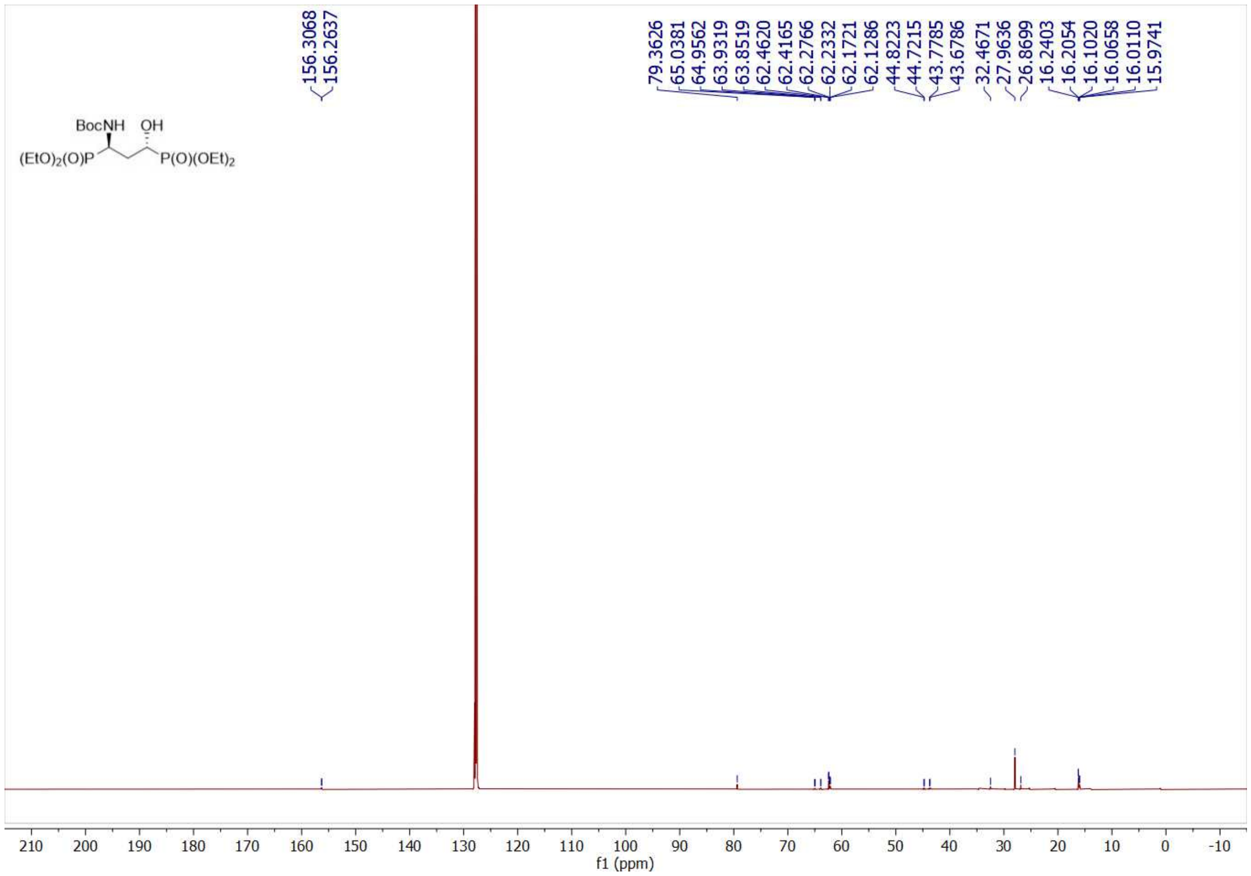

Figure S30: ^{13}C NMR Spectrum for (1*R*/*S*,3*R*/*S*)-***6*** [*anti*-***6***] in C_6D_6

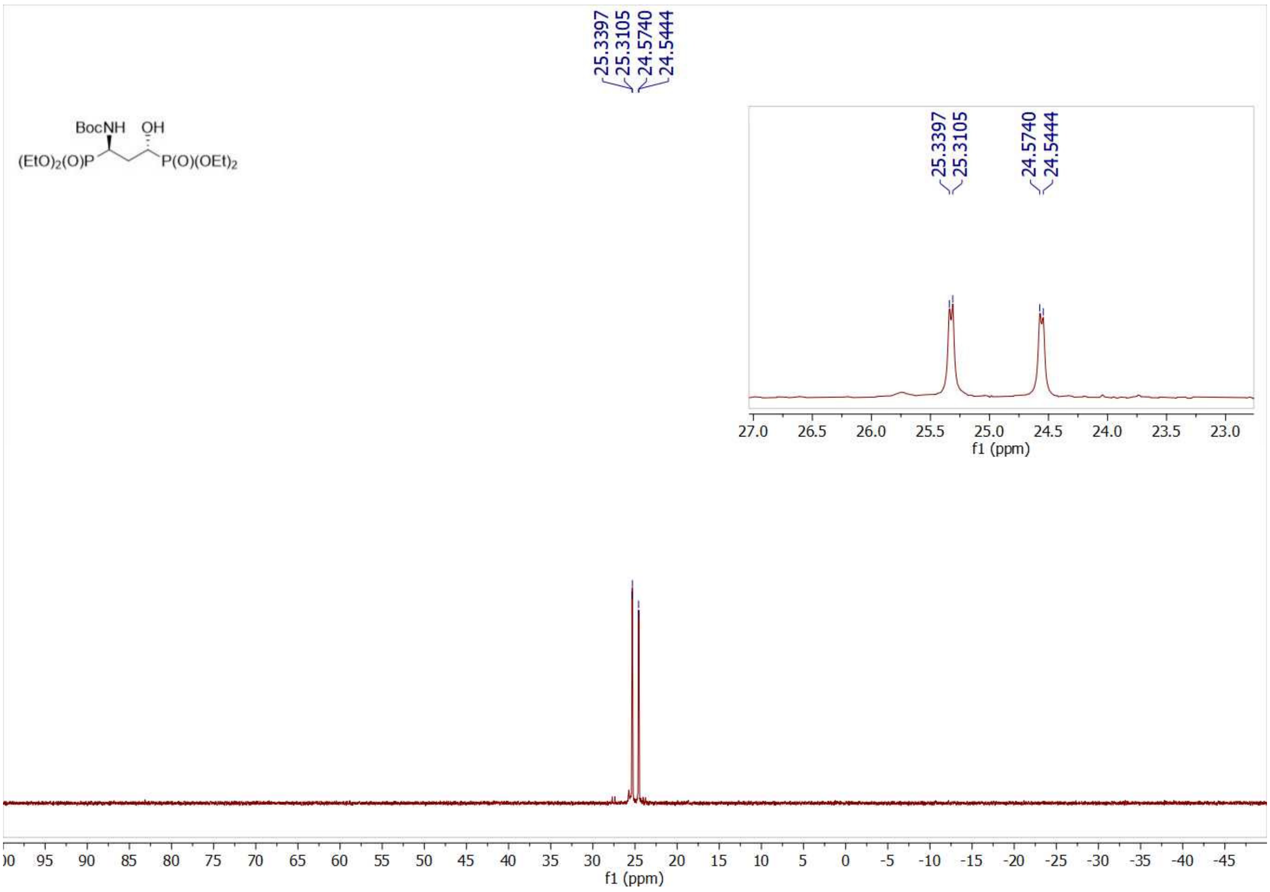

Figure S31: ³¹P NMR Spectrum for (1*R*/*S*,3*R*/*S*)-***6*** [*anti*-***6***] in C_6D_6

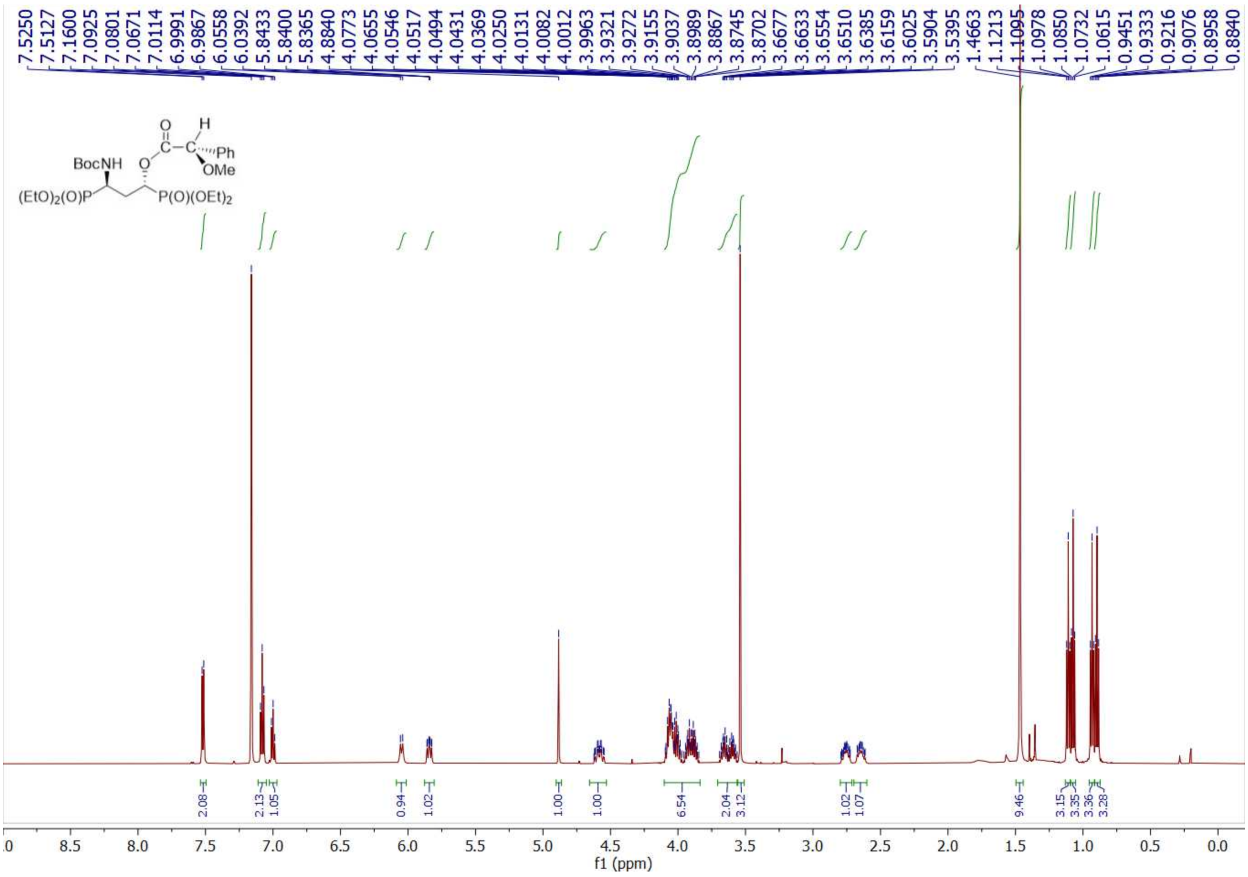

Figure S32: ¹H NMR Spectrum for (1*R*,3*R*,1′*S*)-**16** in C_6D_6

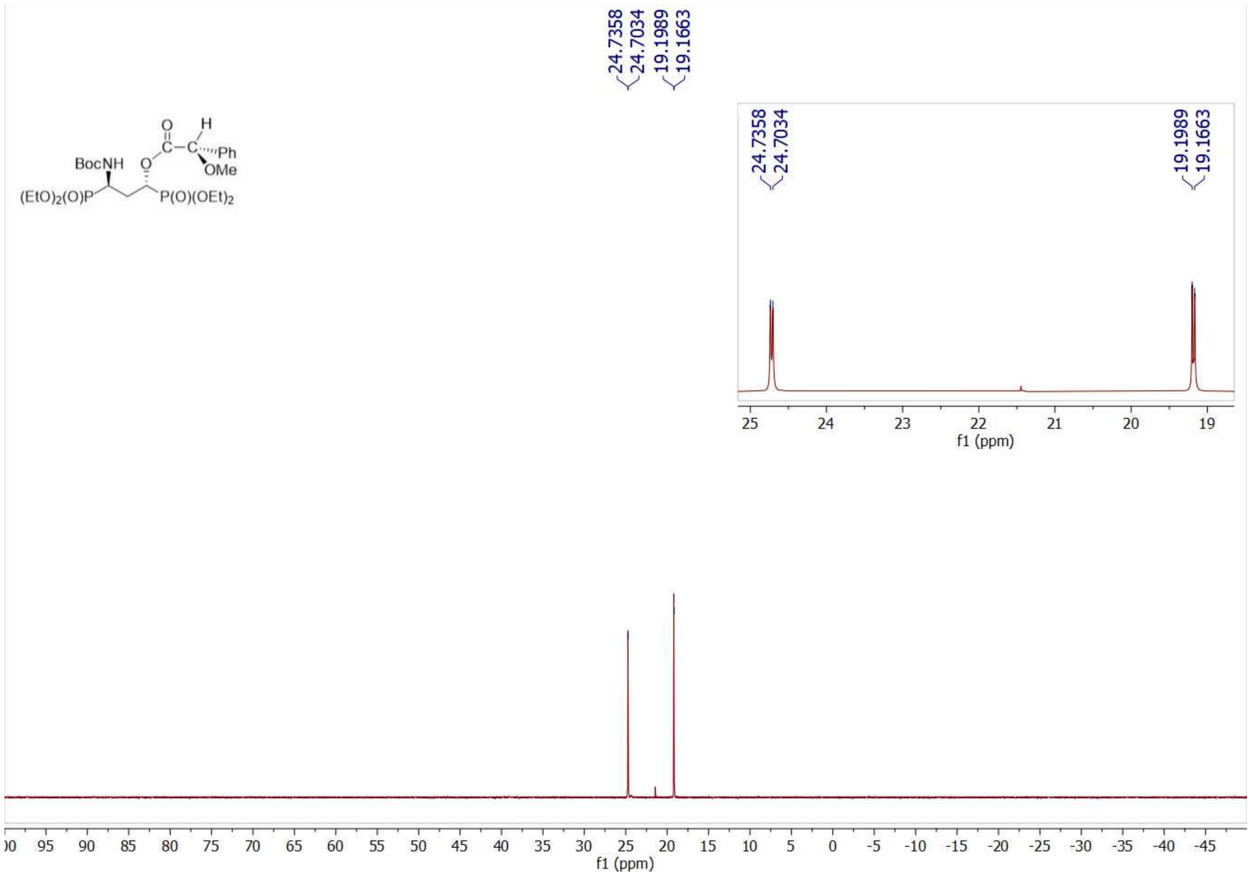

Figure S33: ^{31}P NMR Spectrum for (1*R*,3*R*,1′*S*)-**16** in C_6D_6

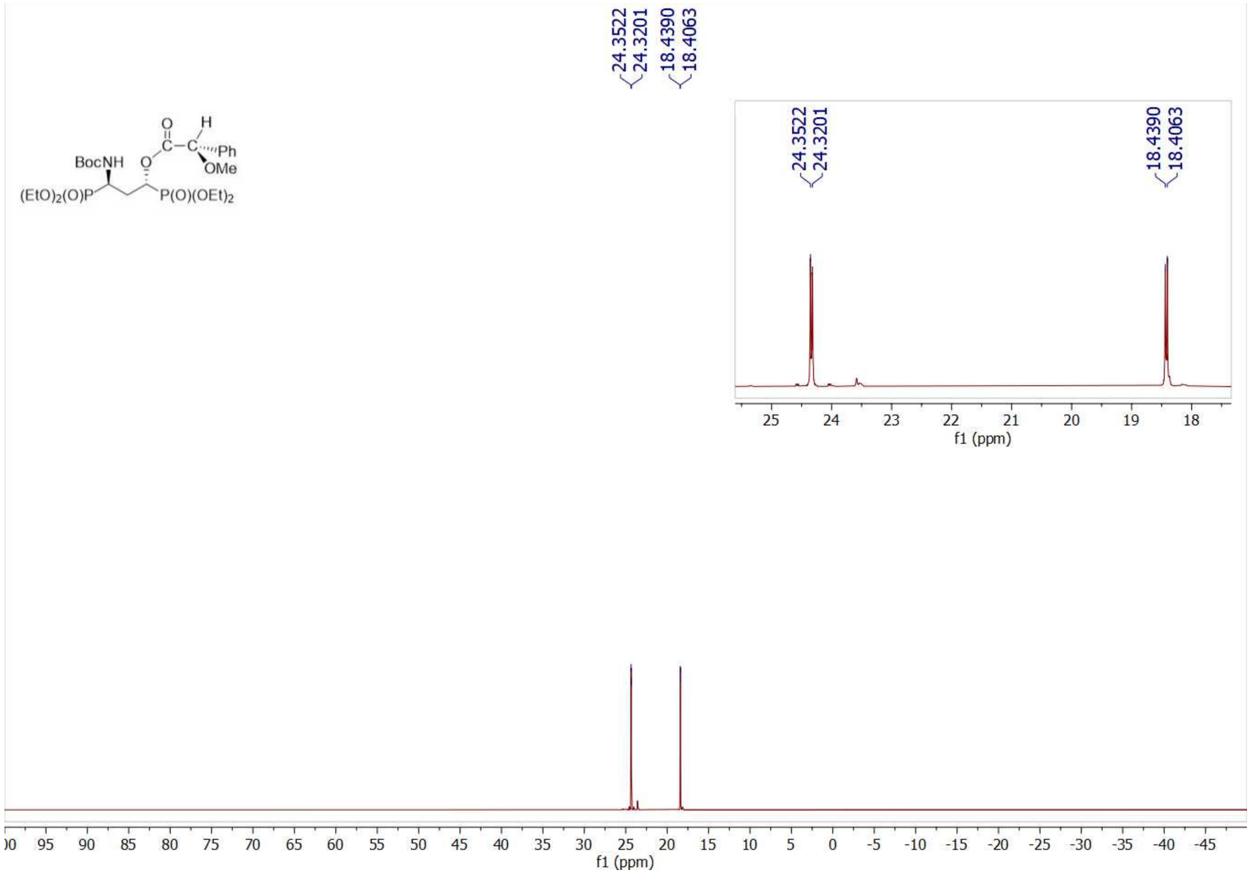

Figure S34: ^{31}P NMR Spectrum for (1*R*,3*R*,1′*S*)-**16** in $CDCl_3$

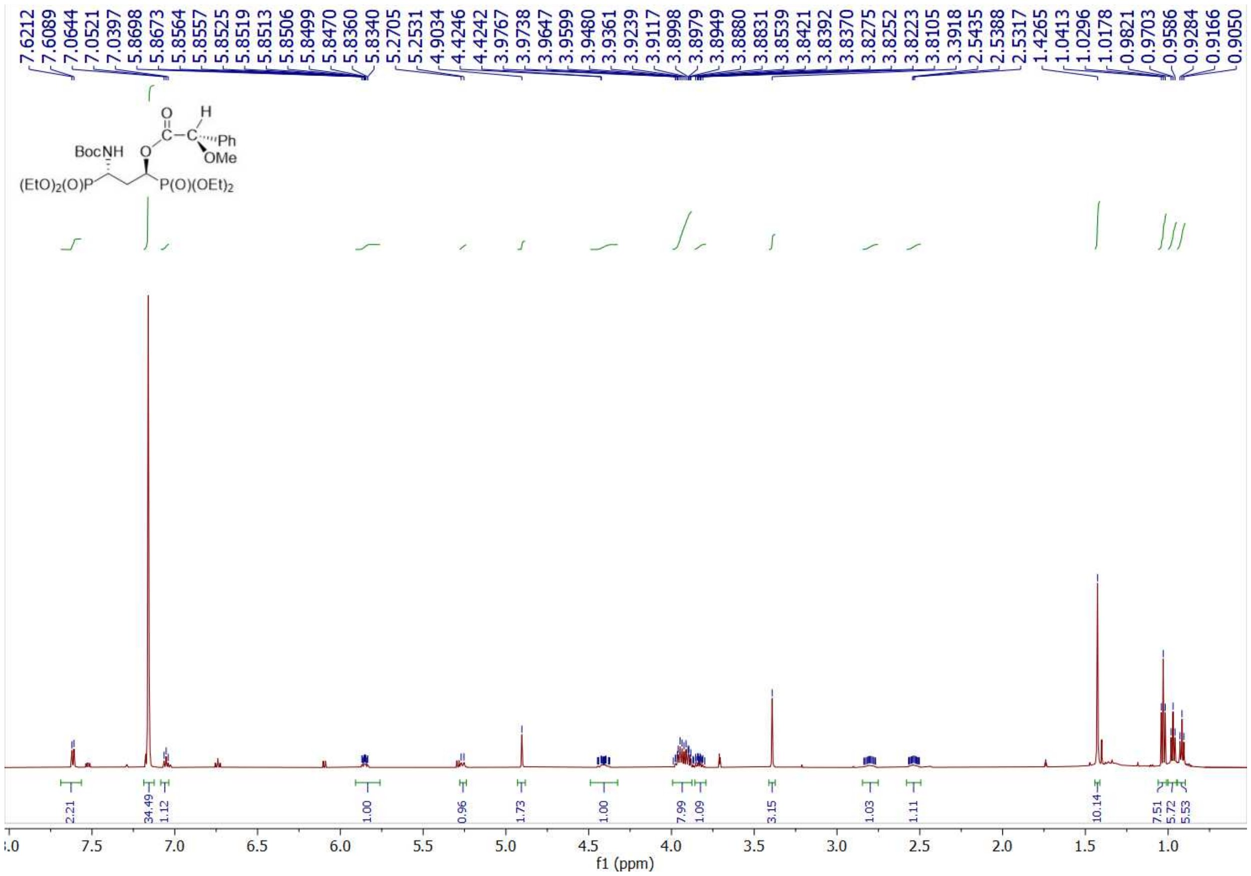

Figure S35: ^{1}H NMR Spectrum for (1*S*,3*S*,1′*S*)-**16** in C_6D_6

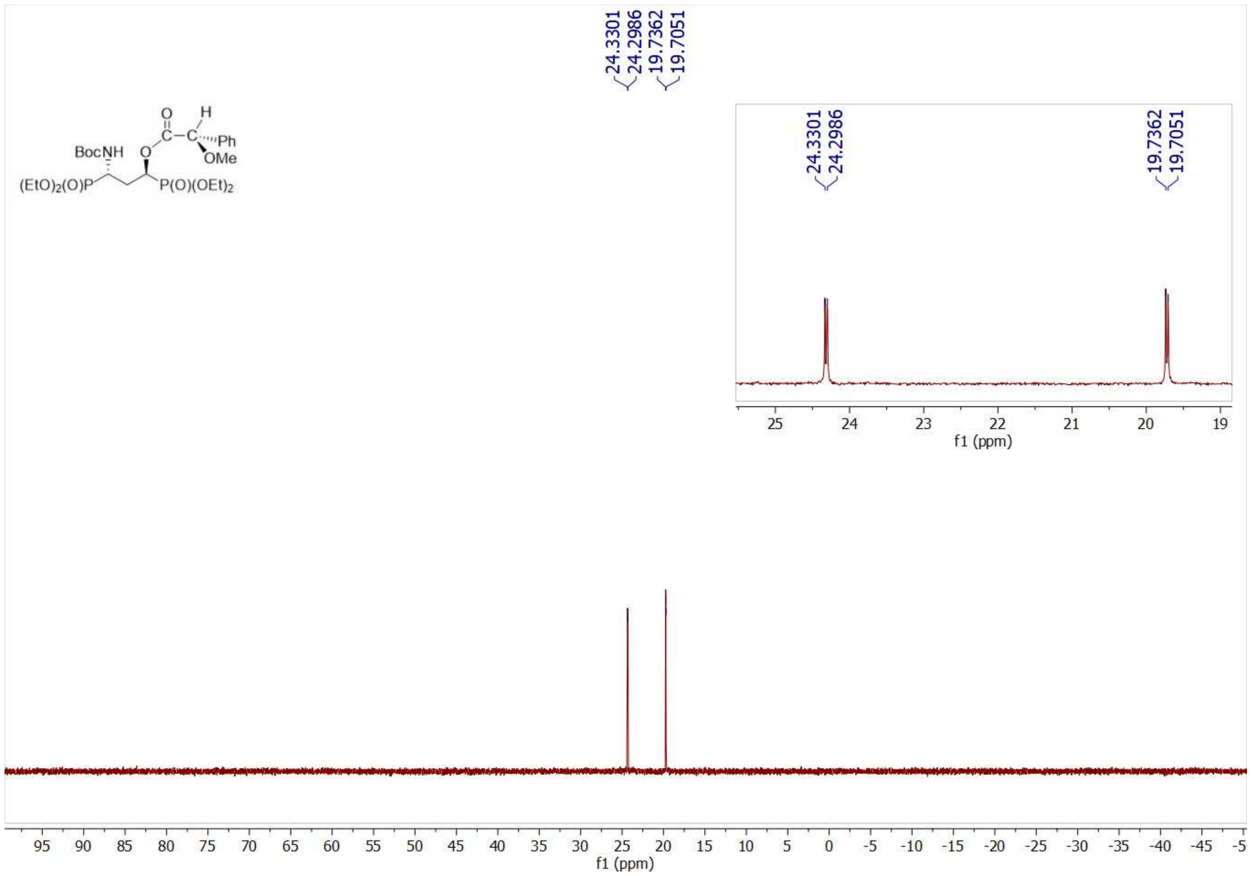

Figure S36: ^{31}P NMR Spectrum for (1*S*,3*S*,1′*S*)-**16** in C_6D_6

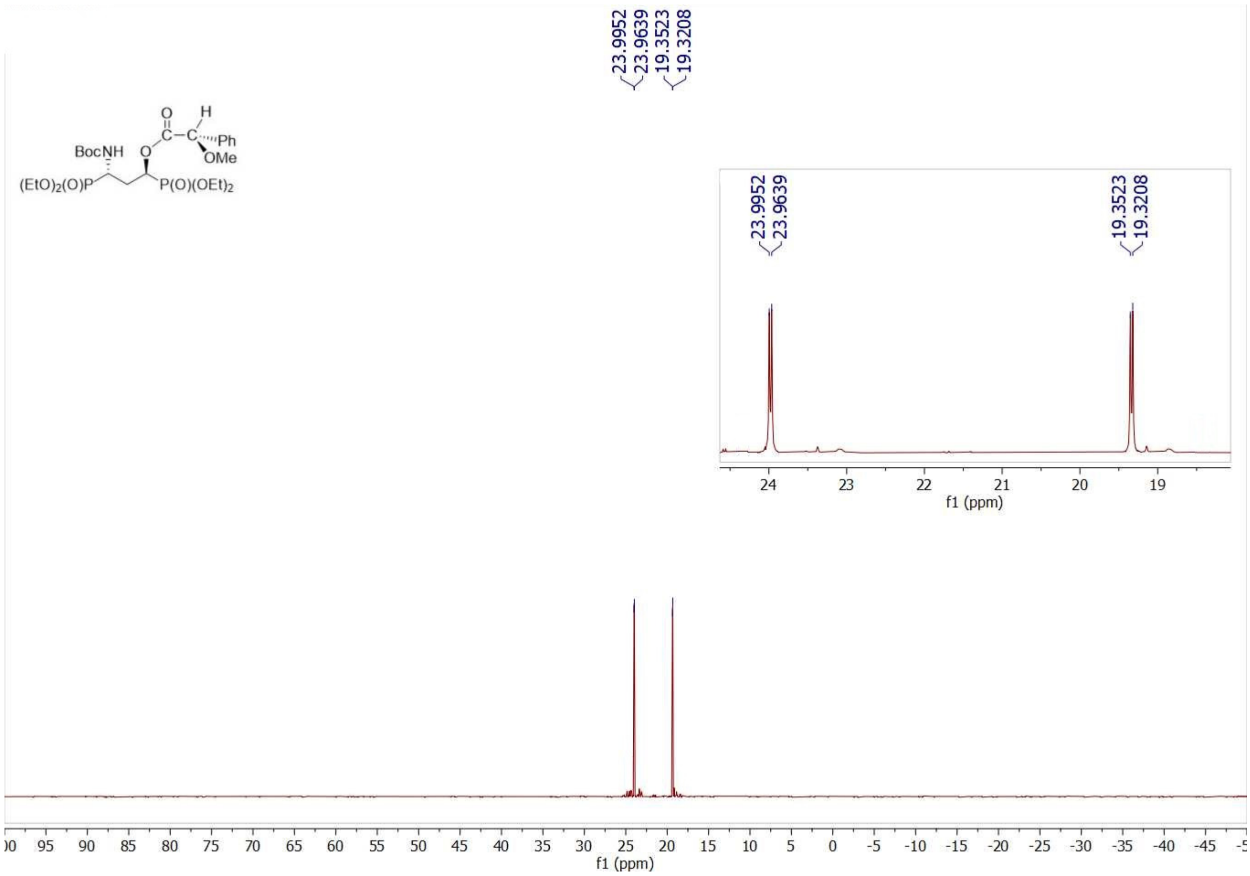

Figure S37: ^{31}P NMR Spectrum for (1*S*,3*S*,1′*S*)-**16** in $CDCl_3$

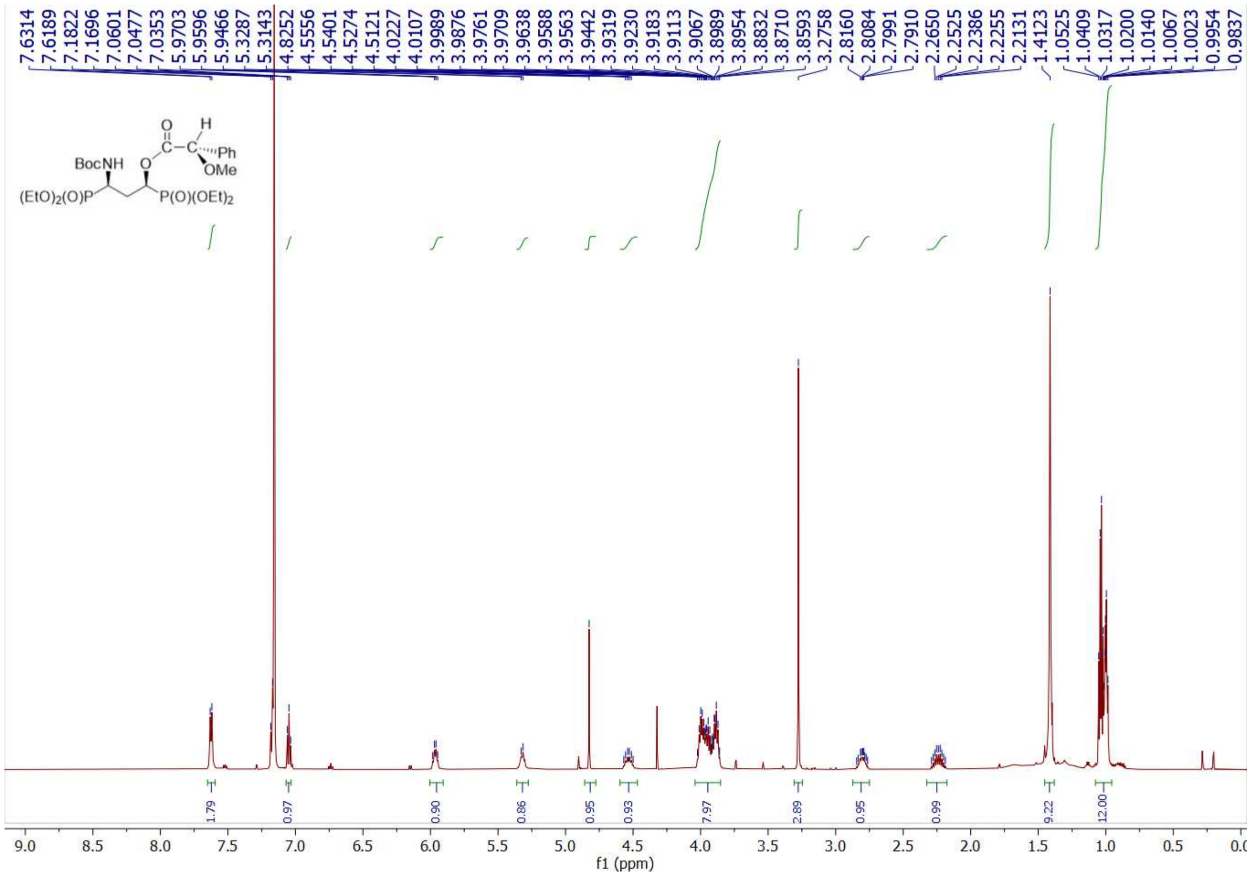

Figure S38: ^{1}H NMR Spectrum for (1*R*,3*S*,1′*S*)-**16** in C_6D_6

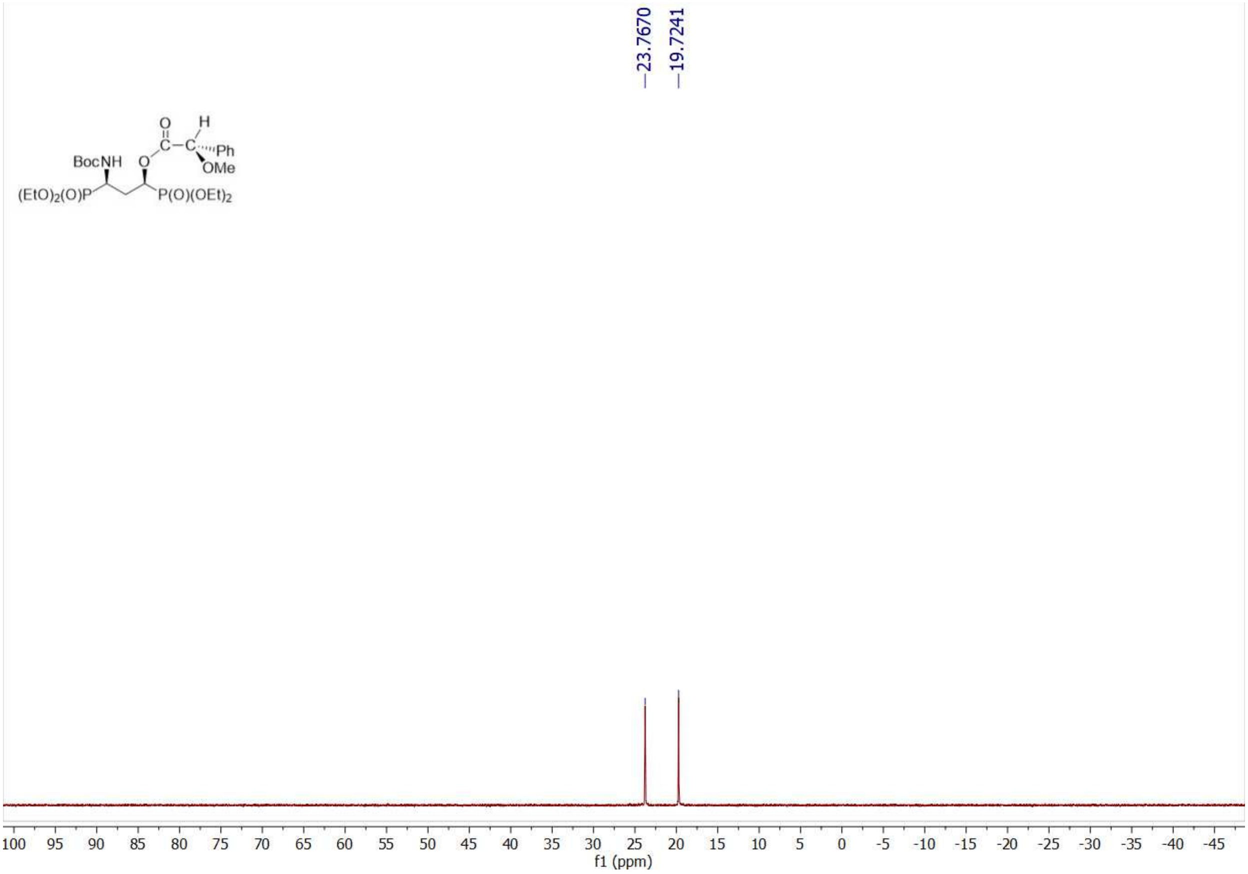

Figure S39: ^{31}P NMR Spectrum for (1*R*,3*S*,1′*S*)-**16** in C_6D_6

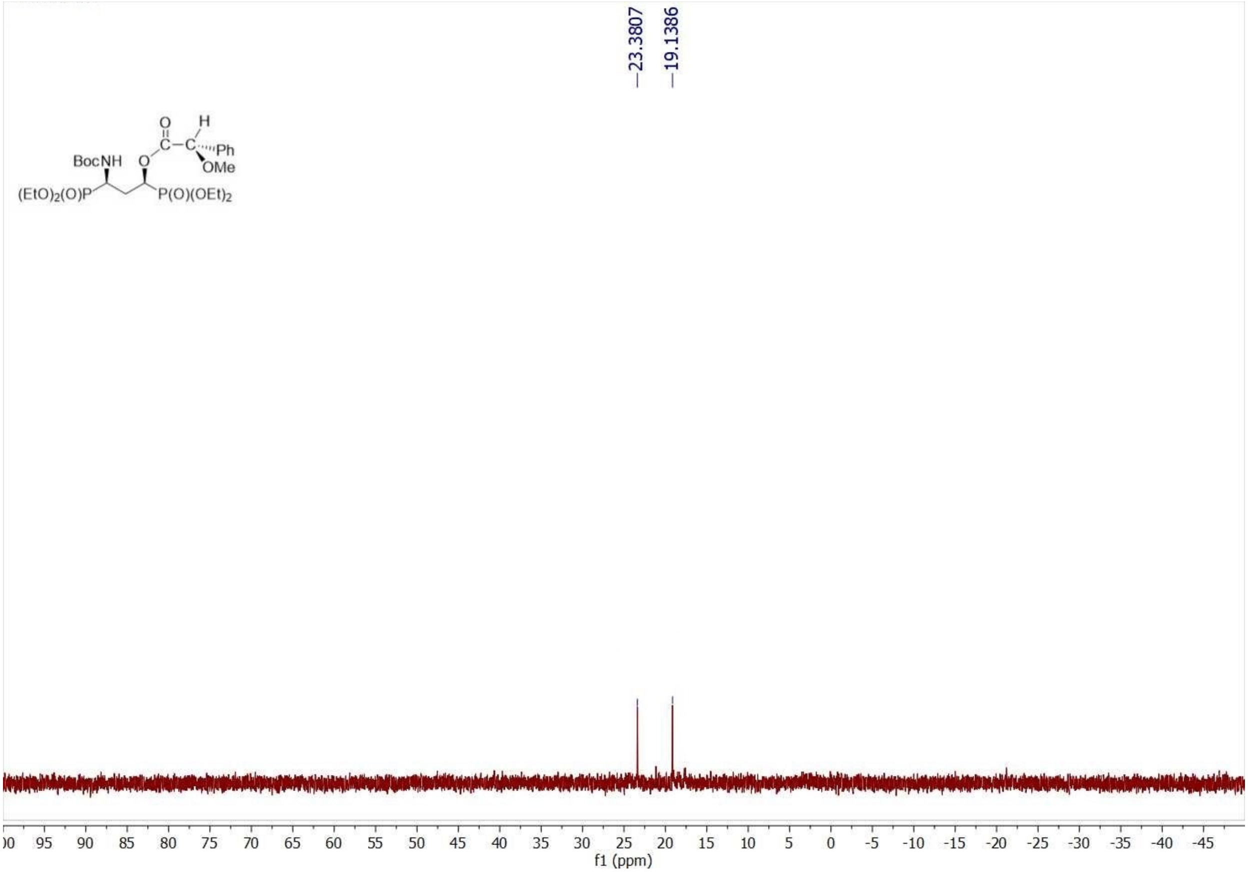

Figure S40: ^{31}P NMR Spectrum for (1*R*,3*S*,1′*S*)-**16** in $CDCl_3$

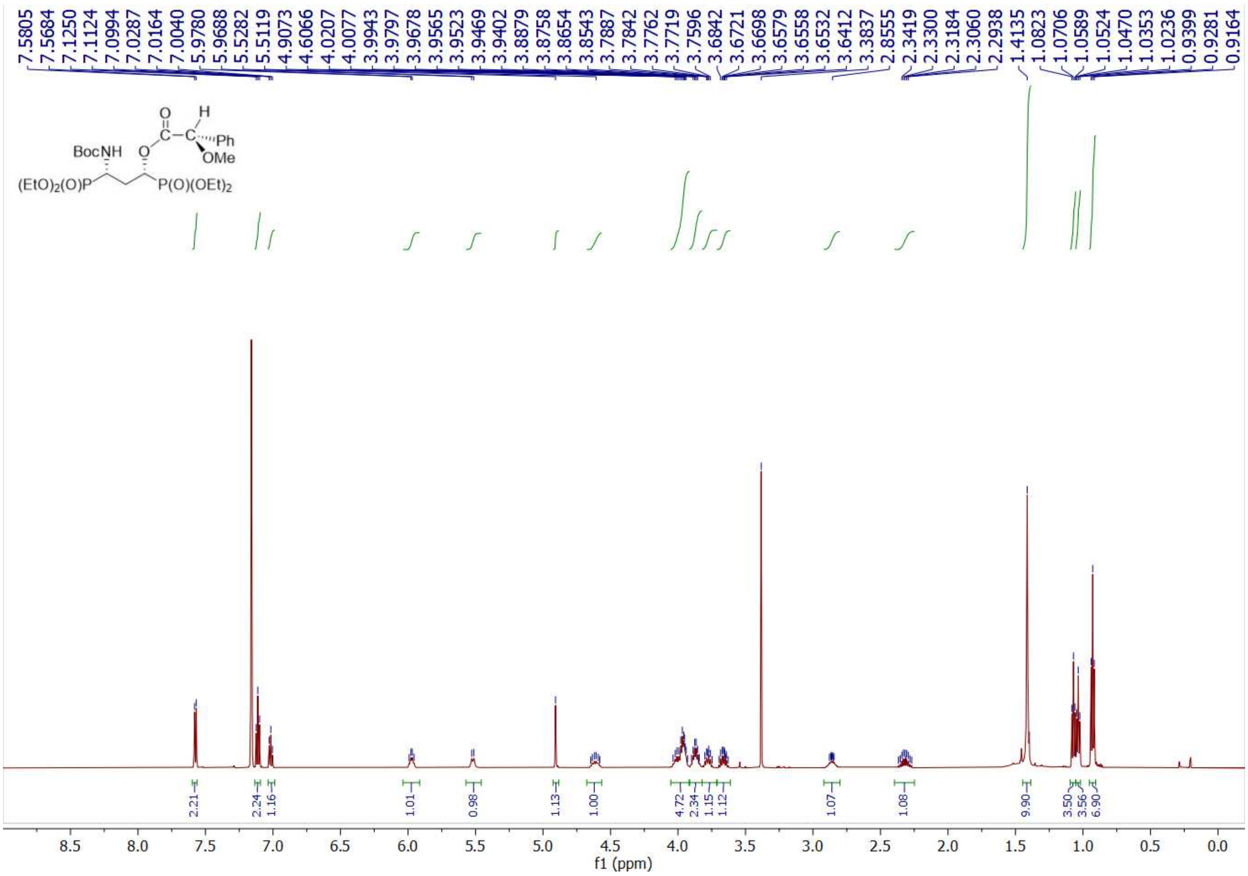

Figure S41: ^{1}H NMR Spectrum for (1*S*,3*R*,1′*S*)-**16** in C_6D_6

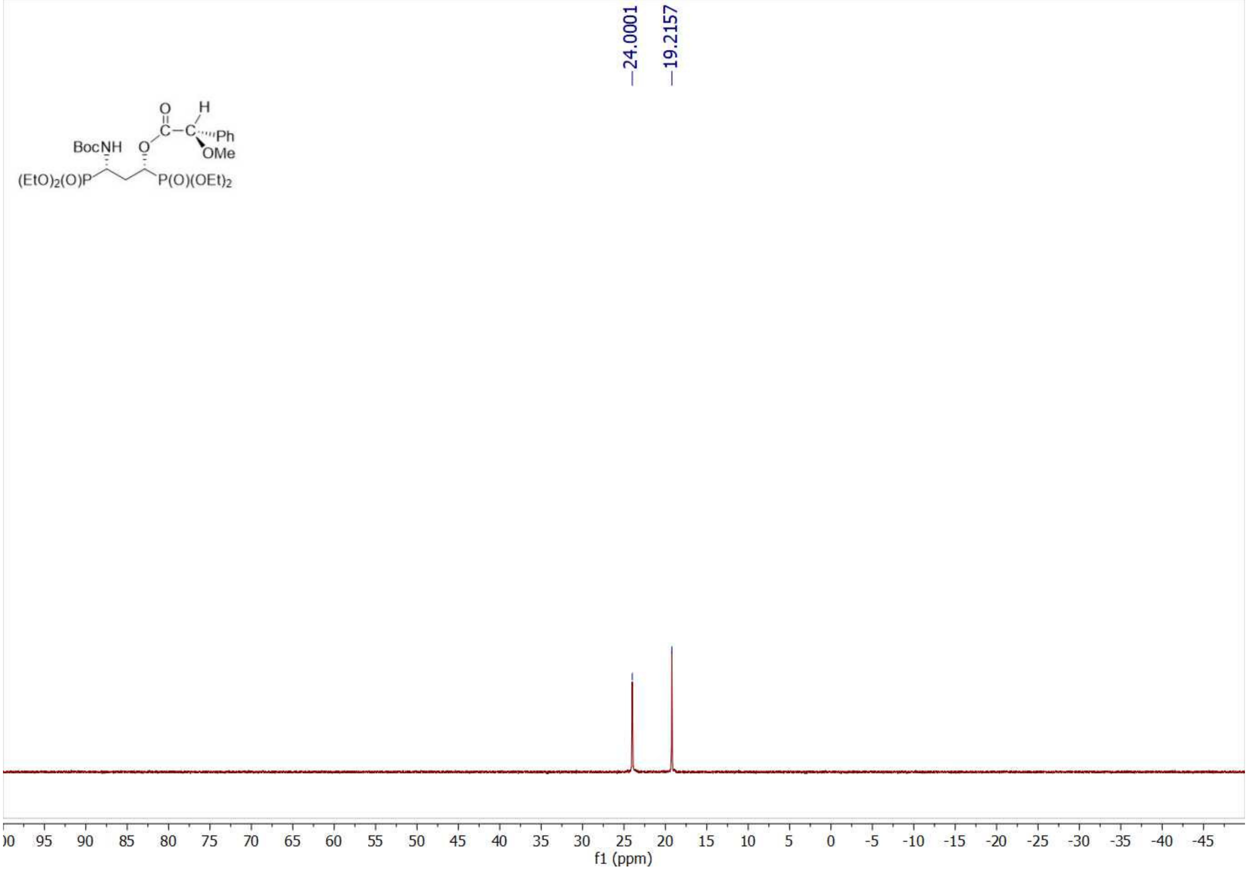

Figure S42: ^{31}P NMR Spectrum for (1*S*,3*R*,1′*S*)-**16** in C_6D_6

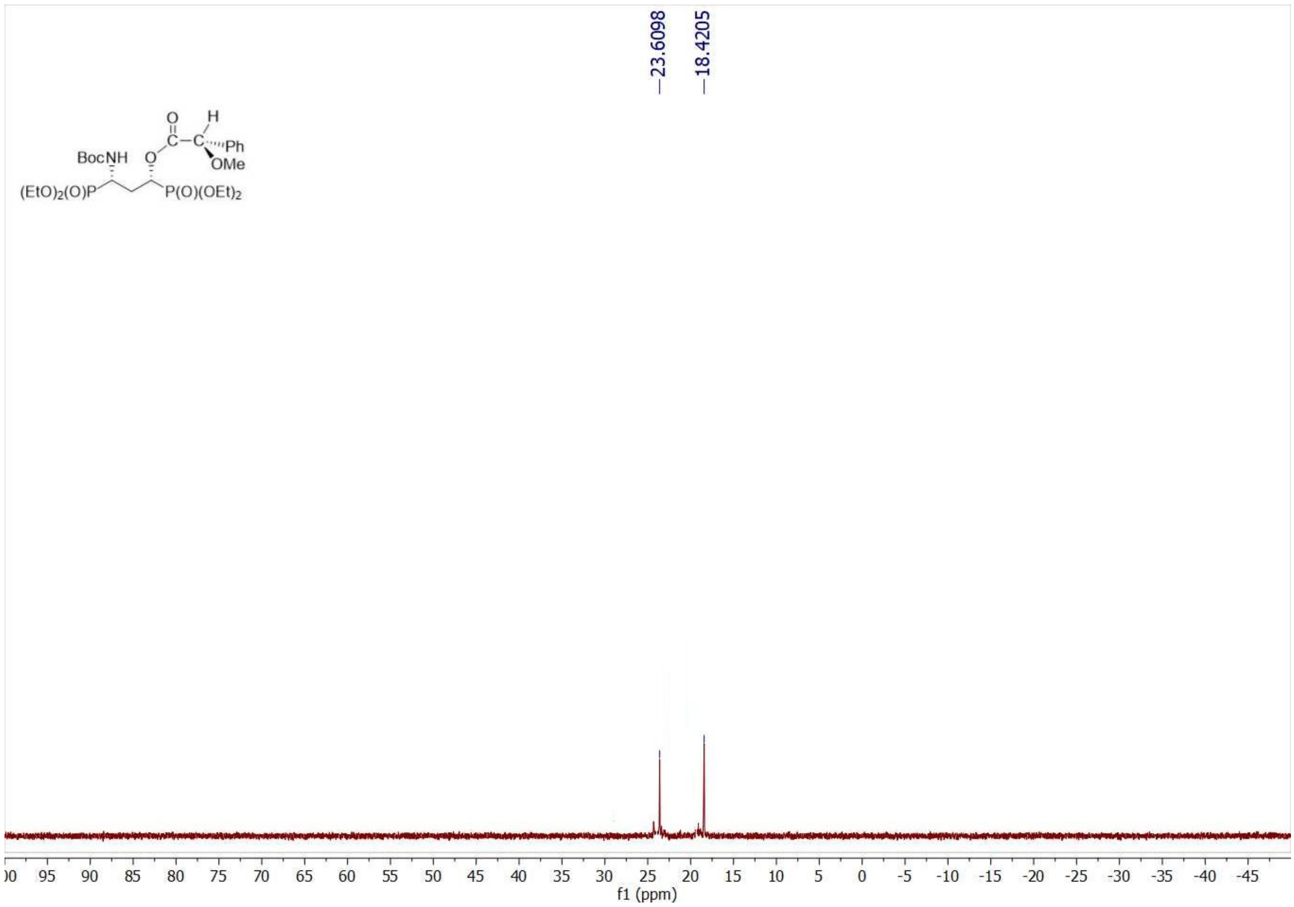

Figure S43: ^{31}P NMR Spectrum for (1*S*,3*R*,1′*S*)-**16** in $CDCl_3$

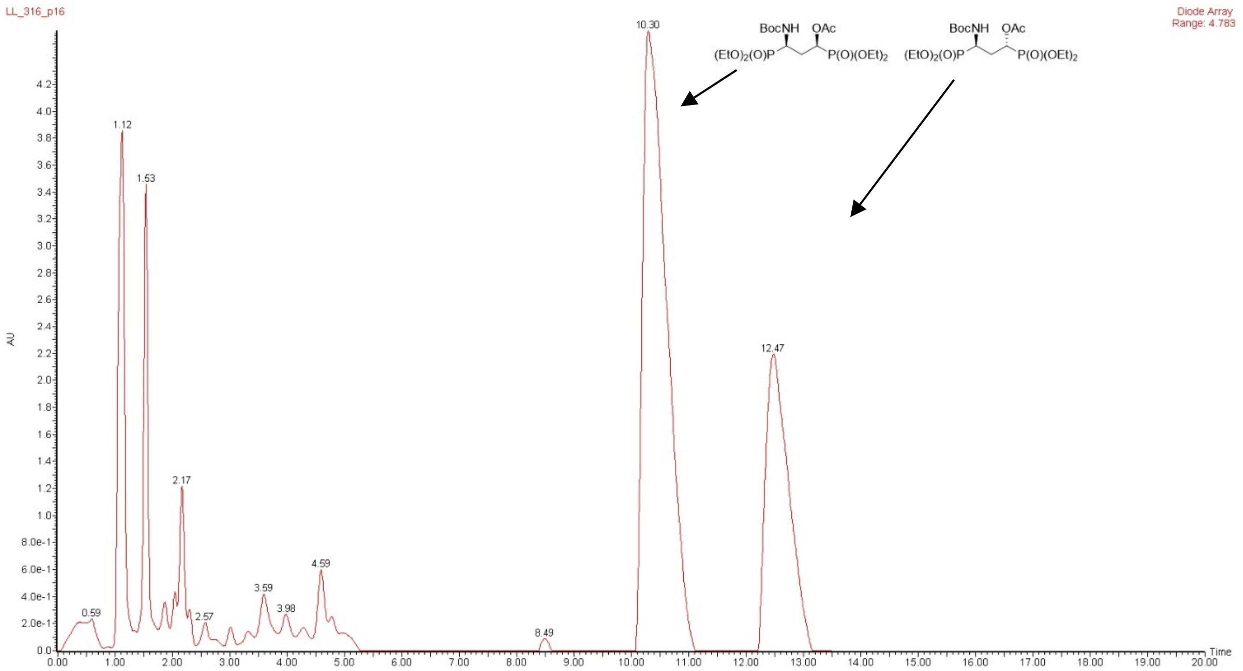

Figure S44: Separation of (1*R*,3*S*)-**11** and (1*R*,3R)-**11** by preparative HPLC

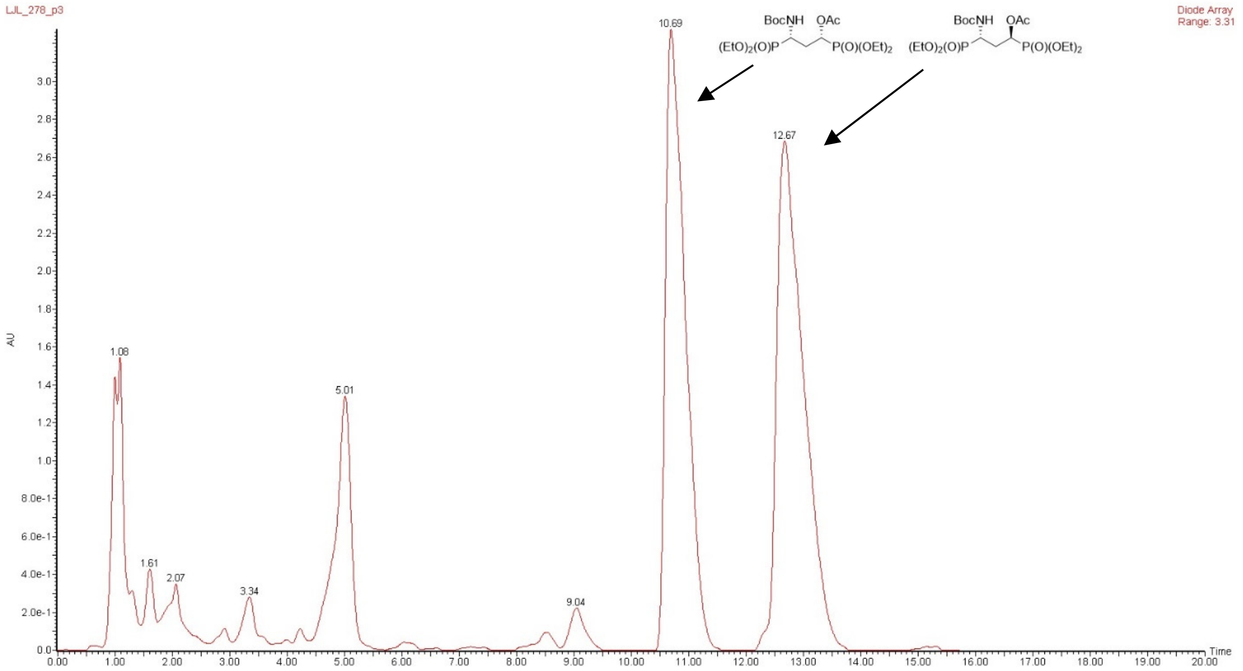

Figure S45: Separation of (1*S*,3*R*)-**11** and (1*S*,3*S*)-**11** by preparative HPLC

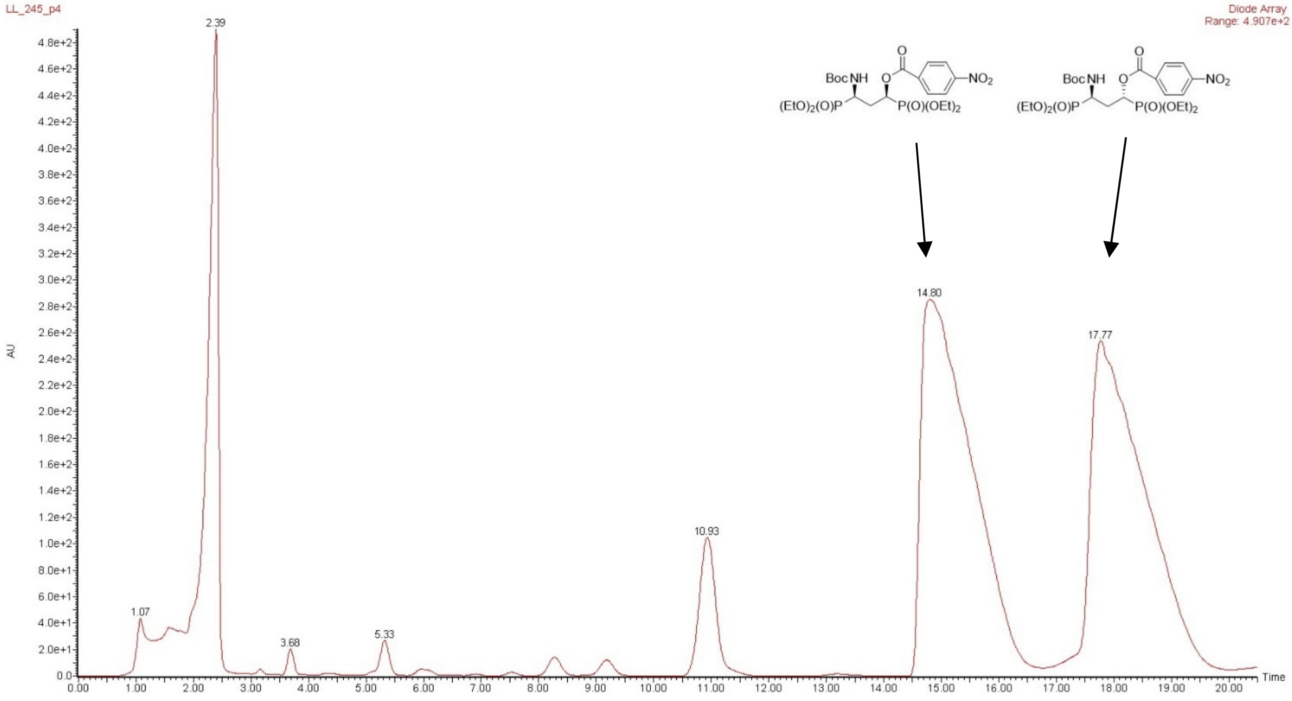

Figure S46: Separation of (1*R*,3*S*)-**12** and (1*R*,3*R*)-**12** by preparative HPLC

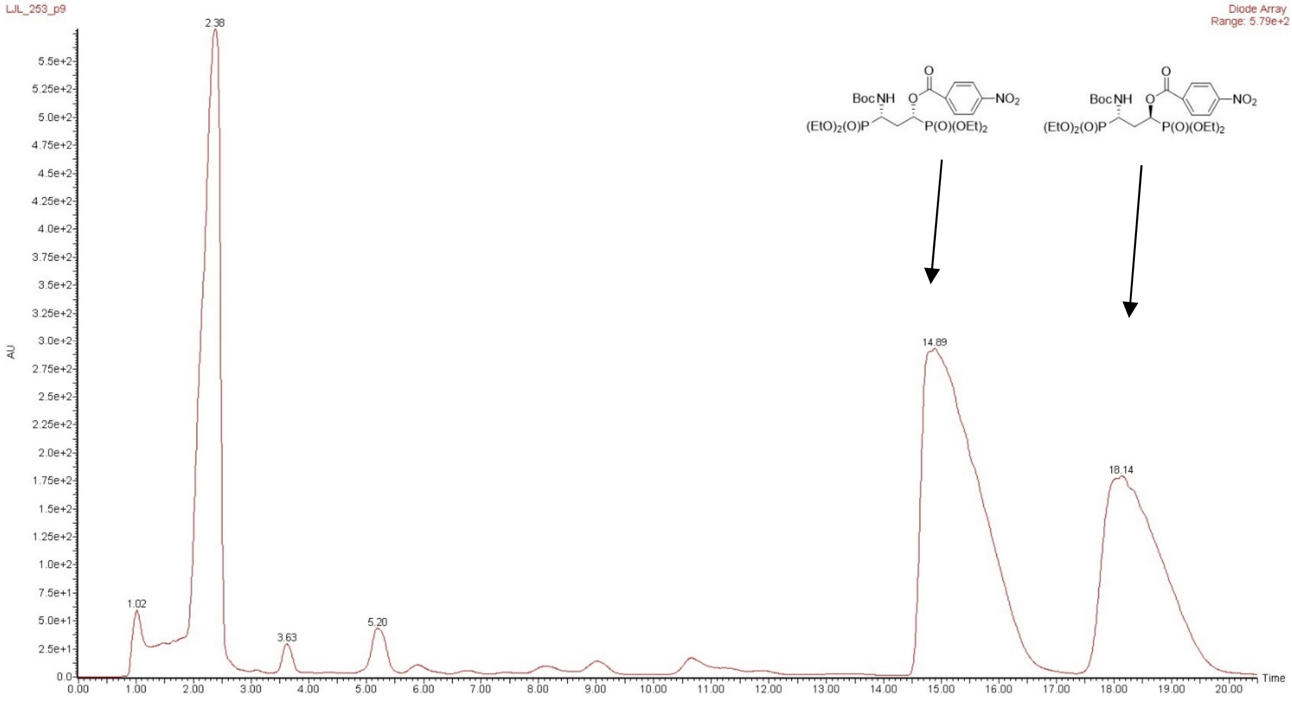

Figure S47: Separation of (1*S*,3*R*)-**12** and (1*S*,3*S*)-**12** by preparative HPLC

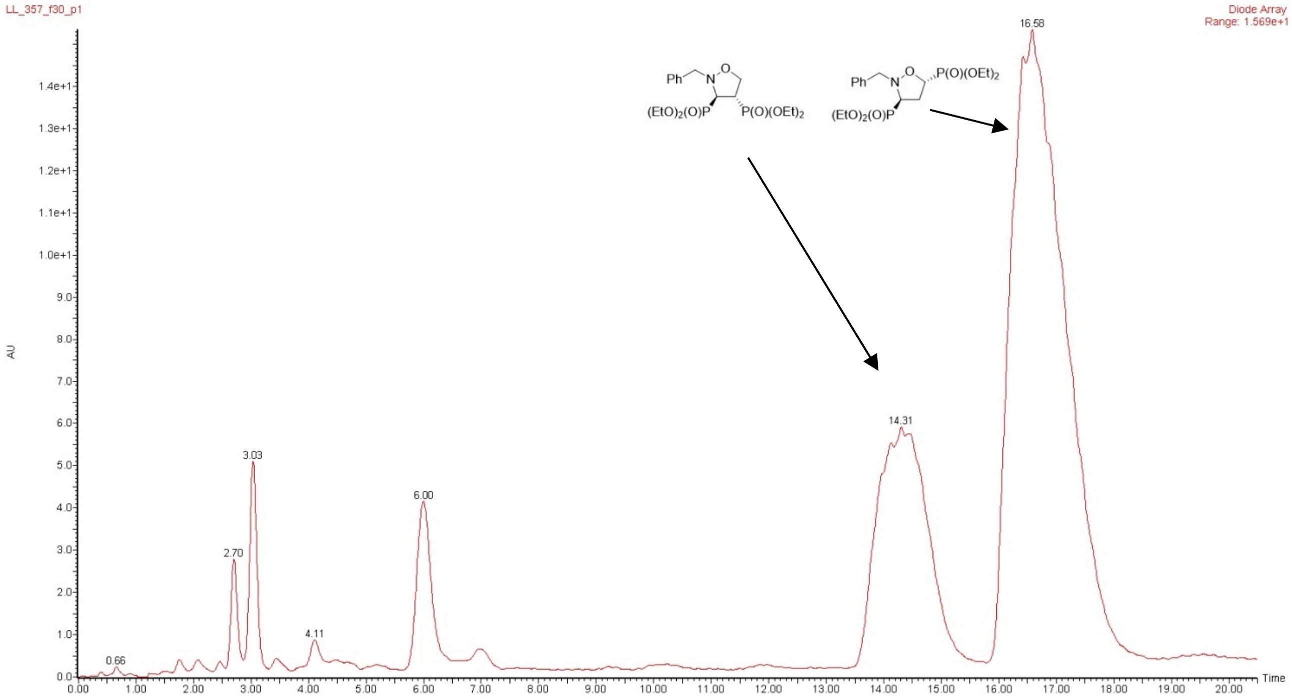

Figure S48: Separation of *trans*-**14** and *trans*-**15** by preparative HPLC

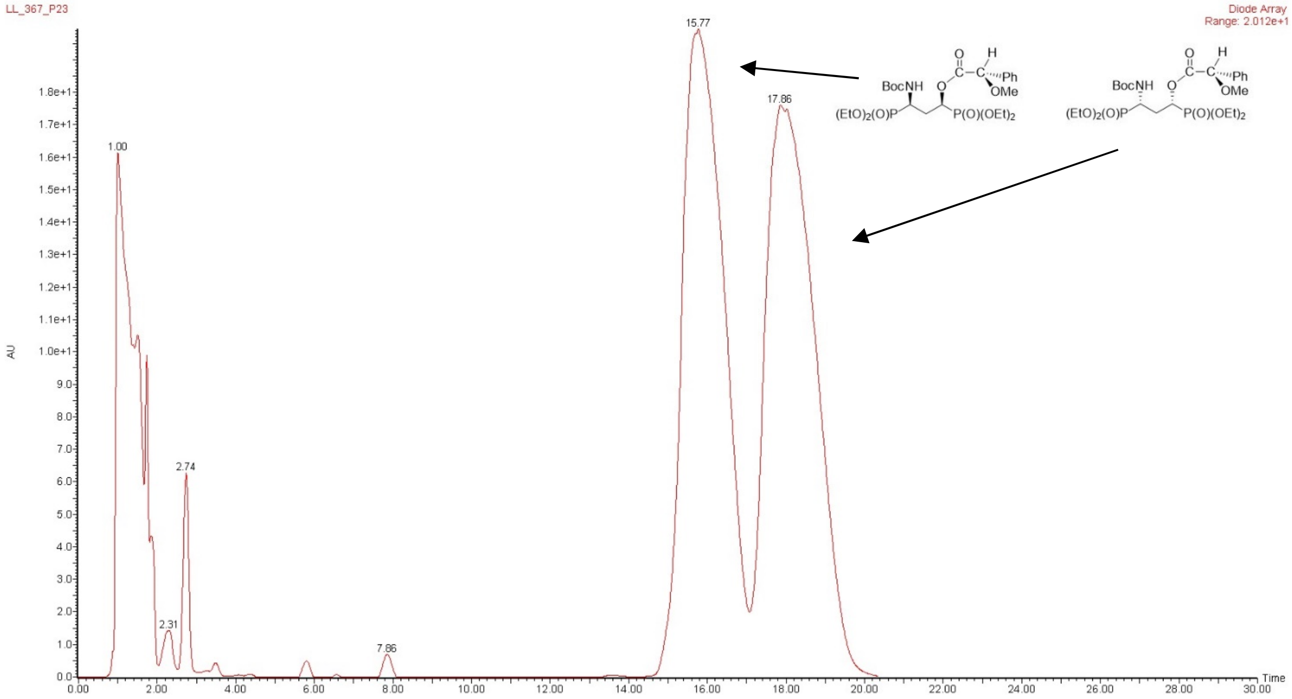

Figure S49: Separation of (1*R*,3*R*,1′*S*)-**16** and (1*R*,3*S*,1′*S*)-**16** by preparative HPLC

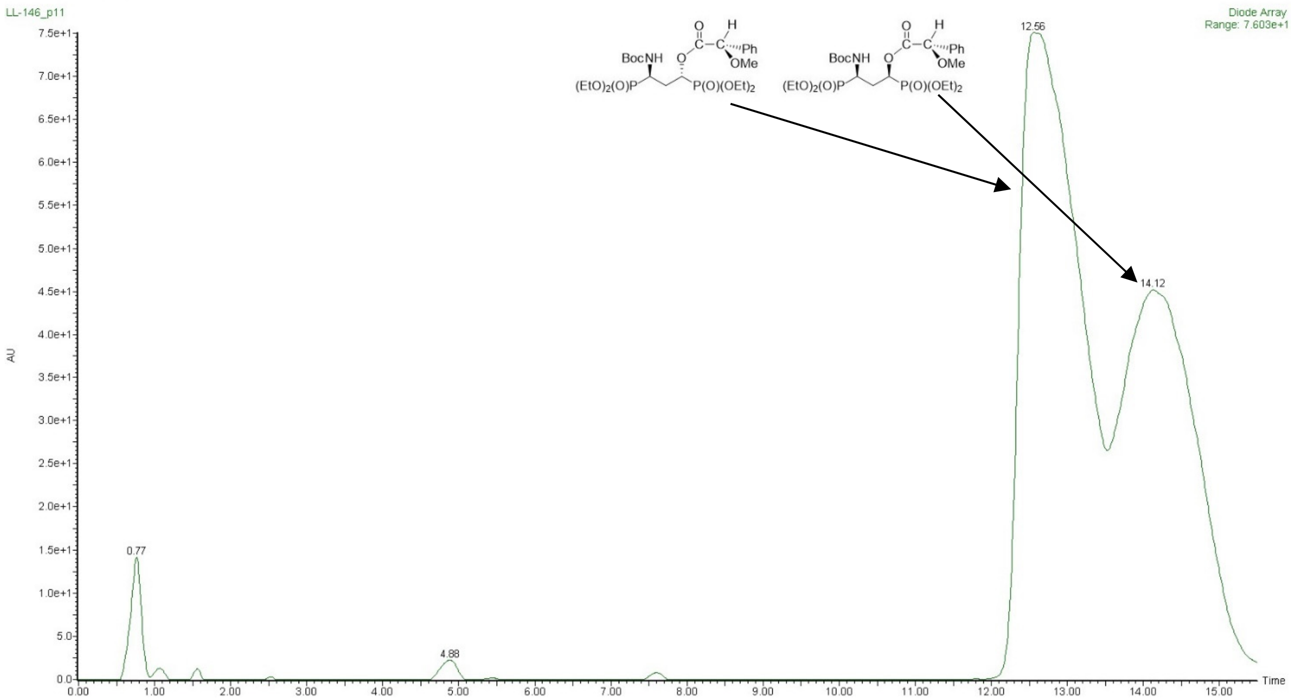

Figure S50: Separation of (1*R*,3*R*,1′*S*)-**16** and (1*R*,3*S*,1′*S*)-**16** by preparative HPLC

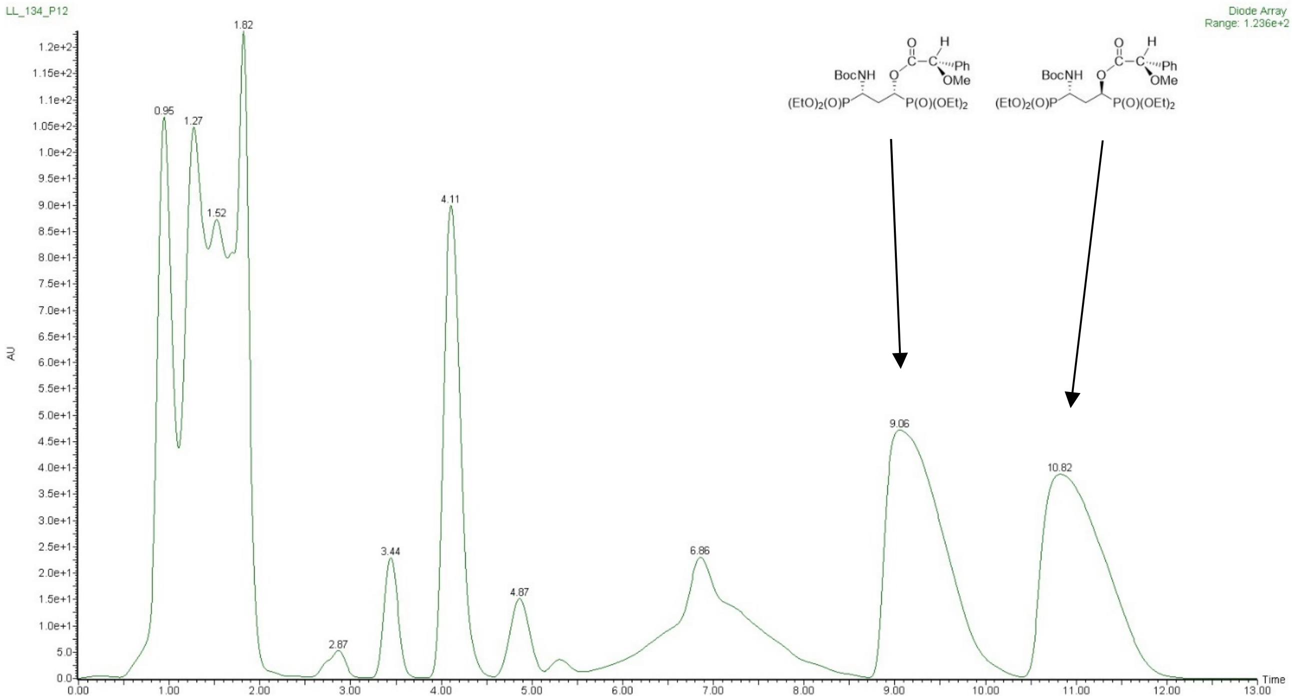

Figure S51: Separation of (1*S*,3*R*,1′*S*)-**16** and (1*S*,3*S*,1′*S*)-**16** by preparative HPLC
